# Somatic mutations distinguish melanocyte subpopulations in human skin

**DOI:** 10.1038/s41556-026-01943-7

**Published:** 2026-04-27

**Authors:** Bishal Tandukar, Delahny Deivendran, Limin Chen, Neda Bahrani, Defne Baskurt, Beatrice Weier, Harsh Sharma, Noel Cruz-Pacheco, Min Hu, Kayla Marks, Rebecca G. Zitnay, Aravind K. Bandari, Barbara Rentroia-Pacheco, Rojina Nekoonam, Boris C. Bastian, Iwei Yeh, Robert Judson-Torres, A. Hunter Shain

**Affiliations:** 1 Department of Dermatology, University of California San Francisco, San Francisco, CA USA; 2 Helen Diller Family Comprehensive Cancer Center, University of California San Francisco, San Francisco, CA USA; 3 Department of Molecular and Cell Biology, University of California Berkeley, Berkeley, CA USA; 4 College of Osteopathic Medicine, California Health Sciences University, Clovis, CA USA; 5 Huntsman Cancer Institute, University of Utah, Salt Lake City, UT USA; 6 Department of Oncological Sciences, University of Utah, Salt Lake City, UT USA; 7 Department of Dermatology, University of Utah, Salt Lake City, UT USA; 8 Department of Biomedical Engineering, University of Utah, Salt Lake City, UT USA; 9 Department of Dermatology, Erasmus Medical Center Cancer Institute, Erasmus University Medical Center, Rotterdam, Netherlands; 10 Department of Pathology, University of California San Francisco, San Francisco, CA USA

**Keywords:** Genomics, Skin stem cells, Cancer genomics

## Abstract

Here, to understand the homeostatic mechanisms governing melanocytes, we interrogate the mutational landscapes, gene-expression profiles and morphological features of 297 clonal expansions of epidermal melanocytes from 31 donors. We show that a population of melanocytes with low mutation burden persists in sun-exposed epidermis. These cells are smaller, less dendritic, and exhibit stem-like expression profiles when compared to melanocytes carrying high mutation burdens. Using single-cell spatial transcriptomics, we show that melanocytes inferred to have low mutation burdens localize to both hair follicles and interfollicular epidermis, whereas melanocytes with high mutation burdens are largely restricted to epidermis. We propose that melanocytes in the hair follicle occupy a privileged niche, protected from ultraviolet radiation, but replenish the epidermis following photodamage. This study highlights the value of incorporating mutational information into cell atlases. Cells can change their positions over time, but mutations provide a historical record of processes that were operative on each cell.

## Abstract (web summary)

Tandukar et al. dissect the mutational, gene expression and morphological features of adult human skin melanocytes at single-cell resolution.

## Main

During embryogenesis, neural crest cells give rise to smooth muscle, bone, connective tissue, neurons and melanocytes^1–3^. Melanocytes are pigment-producing cells that mostly migrate to the skin but can colonize, to a lesser extent, other sites in the body. The developmental trajectory of the melanocytic lineage has predominantly been traced in animal models^1–3^, but there are key differences between melanocytes from humans and other animals. Fish, birds and rodents evolved pigmentation mainly to alter their appearance (for example, for camouflage or mating purposes)^4^, whereas in humans, pigmentation evolved as a defence mechanism against ultraviolet (UV) radiation^5^.

Single-cell RNA-sequencing has helped define the cellular fates of melanocytes in humans^6^. As an example, melanocytes from acral body sites (the non-hair-bearing skin of the foot sole, palm and nail bed) occupy a cellular state distinct from those of the cutaneous melanocytes found elsewhere in the body^6^, probably explaining why acral melanomas are biologically different from cutaneous melanomas^7,8^. RNA-sequencing has highlighted anatomic specificity within melanocytes in humans, but our understanding of their cellular hierarchies and the extent to which external stresses, such as UV radiation, shape their cellular states remains incompletely understood.

**Fig. 1:**
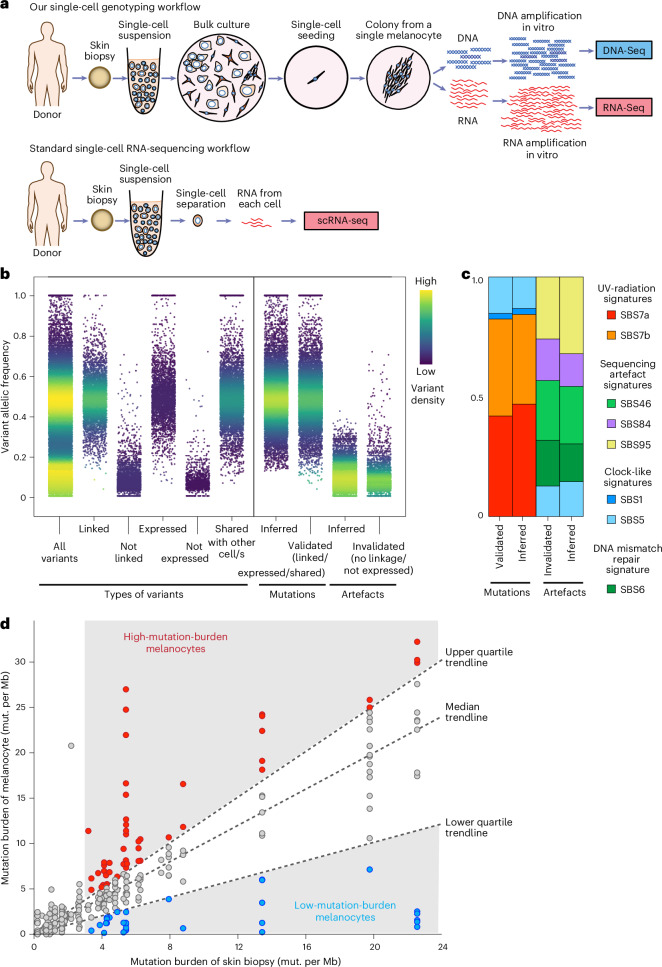
Melanocytes with high and low mutation burdens coexist in human skin biopsies. **a**, An overview of our single-cell genotyping workflow compared to a typical single-cell RNA-sequencing workflow. **b**, Scatterplot showing the variant allelic frequencies (VAFs) of all candidate mutations, aggregated from all melanocytes (*n* = 297) in this study. True somatic mutations were validated when they were detectable in matching RNA-sequencing data from the same cell, in linkage with nearby SNPs, or found in two or more cells from the same donor. For candidate mutations outside expressed genes or far away from SNPs, mutational status was inferred based on their allele frequencies (Methods). **c**, Stacked plot illustrating the mutational signatures of validated mutations, inferred mutations, invalidated artefacts and inferred artefacts. **d**, Mutation burdens (mutations per megabase, mut. per Mb) of individual melanocytes (*n* = 297) plotted against the mutation burdens of the skin biopsies (58 independent skin biopsies of 31 unique donors) from which they were collected. The mutation burden of each skin biopsy is defined as the median mutation burden of all melanocytes from that biopsy. Upper, middle and lower quartile trend lines are shown. Low- and high-mutation-burden melanocytes were gated (Methods) as shown. Note how skin samples with high mutation burdens maintain populations of melanocytes with few mutations. Source data

In this Resource paper, to address this gap in knowledge, we catalogue the mutational, gene expression and morphological features of small colonies of individual melanocytes expanded ex vivo from the epidermal compartment of human skin (Fig. 1a). Cell atlas studies are routinely carried out by performing single-cell RNA-sequencing to define cellular states^9^. Our multi-omic approach has advantages over single-cell RNA-sequencing alone (Supplementary Table 1). Single-cell RNA-sequencing provides a one-dimensional view into the biological state of each cell, and the shallow sequencing depth from these technologies imparts limited resolution into heterogeneity within a cellular lineage. Our work reveals mutationally distinct subpopulations of melanocytes in the epidermis. We also validate their existence and interrogate their spatial distribution with spatial transcriptomics (Xenium Analyzer, 10X Genomics).

## Results

### Phenotyping melanocytes from human skin

We took punch biopsies of sun-exposed adult skin. After incubation in dispase to break down extracellular matrix proteins, the epidermis was physically separated from the dermis with tweezers. With this separation strategy, the epidermal compartment captures melanocytes from the interfollicular epidermis (the portion of the epidermis between hair follicles) and the infundibulum (the opening of the hair shaft)^10^. Melanocytes can also occupy adnexal structures in the dermal compartment, such as the outer root sheath of the hair follicle, but these melanocytes were not captured in our epidermis isolations. Bulk melanocytes were established in tissue culture from the epidermal compartment.

**Extended Data Fig. 1:**
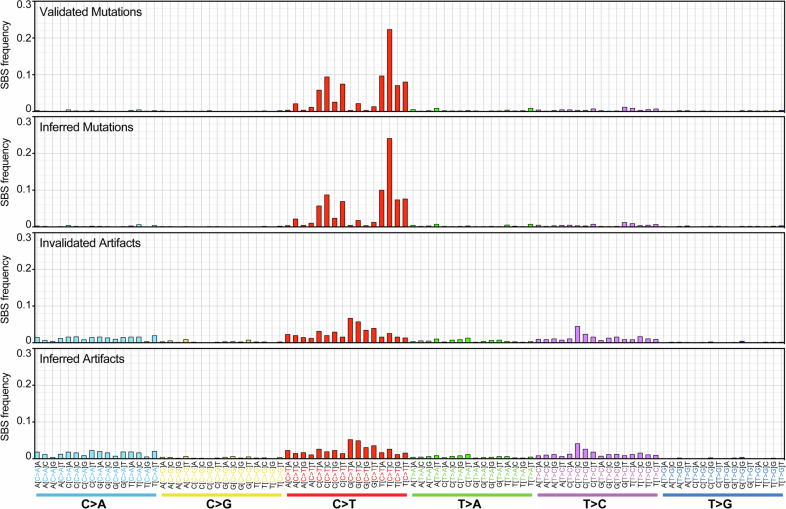
Validation and benchmarking somatic mutation calls from small expansions of skin cells. The 96-bar plots show the frequency of the six possible somatic point mutations across all trinucleotide contexts from: validated mutations, inferred mutations, artefacts, and inferred artefacts. Note that inferred mutations have similar allele frequencies and mutational signatures as validated mutations. Source data

We catalogued somatic mutations in melanocytes by clonally expanding individual melanocytes into small colonies (~230 cells), further amplifying the DNA and RNA of each colony in vitro, and sequencing the amplified nucleic acids (Fig. 1a). We developed a bioinformatic approach^11–13^ to call mutations at high sensitivity and specificity from each expansion of melanocytes (Fig. 1b,c and Extended Data Fig. 1). Because we sequenced both DNA and RNA, there were matching gene-expression data to accompany the mutational information from each colony. The RNA-sequencing data were used to validate mutation calls from DNA-sequencing data and to perform gene-expression analyses (Supplementary Tables 2 and 3). The RNA was sequenced at higher depth than a typical cell atlas study^14,15^, providing greater resolution into potential sublineages of melanocytes. We also photographed each colony to record their morphological features.

A limitation to our approach is the involvement of cell culture, which may alter the gene expression, morphological or, to a lesser extent, the mutational state of each cell. We also characterized fewer cells than a typical cell atlas study. However, these limitations are counterbalanced by the extensive phenotypic information that was collected from each cell’s colony (Supplementary Table 1). In total we profiled 297 cells from 58 independent skin biopsies from 31 unique donors, approximately half of which are previously published^13^, while the remainder are newly sequenced here. This sample size, in the hundreds, is smaller than most single-cell RNA-sequencing studies, but it represents one of the most extensive genomic and transcriptomic resources characterizing the somatic mutational landscapes of melanocytes, and it is comparable in scale to other studies that clonally expand cells from normal tissues for mutational analyses^16–18^.

### Low- and high-mutation-burden melanocytes coexist in the epidermis

The mutation burden of individual melanocytes varied from person to person, site to site within each person, and cell to cell within each site^13^. In this study we focus on the latter source of variability, aiming to understand how melanocytes from the same epidermal biopsy, which would be expected to experience similar exposures to UV radiation, accumulate different levels of mutational damage.

With this goal in mind, we plotted the mutation burdens of individual melanocytes versus the mutation burdens of the tissues from which they were derived (Fig. 1d). Skin samples with high mutation burdens, on average, maintained a subpopulation of melanocytes with low mutation burdens. We defined melanocytes with low (LowMut) and high (HighMut) mutation burdens as those in the bottom and top quartile of mutation burdens. We also restricted our comparisons to melanocytes from tissues whose mutation burden exceeded three mutations per megabase (mut. per Mb). This decision was made due to the fact that it was difficult to separate low- and high-mutation-burden melanocytes when there was little mutational damage in the tissue. The gates defining the high- and low-mutation-burden melanocytes are shown in Fig. 1d.

### Distinct mutational factors operate on melanocytes with high and low mutation burdens

**Fig. 2:**
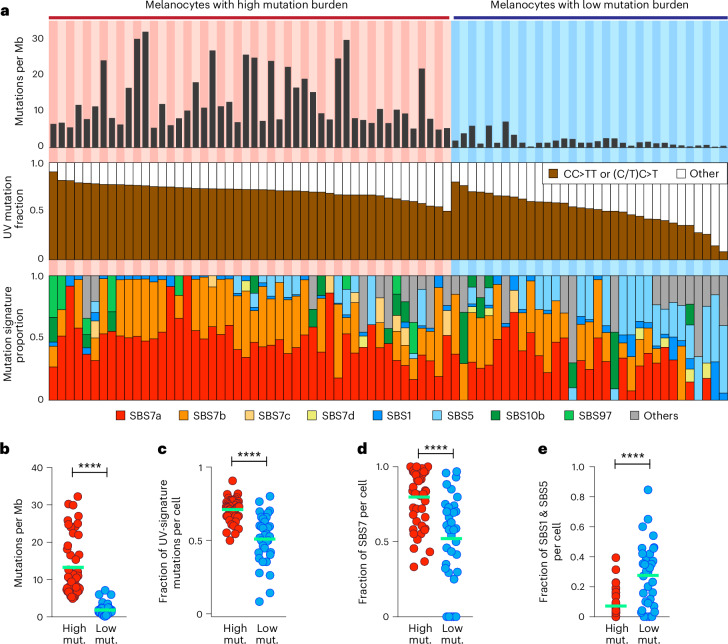
Distinct mutational factors operate on melanocytes with high and low mutation burdens. **a**, Top: mutation burdens of site-matched melanocytes with high and low mutation burdens (see Fig. 1 for a definition of each group). Each column represents one melanocyte. Middle: fraction of UV-radiation-induced mutations in each melanocyte. Bottom: fraction of mutational signatures in each cell. The melanocytes are arranged in descending order by fraction of UV-induced mutations (middle). **b**–**e**, Dot plots summarizing select features in high-mutation-burden (*n* = 48) and low-mutation-burden (*n* = 33) melanocytes, including mutation burden (****P = 1.4 × 10^−13^) (**b**), fraction of UV-radiation-induced mutations per cell (****P = 8.8 × 10^−5^) (**c**), fraction of SBS7-associated mutations per cell (****P = 8.5 × 10^−3^) (**d**), as well as the fraction of SBS1- and SBS5-associated mutations per cell (****P = 2.1 × 10^−7^) (**e**). Green bars represent the mean values for each subset, and each dot corresponds to the value for an individual melanocyte. All comparisons use single cells from separate donors as independent biological units. For all plots, *P* values were calculated using the Wilcoxon rank-sum test (two-sided, cell-to-cell comparisons). Source data

We next characterized the types of mutation in the two populations of melanocytes. Melanocytes with high mutation burdens had a greater proportion of cytosine-to-thymine transitions at the 3′ basepair of dipyrimidines (Fig. 2a–c)—the classic mutation associated with UV radiation^19^. They also had a greater proportion of signature 7 mutations (Fig. 2d)—a signature defined in pan-cancer analyses that is attributable to UV radiation^20^. By contrast, the low-mutation-burden melanocytes had a greater proportion of ‘clock-like’ signatures 1 and 5^20^ (Fig. 2e). These observations imply there is a subpopulation of melanocytes, from the epidermis of heavily sun-exposed skin, which were relatively protected from UV radiation for much of their lives.

### Low- and high-mutation-burden melanocytes have distinct gene-expression profiles

**Fig. 3:**
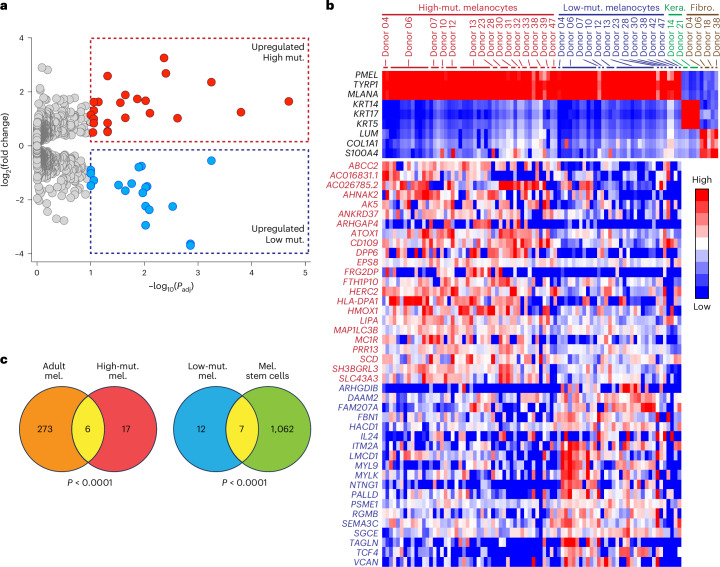
Distinct gene-expression profiles in melanocytes with high and low mutation burdens. **a**, Volcano plot showing differentially expressed genes between melanocytes with high and low mutation burdens using DESeq2. Statistical significance was assessed using a two-sided Wald test, and *P* values were adjusted for multiple comparisons using the Benjamini–Hochberg method. Genes with adjusted *P* < 0.10 were considered significant. **b**, Heatmap showing the expression of genes (rows) in individual skin cells (columns). The top portion compares melanocytes to other cell types in the skin—keratinocytes (*n* = 5) and fibroblasts (*n* = 48)—using selected markers known to be expressed in each lineage. The bottom portion shows differentially expressed genes (from **a**) in melanocytes with high (*n* = 48) and low (*n* = 33) mutation burdens. **c**, Venn diagram showing overlap/non-overlap between genes discovered in our study (the high- and low-mutation-burden genes) and genes discovered by Belote et al.^6^. For HighMut melanocytes, ****P = 3.95 × 10^−8^ versus adult melanocytes and *P* = 0.2569 versus melanocyte stem cells (MSCs). For LowMut melanocytes, *P* = 0.7556 versus adult melanocytes and ****P = 6.02 × 10^−6^ versus MSCs. Statistical significance of overlap was calculated with a one-sided hypergeometric test for enrichment. Source data

**Extended Data Fig. 2:**
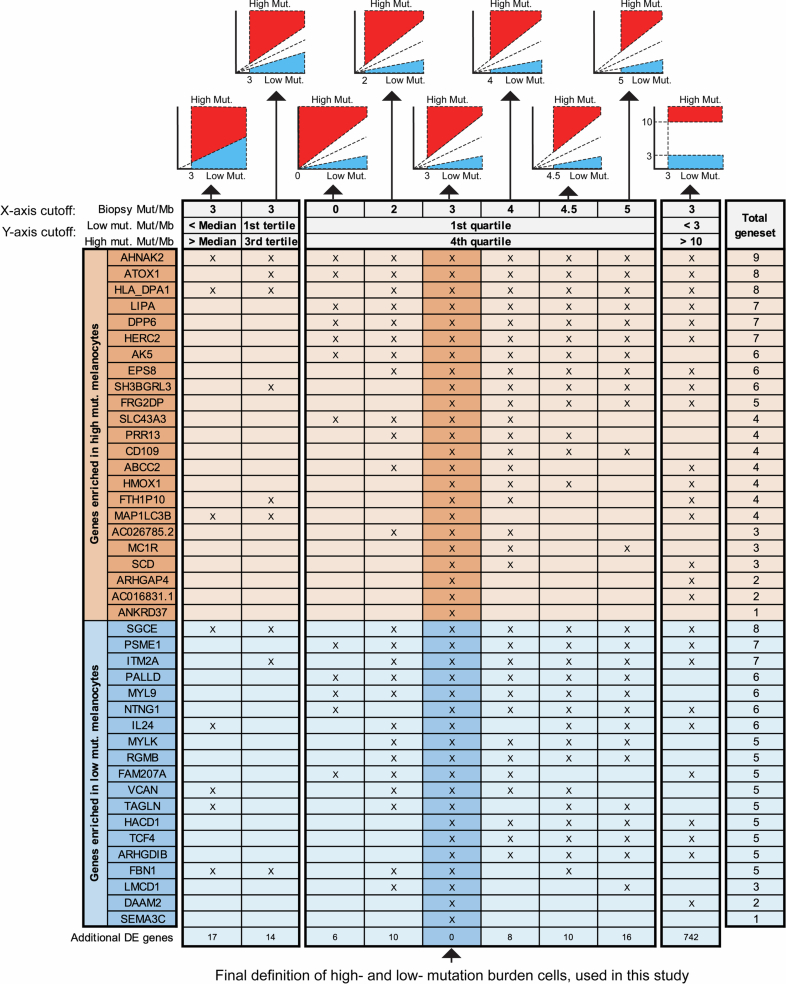
Alternative definitions of high- and low- mutation burden melanocytes capture biologically similar cells. Melanocytes were gated into high- and low- mutation burden cells based on their absolute mutation burden and/or their relative mutation burden within a biopsy. After gating, differential gene (DE) expression analyses were performed, comparing melanocytes with high- (n = 48) vs low- (n = 33) mutation burdens. The differentially expressed genes under our final condition are shown above, and we indicate when these genes were also found with an alternative definition of high- and low- mutation burden melanocytes. The number of additional differentially expressed genes, found with alternative analyses, is indicated in the bottom row. Statistical significance was assessed using a two-sided Wald test, and p-values were adjusted for multiple comparisons using the Benjamini–Hochberg method. Genes with an adjusted p-value < 0.10 were considered significant. Source data

**Extended Data Fig. 3:**
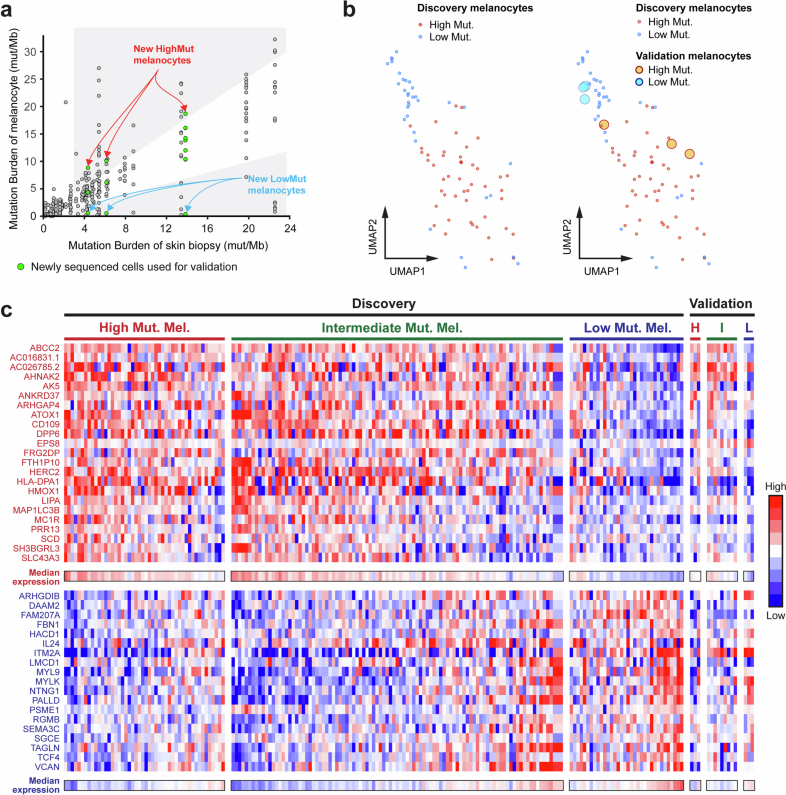
Gene expression profiles of HighMut and LowMut melanocytes are validated in additional cells from new donors. **a**. Each dot represents an individual melanocyte, with its mutation burden (mut/Mb) plotted as shown in Fig. 1. Green dots indicate melanocytes from a new set of three donors, not included in the original analysis. In total, three new HighMut and three new LowMut melanocytes were identified for validation. **b**. UMAP plots of individual melanocytes, stratified by their expression of differentially expressed genes shown in panel **c**. The left panel shows “discovery melanocytes”, and the right panel overlays “validation melanocytes” from the new donors. Note that the new LowMut and new HighMut melanocytes cluster with their respective populations from the discovery cohort. **c**. A heatmap displays gene expression levels (rows) across individual melanocytes (columns). From left to right are HighMut (n = 48), IntermediateMut (n = 99), and LowMut melanocytes (n = 33) from the discovery set; and again from the validation cohort (n = 3, 9, 3). The validation melanocytes exhibit gene expression profiles consistent with those of the discovery set. Source data

**Extended Data Fig. 4:**
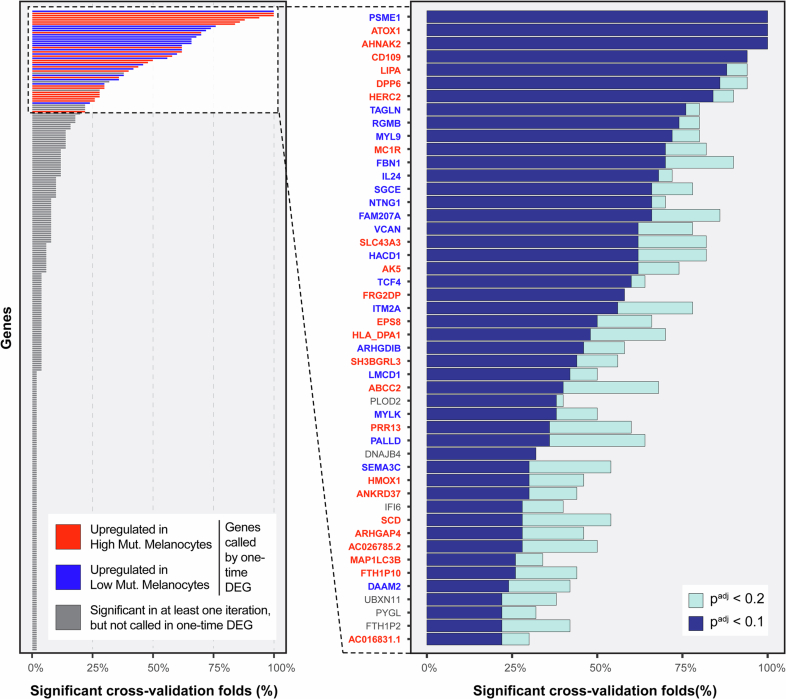
Robustness of HighMut and LowMut gene signatures. The left panel show percentage of cross-validation training folds (50 total: 5 folds × 10 repetitions) in which each gene was significant. Genes upregulated in low- or high-mutation melanocytes are shown in blue and red, respectively. Grey bars indicate genes significant in at least one fold but not called in the one-time analysis. The right panel depicts zoomed view of the top genes most frequently identified. Dark blue bars: FDR-adjusted p < 0.1; light blue bars: FDR-adjusted p < 0.2. All 42 HighMut and LowMut genes from the one-time analysis are among the top 48 most consistently identified, demonstrating the reproducibility and robustness of the gene signatures across folds and repetitions. Source data

To further understand the biological differences between melanocytes with low and high mutation burdens, we performed differential gene-expression analyses (Fig. 3a)^11,21^. Differentially expressed genes were stable, even when adjustments to the definitions of low- and high-mutation-burden melanocytes were considered (Extended Data Fig. 2). Moreover, the gene-expression profiles of melanocytes with high and low mutation burdens were reproducibly observed in a validation cohort from three new donors, not included in the original analyses (Extended Data Fig. 3). To further assess the robustness of the gene signature, we performed repeated fivefold cross-validation (ten repetitions) and found that the majority of signature genes were consistently identified across training folds (Extended Data Fig. 4). These results demonstrate that the HighMut and LowMut gene signatures are reproducible and robust, both across independent donors and across multiple cross-validation splits.

As expected, both groups expressed melanocyte markers at levels that were several orders of magnitude greater than for keratinocytes or fibroblasts (Fig. 3b, upper heatmap), confirming their shared melanocytic lineage despite phenotypic variation within the lineage. The significantly upregulated genes in melanocytes with high and low mutation burdens were compared to the molecular signature database^22^. A subset of notable overlaps (discussed below) are highlighted in Supplementary Table 4, and all gene sets with significant overlap are listed in Supplementary Table 5.

The melanocytes with high mutation burdens expressed higher levels of genes involved in pigmentation and antigen presentation (Fig. 3b and Supplementary Table 4). Examples include *HMOX1*, *ABCC2* and *MC1R*, whose proteins participate in the metabolism and catabolism of pigmentation products. Furthermore, they expressed genes overlapping with previously described immune signatures (Supplementary Tables 4 and 5), including genes whose products are responsible for protein breakdown (*LIPA*, *HMOX1*) and antigen presentation (*HLA-DPA1*).

The melanocytes with low mutation burdens expressed higher levels of genes typically observed in cells from neuronal, smooth muscle and connective tissues (Supplementary Table 4). This was notable because, during development, neural crest cells give rise to each of these cell types in addition to melanocytes. Examples of neural crest genes include *VCAN*, *FBN1*, *PALLD*, *ITM2A* (connective tissue genes); *TAGLN*, *MYL9*, *MYLK*, *SGCE*, *HACD1* (smooth muscle genes); and *SEMA3C*, *TCF4*, *DAAM2*, *RGMB*, *NTNG1* (neuronal genes).

**Extended Data Fig. 5:**
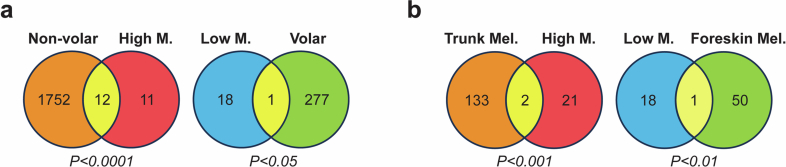
Genes expressed in high- and low- mutation melanocytes show contrasting overlap with gene lists from other studies and contrasting morphology. The Venn diagrams show the overlap of expressed genes (see Fig. 3) in melanocytes with high and low mutation burden with: **a**. genes preferentially expressed in non-volar or volar melanocytes (from Belote et al. Nature Cell Biology, 2021) and **b**. differentially expressed genes from trunk or foreskin melanocytes (from Cheng et al. Cell Reports, 2018). For HighMut melanocytes, ****p = 6.32 x 10-8 vs. non-volar melanocytes, p = 0.3010 vs. volar melanocytes, ***p = 6.55 x 10-4 vs. trunk melanocytes, and p = 0.0632 vs. foreskin melanocytes. For LowMut melanocytes, p = 0.1090 vs. non-volar melanocytes, *p = 3.42 x 10-2 vs. volar melanocytes, p = 0.1330 vs. trunk melanocytes, and **p = 1.31 x 10-3 vs. foreskin melanocytes. Statistical significance of overlap was calculated with a one-sided hypergeometric test for enrichment. Source data

We also compared the genes upregulated in low- and high-mutation-burden melanocytes to gene sets defined in cell atlas studies focused on melanocytes^6,23^ (Fig. 3c and Extended Data Fig. 5). The genes upregulated in our high-mutation-burden melanocytes overlapped with genes upregulated in ‘adult’ melanocytes from ref. ^6^, and the genes upregulated in our low-mutation-burden melanocytes overlapped with ‘melanocyte stem cell’ (MSC) genes from ref. ^6^. The ‘MSC’ gene set from ref. ^6^ was enriched in melanocytes from fetal tissue, and suggested to derive from melanocytes in hair follicles^6^.

**Extended Data Fig. 6:**
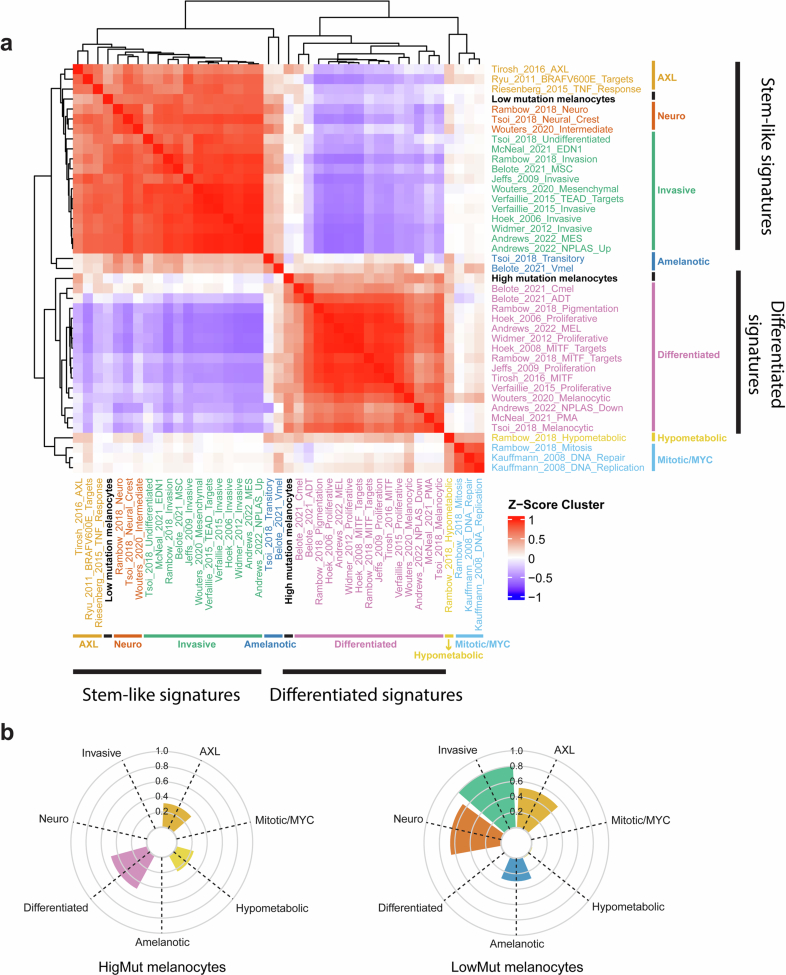
The HighMut and LowMut gene expression signatures are differentially associated with other melanocytic signatures. The WIMMS (What Is My Melanocytic Signature) tool was used to determine how gene expression in high- and low- mutation burden melanocytes correlate with genes expressed in previously published melanocytic signatures. **a**. Previously published signatures are grouped broadly into stem-like and differentiated signatures with subgroups, as indicated. **b**. Our HighMut signature clustered with other differentiated signatures while our LowMut signature clustered with other stem-like signatures. Source data

Finally, we used a recently developed tool, WIMMS (What Is My Melanocytic Signature), to interrogate how our gene-expression signatures relate to other melanocytic signatures (Extended Data Fig. 6)^24^. Briefly, WIMMS was used to re-interpret 39 commonly referenced gene-expression signatures from studies of melanoma or melanocytes, showing that many of the signatures are capturing overlapping cell states. With this framework, our high-mutation-burden melanocytes are most similar to ‘differentiated’ melanocytes from other studies, while our low-mutation-burden melanocytes are most similar to ‘AXL’, ‘neuronal’ and ‘invasive’ melanocytes from other studies.

Taken together, gene-expression analyses suggest that melanocytes with high mutation burdens occupy a relatively differentiated state as compared to low-mutation-burden melanocytes.

### Low- and high-mutation-burden melanocytes have different morphological features

**Fig. 4:**
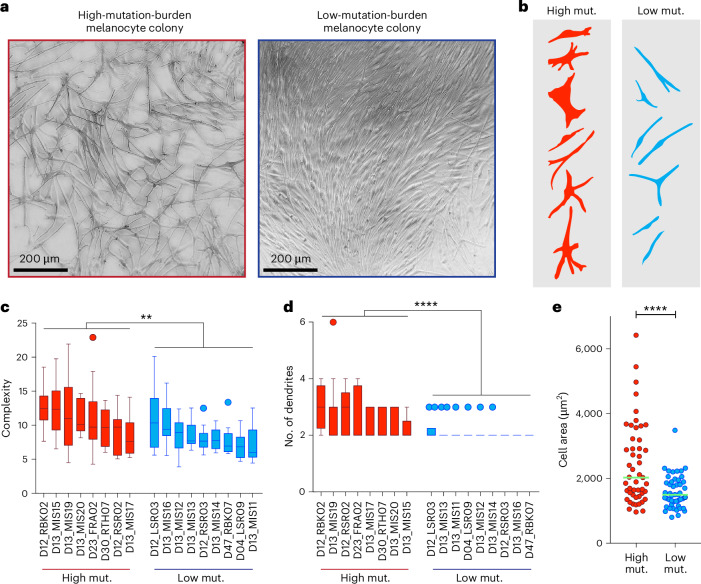
Distinct morphological features in melanocytes with high and low mutation burdens. **a**, Phase-contrast images of a melanocyte colony with high mutation burden (left, 11.27 mut. per Mb) and a melanocyte colony with a low mutation burden (right, 2.45 mut. per MB). The founding melanocytes for each colony came from the earlobe of donor 13. Analysis included 97 HighMut melanocytes from eight independent clonal colonies and 103 LowMut melanocytes from nine independent clonal colonies, all showing consistent morphology across independent experiments. **b**, Masked images of individual melanocytes with high and low mutation burdens, exemplifying the complexity (Methods) of cells with relatively higher mutation burdens. **c**–**e**, Comparison of HighMut and LowMut melanocytes: cell complexity (**P = 3.7 × 10^−3^, comparing the mean complexity per clonal colony; **c**), dendrite counts (****P < 0.0001, comparing the mean complexity per clonal colony; **d**) and cell area in melanocytes (****P = 4.6 × 10^−5^, cell-to-cell comparison; **e**) derived from clonal expansions across multiple donors. For cell complexity and dendrite counts: HighMut, *n* = 97 cells from eight independent clonal colonies; LowMut, *n* = 103 cells from nine independent clonal colonies. For cell area measurements: HighMut, *n* = 49 cells from eight colonies; LowMut, *n* = 47 cells from nine colonies. Independent clonal colonies derived from distinct founding melanocytes represent biological replicates; individual cell measurements within colonies represent technical replicates. In **c** and **d**, box plots are Tukey box plots defined as follows: the centre line represents the median; the bounds of the box indicate the 25th and 75th percentiles (interquartile range, IQR); whiskers extend to the most extreme data points within 1.5 × IQR; data points beyond this range are plotted as individual outliers. In **e**, each dot represents an individual cell. Horizontal green lines indicate the median. Statistical significance was assessed using the Wilcoxon rank-sum test (two-sided, clone-to-clone or cell-to-cell comparisons). Source data

Next, we compared the morphology of melanocytes with high and low mutation burdens (Fig. 4a,b shows representative examples of each). Melanocytes that could be distinguished from their neighbours were segmented, and we calculated their perimeters, surface areas and number of dendritic extensions. Melanocytes with high mutation burdens had more complex morphologies, defined by the ratio of their perimeter to their surface area (Fig. 4c). They also tended to be dendritic and larger (Fig. 4d,e).

Immunostaining for MITF and MLANA (markers for melanocytes) has shown that dendritic melanocytes are enriched in the interfollicular epidermis and the bulb of the hair follicle, while ovoid melanocytes are enriched in the hair bulge^25^, the niche for epithelial and melanocytic stem cells. Because low-mutation-burden melanocytes were less dendritic in tissue culture, more stem-like, and less sun-damaged, we hypothesized that they have spent most of their lives in the bulge or outer root sheath region of hair follicles before migrating to the interfollicular epidermis.

### Spatial transcriptomics reveals the localization of melanocytes with high and low mutation burdens in human skin

**Extended Data Fig. 7:**
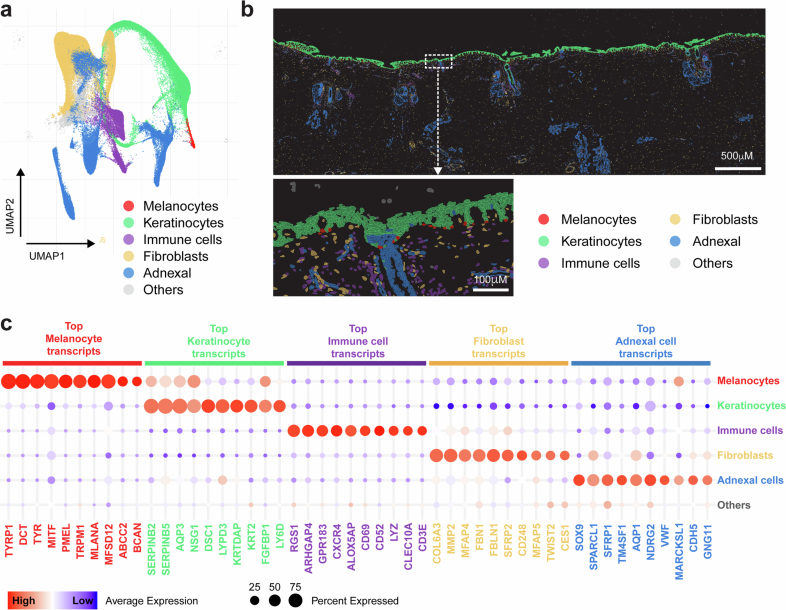
Diverse cell types, including melanocytes, are recognized in Xenium data of normal human skin. **a**. A UMAP, produced by the Xenium Ranger workflow (see methods), shows the relationship of hundreds of thousands of cells from a normal skin biopsy. The graph-based clustering algorithm identified 42 clusters of cells, which were aggregated into six broader groups of cells based on their gene expression profiles. **b**. The localization of different cell types in Xenium data. The epidermis is mainly comprised of keratinocytes with melanocytes occasionally observed in the basal layer. Adnexal structures extend into the dermis, surrounded by a low density of fibroblasts and immune cells. The figure is derived from a skin section of one donor (see Extended Data Figs. 8 and 9 for the additional two donors). **c**. The top 10 genes associated with each group of cells. The melanocytes were distinguished by their expression of known pigmentation genes. Statistical significance was assessed using a two-sided Wald test, and p-values were adjusted for multiple comparisons using the Benjamini–Hochberg method. Genes with an adjusted p-value < 0.10 were considered significant. Source data

**Extended Data Fig. 8:**
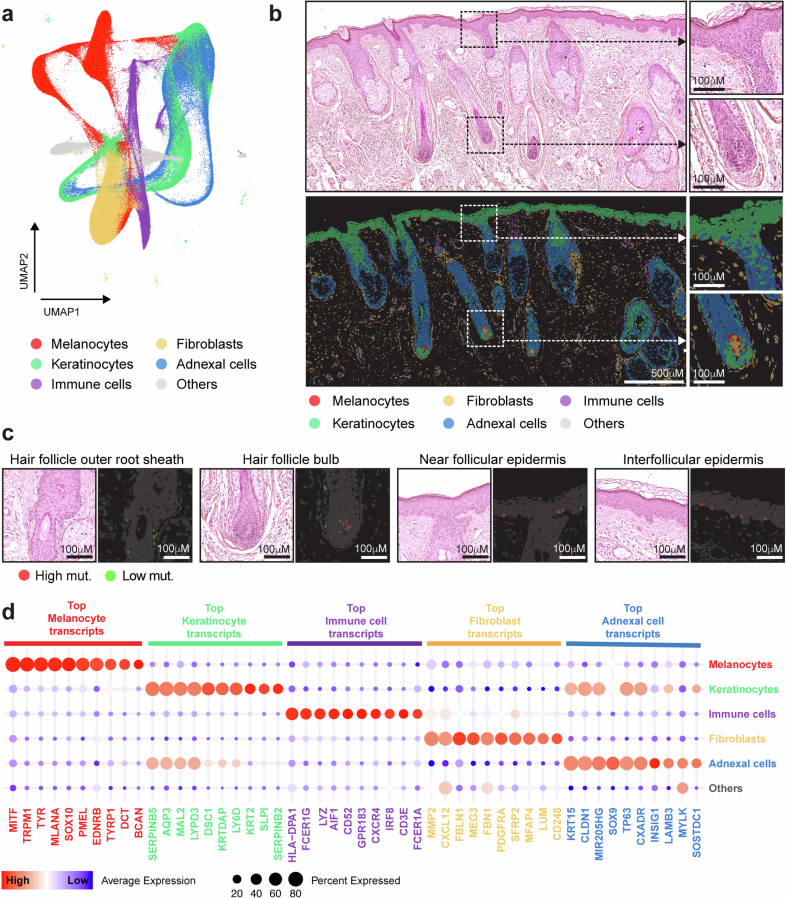
Diverse cell types, including melanocytes, are recognized in Xenium data of human skin from external ear. **a**. A UMAP shows the relationship, based on gene expression patterns, of hundreds of thousands of cells from a skin biopsy of the ear of a 63-year-old female (see Extended Data Figs. 7 and 9 for the additional two donors). The graph-based clustering algorithm identified 41 clusters of cells, which were aggregated into six broader groups of cells based on their gene expression profiles. **b**. The localization of different cell types in Xenium data. **c**. Representative images of melanocytes expressing the HighMut or LowMut gene expression programs in different niches within the skin. The hair follicle bulb melanocytes are identified by their proximity to dermal papillae and internal localization in contrast to outer root sheath melanocytes. **d**. The top 10 genes associated with each group of cells. Statistical significance was assessed using a two-sided Wald test, and p-values were adjusted for multiple comparisons using the Benjamini–Hochberg method. Genes with an adjusted p-value < 0.10 were considered significant. Source data

**Extended Data Fig. 9:**
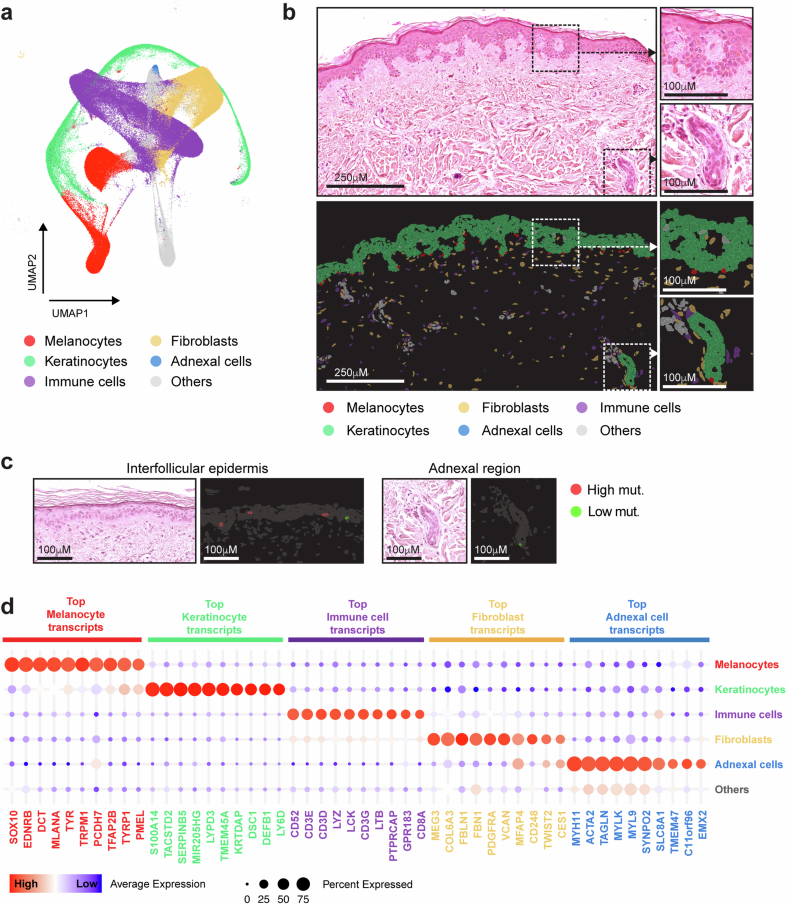
Diverse cell types, including melanocytes, are recognized in Xenium data of human skin from lower back (paraspinal). **a**. A UMAP shows the relationship, based on gene expression patterns, of hundreds of thousands of cells from a skin biopsy of the lower back from a 68-year-old male. The figure is derived from a skin section of one donor (see Extended Data Figs. 7 and 8 for the additional two donors). The graph-based clustering algorithm identified 43 clusters of cells, which were aggregated into six broader groups of cells based on their gene expression profiles. **b**. The localization of different cell types in Xenium data. **c**. Representative images melanocytes expressing the HighMut or LowMut gene expression programs in different niches within the skin. **d**. The top 10 genes associated with each group of cells. Statistical significance was assessed using a two-sided Wald test, and p-values were adjusted for multiple comparisons using the Benjamini–Hochberg method. Genes with an adjusted p-value < 0.10 were considered significant. Source data

To test our hypothesis, we used the Xenium Analyzer (10X Genomics) to interrogate the localization of melanocytes inferred to have high and low mutation burdens in human skin. The Xenium platform measures spatial expression patterns across hundreds of transcripts at subcellular resolution over a large field of view (up to 1 × 2 cm). When choosing probes, we started with a generic skin panel, designed to mark common cell lineages in the skin, and supplemented it with custom targets, which included all genes upregulated in melanocytes with high and low mutation burdens (Supplementary Table 6 provides a list of all 360 genes included on the panel). We analysed formalin-fixed, paraffin-embedded (FFPE) specimens of normal skin adjacent to melanoma from three patients. These normal skin samples exhibited solar elastosis, which—together with the presence of melanoma—reflects a high degree of cumulative sun damage. In total, we measured the localization of ~217 million transcripts across ~800,000 cells. Cell segmentation and clustering distinguished different cell types in the skin (Extended Data Figs. 7–9), but, for the purposes of this study, we focused our analyses on melanocytes.

**Fig. 5:**
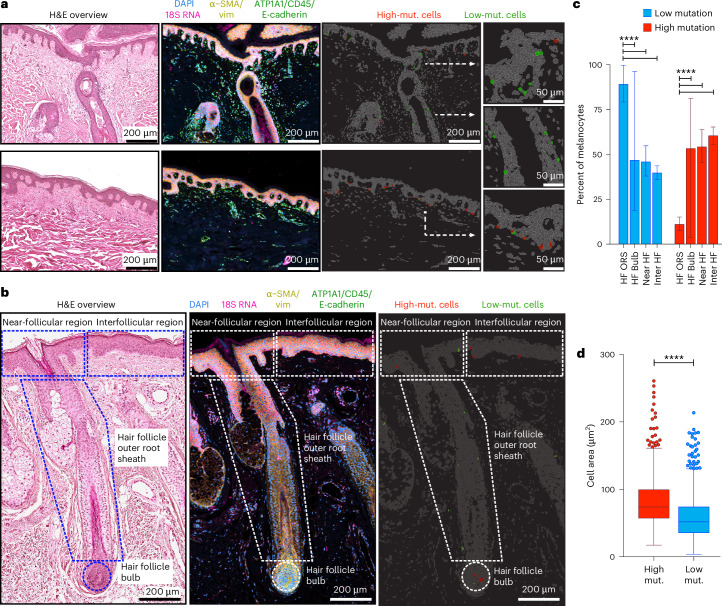
Melanocytes with high and low mutation burdens occupy distinct niches in the skin. **a**, Representative examples from a hair follicle (top panels) and interfollicular epidermis (bottom panels). Each row shows an H&E-stained section (left), corresponding immunofluorescence staining (middle) and Xenium cell segmentations (right). In segmentations, melanocytes expressing genes associated with high mutation burdens are shown in red (HighMut) and those with low mutation burdens in green (LowMut). **b**, Anatomical distribution of melanocytes in normal skin, visualized by H&E (left), immunofluorescence (middle) and Xenium segmentation (right). In **a** and **b**, immunofluorescence panels include nuclear staining (DAPI, blue), cell boundary markers (ATP1A1/CD45/E-Cadherin, pink) and intracellular markers (RNA 18S, yellow; α-SMA/vimentin, green). This multiplexed approach ensured accurate single-cell identification, excluding the possibility of multiple cells being counted as one. LowMut and HighMut melanocytes were enumerated in the hair follicle outer root sheath (HF ORS), hair follicle bulb (HF bulb), near the hair follicle (Near HF) and between the hair follicles (Inter HF). The Xenium analysis was performed on four independent skin sections from three donors, and consistent results were observed across all sections. **c**, Quantification of LowMut (*n* = 810 cells) and HighMut (*n* = 836 cells) melanocytes across defined skin regions from four independent skin sections from three donors. The unit of analysis is the individual melanocyte identified by spatial transcriptomic segmentation. Bars represent the percentage of melanocytes within each region, calculated from aggregated single-cell counts across sections. Error bars indicate 95% confidence intervals (Poisson exact method). Statistical significance was assessed using a two-sided Poisson exact test (****P < 2.2 × 10^−16^ in all six comparisons). **d**, Comparison of melanocyte volume. LowMut melanocytes (*n* = 811 cells) were significantly smaller than HighMut melanocytes (*n* = 837 cells). Each data point represents one cell across four skin sections from three donors. *P* values were calculated using the Wilcoxon rank-sum test (two-sided, cell-to-cell comparisons; ****P = 2.2 × 10^−16^). In the Tukey box plots, the centre line represents the median ± IQR; whiskers = 1.5 × IQR. Source data

In each melanocyte we counted the number of transcripts mapping to genes upregulated in high- versus low-mutation-burden cells, and rank-ordered these cells by their relative expression of these gene sets. Melanocytes with HighMut gene-expression programs were almost exclusively found in the interfollicular epidermis (Fig. 5a–c). Melanocytes with LowMut gene-expression programs were enriched in hair follicles but could be found in the epidermis, too (Fig. 5a–c). When melanocytes with LowMut gene-expression programs were in the epidermis, they were more common in the infundibulum (the ‘opening’) of the hair shaft (Fig. 5b,c). These findings suggest that melanocytes with low mutation burdens originate in the hair follicle, but can migrate to the interfollicular epidermis. We also inferred the area of each cell, and the LowMut melanocytes were relatively smaller (Fig. 5d), consistent with the morphological features observed in vitro (Fig. 4).

Interestingly, a rare population of melanocytes inferred to have high mutation burdens was observed deep in the dermis, positioned above or surrounding the dermal papillae within the hair bulb (Fig. 5b). These cells probably did not carry high mutation burdens, but they shared similar gene-expression profiles. In this region, melanocytes are differentiated and function to deliver pigment to the hair shaft.

## Discussion

In this study, we catalogued mutational, gene-expression and morphological profiles of melanocytes in human epidermis. Next, we used spatial transcriptomics to infer the localization of subpopulations of epidermal melanocytes in intact skin. Among these modalities, mutational information is the least common in cell atlas studies, but it proved to be uniquely informative. Cells can change their transcriptional states and positional coordinates over time, but mutations are like scars, providing a historical record of the homeostatic processes that were operative on each cell. To our surprise, we found a population of melanocytes in the epidermal compartment of sun-exposed skin with remarkably low mutation burdens, raising an important question. How can cells from the epidermis of the same skin biopsy, ostensibly receiving the same doses of UV radiation, accumulate such different mutation burdens?

**Fig. 6:**
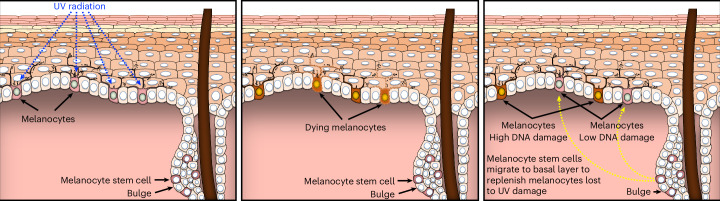
A model to explain how melanocytes with both low and high mutation burdens colonize human skin. Skin is frequently exposed to environmental mutagens such as UV radiation. During repeated waves of UV exposure (left), some epidermal melanocytes undergo cell death as a result of excessive UV-induced DNA damage (middle). In response, MSCs located in the bulge/outer root sheath of hair follicles migrate to the epidermis and replenish the lost melanocytes (right). These newly derived melanocytes harbour relatively low mutation burden. In contrast, epidermal melanocytes that survive repeated UV exposure accumulate a higher mutation burden. We propose that this regenerative process represents a normal homeostatic mechanism that operates continuously in healthy skin to limit the overall accumulation of mutations in epidermal melanocytes.

Our single-cell analyses were performed on melanocytes collected from the interfollicular epidermis. A spatial analysis showed that the interfollicular epidermis houses melanocytes inferred to have both high and low mutation burdens, based on their gene-expression patterns. These analyses also showed that the hair follicle is heavily enriched with melanocytes inferred to have low mutation burdens, based on their gene-expression profiles. We propose that melanocytes with low mutation burdens spend most of their lives in the hair follicle, thus shielding them from the damaging effects of UV radiation, and they migrate to the epidermis in response to UV radiation (Fig. 6). The bulge houses MSCs^26^, and the melanocytes with low mutation burdens retain the morphological and gene-expression features of stem cells. However, it is possible that the migration out of the hair follicle is driven by other cues, such as injury.

In mice, exposure to UV radiation can induce melanocytes to exit the hair follicle and populate the interfollicular epidermis^27^. In humans, patients with vitiligo do not have epidermal melanocytes (a consequence of auto-immunity against those cells), but treatment with UV radiation causes melanocytes in the hair follicles to migrate into the epidermis and repigment the skin^28^. Our work suggests that these waves of migration are not one-off events, only observed in an experimental or disease setting. Instead, they probably occur on a regular basis in physiologically normal skin, perhaps as a mechanism to replenish melanocytes after sun damage (Fig. 6). After many years of sun exposure, the melanocytes in the epidermis have a broad range of mutation burdens, where the mutation burden of each melanocyte is proportional to the time that it resided in the epidermal compartment of the skin.

The possibility that these homeostatic mechanisms could be co-opted for therapeutic intent is intriguing. The mutation burden of tissues is thought to increase with age^29^, but with more research, it may prove feasible to replace heavily mutated cells with fresh cells to rejuvenate tissue. In a separate study^17^, Yoshida and colleagues described a population of bronchial epithelial cells with ‘near-normal’ mutation burdens admixed with heavily damaged cells from the lungs of smokers. They postulated that the epithelial cells with low mutation burdens may have occupied a privileged niche for much of their lives. In barrier organs, such as skin and lung, more work is needed to understand the location of stem-cell reservoirs and how they replenish the cells at the first line of defence. To achieve this goal, cell atlas studies will need to focus on more data modalities than gene expression. Large-scale consortiums have transcriptionally profiled over 85 million cells and counting^9^. Although these studies have undoubtably proven useful, our work illustrates the benefits of fewer cells but more information per cell, including mutational profiles.

## Methods

### Skin-sample collection for single-cell genotyping of melanocytes

A total of 55 skin biopsies were collected from 31 donors across multiple anatomical sites at the University of California, San Francisco (UCSF) and Northwestern University. At UCSF, biopsies were obtained from donor cadavers enroled in the UCSF Willed Body Program for research purposes. Living patients consented to participating in this study through approved protocols from the Institutional Review Board (IRB) of the University of California, San Francisco (22-36678) and the IRB of Northwestern University (STU00211546).

Cadaver tissues were obtained from donors who had provided broad pre-mortem consent, through their living will, permitting the use of their tissues for medical research and/or educational purposes. Donor demographic information, including age, sex and gender, was obtained from preapproved Vital Statistics Information Sheets. The living donors provided self-reported information and consent, in accordance with their IRBs, to publish data on their UV-radiation exposure and various risk factors for skin cancer, including age, sex, ancestry, sun exposure, sunscreen use and tanning-bed use, by completing a questionnaire. Comparable information for UCSF Willed Body Program donors was obtained from their consented Vital Statistics Information Sheets.

The biopsies were collected as either punch or shave biopsies, with diameters of 3 mm or 5 mm. Tissue samples (buccal mucosa, blood or skin biopsies from distinct anatomical sites) were also collected to establish the genome for each donor.

### Tissue culture and clonal expansion of skin cells

Each skin biopsy was first trimmed to remove excess subcutaneous tissue, then incubated in 10 mg ml^−1^ dispase II for 16–18 h at 4 °C. After incubation, the epidermis was separated from the dermis. Melanocytes or MSCs residing in the hair follicle bulge/outer root sheath are not included in the epidermal fraction when a clean dispase-based epidermis and dermis separation is performed, as the bulge region lies below the basement membrane at the dermal interface. A single-cell suspension of the epidermal layer was obtained by incubating the tissue in 0.05% trypsin for 3 min, with intermittent vortexing every 10–15 s.

The resulting single-cell suspension was seeded in CNT40 medium (CELLnTEC) supplemented with 5% antibiotic–antimycotic and cultured at 37 °C with 5% CO_2_ to establish a bulk culture of keratinocytes and melanocytes over the course of a week. Melanocytes were then selectively isolated through limited trypsinization for 3 min, which detaches only the melanocytes, leaving the keratinocytes adhered.

The melanocytes were manually seeded as single cells into 96-well plates using serial dilution, and cultured in melanocyte medium (CNT40) to grow into clonal colonies of ~230 cells. Manual seeding was preferred over fluorescence-activated cell sorting, as it was less abrasive to primary cells and yielded more clonal expansions.

Colonies were monitored daily. Wells containing two or more cells on day 1 were marked as doublets and excluded from further analysis. Similarly, wells in which two physically separate colonies emerged (for example, on opposite sides of the well) were also classified as doublets and not processed. If a doublet was missed during screening, it became evident during mutational analysis, as somatic mutations in those wells showed allele frequencies consistently below 50%. Regular monitoring also allowed us to determine the optimal harvest time—when proliferation began to slow but before cells stopped dividing and underwent growth arrest. The replicative lifespan of melanocytes varied across samples, reflecting differences in intrinsic cell state, biopsy quality, donor age and other factors. Accordingly, colony sizes also varied. These findings align with previous reports showing that telomere shortening, donor characteristics and environmental exposures contribute to differences in melanocyte proliferative capacity^30,31^. Finally, monitoring cell growth also enabled us to confirm cell type by assessing cell morphology.

### Genomic and transcriptomic sequencing of individual melanocytes

The colonies of melanocytes were collected in RLT buffer (Qiagen, 79216). DNA and mRNA were extracted, separated and amplified using the G&T sequencing protocol^32,33^. With this method, the DNA was amplified using either multiple displacement amplification (MDA, Qiagen, 150345) or primary template-directed amplification (PTA) protocol^34^ (BioSkryb, 100136). The mRNA was amplified using the SMART-Seq2 protocol^32,33^. Complementary DNA (cDNA) amplification was achieved using a KAPA HiFi HotStart ReadyMix kit (Roche, KK2502) followed by purification using KAPA HyperPure beads (Roche, 8963843001).

For the reference DNA, bulk DNA was isolated from buccal swabs using the prepIT.L2P method (DNA Genotek, PT-L2P-5) or from unrelated skin biopsies, extracted using the DNeasy Blood & Tissue Kit (Qiagen, 69504).

For library preparation, bulk DNA, melanocyte DNA or cDNA was sheared using a Covaris LE220 instrument to an average fragment size of 350 base pairs. This was followed by end repair, ligation with IDT8 or IDT10 dual index adaptors, and amplification using the KAPA HyperPrep Kit (Roche, KK8504). DNA libraries were then enriched using the KAPA HyperCapture Reagent Kit (Roche, 09075828001).

During the enrichment/hybridization step, probes were used from either the UCSF500 targeted cancer gene panel (developed and validated by UCSF Clinical Cancer Genomics Laboratory, Roche), the NimbleGen SeqCap EZ Exome + UTR panel (Roche, 06740294001) or the KAPA HyperExome V1 panel (Roche, 09062556001). The specific exome probe used for each melanocyte is identified in Supplementary Table 2. Samples were then subjected to paired-end sequencing (100 bp) on either an Illumina HiSeq 2500 or NovaSeq 6000 system, with a total of 297 melanocytes sequenced. For samples with low read counts, rehybridization and additional exome sequencing were performed as needed. A graphical summary of this method is shown in Fig. 1a.

All the nucleic-acid inputs and outputs involving G&T-Seq, library preparation, hybridization and sequencing were quantified using Qubit (dsDNA High Sensitivity quantification), an Agilent Bioanalyzer 2100 (High Sensitivity DNA run) and/or QuantStudio 5 real-time polymerase chain reaction (PCR) system (qPCR with the KAPA Quantification Kit, Roche, KK4854).

### Workflow for sequencing-data analysis and variant calling

DNA-sequencing data were aligned to the hg19 version of the human genome using the BWA-MEM algorithm (v2.0.5)^35^. Following alignment, Picard (v4.1.2.0) (https://broadinstitute.github.io/picard/) was used to deduplicate the genomic reads. The aligned reads were then realigned around indels and recalibrated using the Genome Analysis Toolkit (GATK v4.1.2.0)^36^. For RNA samples, sequencing reads were aligned to both the genome and transcriptome using STAR align (v2.1.0)^37^. Deduplication of RNA reads was performed using Picard (v4.1.2.0). Read counts for each gene were quantified with RSEM (v1.2.0)^38^. Different versions of some of these software programs were tested (https://github.com/ShainLab/Single_Cell_Somatic_Mutation_Caller/tree/main/benchmarking_data) and had little impact on the results.

To identify germline heterozygous single nucleotide polymorphisms (SNPs), FreeBayes (v1.3.1)^39^ was used, and the SNPs were filtered to include only those overlapping with known SNPs in the 1000 Genomes Project^40^ and with allelic frequencies between 40 and 60%. Among other uses, this information was utilized to detect allelic dropout, assisting in the removal of samples with low coverage or amplification biases. A DNAnexus app is available to perform these operations (https://github.com/ShainLab/HaploPrep/tree/main). We also include benchmarking data comparing FreeBayes to another SNP caller (https://github.com/ShainLab/HaploPrep/tree/main/benchmarking_data/VariantCaller_test).

CNVkit (v0.9.6.2) was used to infer copy-number alterations from both DNA- and RNA-sequencing data^41,42^. A reference was used in both modes. When running CNVkit in DNA mode, each cell was compared to a panel of normals from the same sequencing batch. When running in RNA mode, all other cells from the same sequencing batch were used as a reference. Other CNVkit parameters were set to default. As described in greater detail in the documentation, for DNA copy-number inference, CNVkit accepts an interval file of baits and automatically generates ‘bins’ for both on-target and off-target reads. The bin size depends on the bed file of baits. For this study, CNVkit automatically generated bins, resulting in ~250-bp on-target bins and 20-kb off-target bins. For RNA copy-number inference, each gene is treated as its own bin. CNVkit uses circular binary segmentation to segment files by default, producing .cns files of copy-number segments. Heatmaps showing copy-number inferences are available on figshare for each cell (10.6084/m9.figshare.28700804.v2)^43^. Copy-number alterations were rare and therefore not a focus of the present study.

Additionally, a candidate list of short insertions and deletions was generated using Pindel^44^. We elected to use Pindel because it was the choice of the Pan-Cancer Analysis Working Group (PCAWG)^45^. We also benchmarked it against Mutect2, and for calling indels from DNA that had undergone whole-genome amplification we concluded the specificity was superior to Mutect2. The candidate indels were further filtered for a minimum of four reads and variant allelic frequency of 15% or higher. These candidates were visually inspected in an integrative genomics viewer to further remove (1) insertions within homopolymer tracts, which could theoretically be assigned to multiple positions within the tract, and (2) indels occurring adjacent to a germline SNP. These types of false positive indel were previously reported by the PCAWG^45^. The median number of indels per exome was 2, a small proportion of the overall mutation burden, and an extremely small proportion of mutations in this study.

For identifying somatic point mutations^13^, the initial candidate point mutations were called using Mutect2 (v4.1.2.0)^46^. The mutations were further filtered to remove artefacts that arose during whole-genome amplification (https://github.com/ShainLab/Single_Cell_Somatic_Mutation_Caller). The app uses phasing and expression information to validate somatic mutations. True mutations were expected to show linkage to these nearby SNPs, whereas artefacts typically displayed incomplete or no linkage.

For phasing, we begin by using the GATK ReadBackedPhasing function using the patient’s normal BAM file, a VCF of germline heterozygous SNPs (stringently filtered for high specificity), and an annotated VCF file as input. This generated a phased VCF file, which was then used as input for our ‘Single_Cell_Somatic_Mutation_Caller’ custom software application (https://github.com/ShainLab/Single_Cell_Somatic_Mutation_Caller). Within the application, custom scripts split tumour BAM reads into haplotype-specific BAM files. Candidate somatic mutations were then counted in the BAM files corresponding to each haplotype using the samtools mpileup function. These counts were compiled into a summary table, and our app categorized each variant as either clonal (that is, in complete linkage with the nearest germline SNP) or subclonal (not in linkage), or made no call if there were no nearby SNPs and/or coverage was low in the haplotype BAM files.

Second, RNA validation was conducted by verifying the presence of somatic mutations in the RNA-sequencing data, as artefacts were not anticipated to be present in either the genomic or transcriptomic datasets. This approach considers only mutations with sufficient RNA-sequencing coverage and excludes those introducing premature stop codons, which could trigger nonsense-mediated decay. This strategy also does not consider expression of X-chromosome mutations in females, because there is a 50% chance the mutation resides on the silenced allele. After counting the number of reference and mutant reads in matching RNA-sequencing data, our app calls each mutation either ‘expressed’ or ‘not expressed’, or it makes no call if coverage is insufficient, the mutation is truncating, and/or the mutation is on the X chromosome of a female sample.

After validating (or invalidating) candidate mutations within the expressed and phase-able portions of the genome, candidate mutations that do not reside in regions (for example, variants in genes that are not expressed and far away from germline heterozygous SNPs) were inferred to be mutations or artefacts based on their variant allele frequency (VAF). True mutations exhibited a normal distribution centred around 50%, whereas artefacts typically had lower allelic frequencies and fewer overall reads. Cutoff thresholds were set using receiver operating characteristic curves, trained on known mutations/artefacts from each sample, to maximize both sensitivity and specificity (Fig. 1b).

The specificity of mutation calls was benchmarked in several ways, as shown in Fig. 1b. The allele frequencies of mutations were bimodal, with most centred at 50% and a smaller subset at 100%. This pattern is exactly what one would expect for heterozygous and homozygous mutations in cells where copy-number alterations are exceedingly rare. Artefacts tended to have much lower allele frequencies, consistent with their origins in later rounds of whole-genome amplification. Moreover, mutations had expected mutational signatures (ageing or UV radiation), whereas artefacts had known artefactual signatures (Extended Data Fig. 1). These patterns held true whether mutations were inferred from their allele frequencies or validated based on their haplotype distribution, gene expression or presence in other cells.

Once mutations were called, mutation burdens were calculated as mutations per megabase. The total megabases of coverage is denoted the footprint of the genome covered. This was calculated using Footprints software^13^ and includes only nucleotide base pairs with a minimum of 10x or greater coverage.

### Distinguishing melanocyte subpopulations with high and low mutation burden

To identify melanocyte subpopulations with high and low mutation burden (Fig. 1 and Extended Data Fig. 2), we plotted the mutation burden of individual melanocytes versus the mutation burden of the skin biopsy from which they were derived. For this analysis, the median mutation burden of all sequenced melanocytes from each biopsy was considered the biopsy’s overall mutation burden. Linear regression was performed to fit trendlines through the upper and lower quartiles of mutation burdens. Melanocytes falling into the top quartile (Q1) were classified as having a high mutation burden, and those in the bottom quartile (Q4) were classified as having a low mutation burden (Fig. 1d). Only biopsies with a minimum mutation burden of 3 or higher were included, as it is difficult to distinguish subpopulations from biopsies with minimal DNA damage. We considered other definitions of low- and high-mutation-burden melanocytes (Extended Data Fig. 2). Encouragingly, a common set of genes was differentially expressed with the alternate definitions of low- and high-mutation-burden melanocytes.

### Mutation signature analysis between melanocyte subpopulations and variant types

Mutation signatures were inferred for all the cells with a minimum of ten mutations (Supplementary Table 3). The mutations from each melanocyte were compiled to create a mutational profile in the trinucleotide context of single base substitutions (SBSs), with the Bioconductor library BSgenome.Hsapiens.UCSC.hg19 (v1.4.3) using the DeconstructSigs R package (v1.9.0)^47^. Alexandrov and colleagues previously used non-negative matrix factorization to derive 78 unique mutation signatures from 2,780 whole-genome variant calls^20^, which have been widely used in other studies. These signatures, curated by the Wellcome Sanger Institute, are available in the Catalogue of Somatic Mutations in Cancer (COSMIC) database (https://cancer.sanger.ac.uk/signatures/sbs/).

We utilized the SigProfilerAssignment (v0.1.8), which applies a custom forward stagewise algorithm to assign the mutational profile of each cell based on these predefined COSMIC signatures (v3.4)^48^. Figure 2a shows the top eight mutation signatures observed across all cells, including UV-induced signatures. All remaining mutation signatures present in fewer than 10% of the cells were grouped as ‘others’. The overall SBS7 fraction for each melanocyte (Fig. 2d) was calculated by summing all four SBS7 signatures (SBS7a–d).

Mutation signature analysis was also performed to evaluate the quality of mutation calls. In Fig. 1c and Extended Data Fig. 1, we combine variants from all cells that we considered to be mutations or artefacts. Mutations and artefacts were further subdivided into those that were ‘validated’ (by their haplotype distribution and expression patterns) or ‘inferred’ (by their allele frequency). In general, somatic mutations were characterized by signatures expected to operate in skin, namely aging (SBS1 and SBS5) and UV radiation (SBS7a,b) (Fig. 1c). By contrast, artefacts had mutational signatures corresponding to known artefactual signatures (SBS46, 84 and 95) (Fig. 1c). When looking at the 96 bar plots, validated and inferred mutations occur in nearly identical trinucleotide contexts (Extended Data Fig. 1).

### Analysis of gene-expression profiles between melanocyte subpopulations

Differential gene-expression analysis was conducted using the DESeq2 R package (v1.38.3)^11^. RNA reads from the RSEM file were first normalized and transformed using variance-stabilizing transformation. A significance threshold of 10% adjusted *P* value (Benjamini–Hochberg) and a log_2_(fold change) greater than 0 was applied to identify differentially expressed genes. To visualize these genes, a volcano plot (Fig. 3a) and a heatmap (Fig. 3b) were generated. Additionally, gene expression in the two melanocyte subpopulations was compared to that of keratinocytes and fibroblasts using cell type-specific markers (*PMEL*, *TYRP1* and *MLANA* for melanocytes, *KRT14*, *KRT17* and *KRT5* for keratinocytes and *LUM*, *COL1A1* and *S100A4* for fibroblasts; Fig. 3b) to confirm cell identity.

Genes enriched in melanocytes with high and low mutation burdens, as identified through differential expression analysis, were compared to enriched genes from three additional datasets: (1) adult melanocytes versus MSCs, (2) volar versus non-volar melanocytes and (3) foreskin versus trunk melanocytes. Datasets 1 and 2 were obtained from ref. ^6^, and dataset 3 from ref. ^23^. The hypergeometric test was performed on overlapping genes (Supplementary Table 7) in R using the phyper function as follows:

$${\rm{\$}}\,{\rm{phyper}}({\rm{o}},{\rm{a}},{\rm{b}},{\rm{x}},{\rm{lower}}.{\rm{tail}}={\rm{FALSE}},\log .{\rm{p}}={\rm{FALSE}})$$

Here, ‘o’ represents the number of overlapping genes, ‘a’ is the number of enriched genes in the first set, ‘b’ is the number in the second set, and ‘x’ is the number of remaining genes from the total platform of 18,000 genes. Statistical significance was based on the upper tail of the distribution (lower.tail = FALSE). The overlaps were shown as Venn diagrams (Fig. 3c, Extended Data Fig. 5a and Extended Data Fig. 5b).

We further analysed the overlap between the enriched gene sets from (1) high-mutation-burden melanocytes and (2) low-mutation-burden melanocytes with the annotated gene sets in the Molecular Signatures Database (MSigDB) using the Gene Set Enrichment Analysis (GSEA)^22^ tool. Specifically, we compared these overlaps with the following annotated human gene sets: H (hallmark gene sets), C1 (positional gene sets), C2 (curated gene sets), C3 (regulatory target gene sets), C4 (computational gene sets), C5 (ontology gene sets), C6 (oncogenic signature gene sets), C7 (immunologic signature gene sets) and C8 (cell type signature gene sets)^22,49^. Overall, our gene sets were compared to 33,591 MSigDB gene sets, comprising 42,499 genes. The results are presented as the number of overlapping genes per gene set, with statistical significance indicated by *P* values and *Q* values (false discovery rate). All gene sets that showed significant overlap with either the high- or low-mutation-burden melanocyte subsets are listed in Supplementary Table 5.

### Validation of the gene-expression profiles of the melanocyte subpopulations

To validate the gene-expression profiles of HighMut and LowMut melanocytes, we collected additional biopsies (*n* = 3) from the shoulders of three new donors (details are provided in Supplementary Table 2). From these biopsies, 15 melanocytes were sequenced using the same approach as for the main (discovery) cohort. Applying identical cutoff thresholds and selection criteria, melanocytes with high and low mutation burdens were identified (Extended Data Fig. 3a). Enrichment of HighMut- and LowMut-associated genes from the discovery cohort was assessed in the validation cohort by uniform manifold approximation and projection (UMAP) clustering of differentially expressed genes (Extended Data Fig. 3b) and heatmap comparison (Extended Data Fig. 3c).

The robustness of the gene signature was further assessed using repeated fivefold cross-validation with ten repetitions. In each training fold (80% of the cells) of every cross-validation split, we performed differential gene-expression analysis with DESeq2 and identified the significant genes, using the same settings and significance threshold (Benjamini–Hochberg false discovery rate-adjusted *P* < 0.1) applied to derive the final gene signature. To reduce variability due to a single cross-validation split, the entire fivefold cross-validation procedure was repeated ten times, resulting in 50 separate differential expression analyses (10 repetitions × 5 folds). We then plotted the percentage of training folds in which each gene was deemed significant. Of 9,989 genes, 300 were significant in at least one training fold. The 42 genes that we defined as HighMut and LowMut genes, based on a one-time differential gene-expression analysis of all cells, were all among the top 48 genes most frequently discovered across cross-validation training folds (Extended Data Fig. 4).

### Comparing cell morphology between melanocyte subsets

Cell images were captured using either a Zeiss Axiovert 40 CFL instrument or the Invitrogen EVOS FL system. Greyscale images were extracted with Fiji, and brightness and contrast were adjusted to enhance cell visibility (Fig. 4). The ‘rolling ball’ algorithm-based background subtraction was used to correct unevenly illuminated background.

### Cell segmentation and analysis

Raw in-focus images were processed by researchers blinded to mutational status. Briefly, brightfield images were imported into QuPath. The cells that could be distinguished from their neighbours were manually segmented using the polygon tool (4–15 cells per field of view). Annotation measurements were exported, and complexity was calculated from a perimeter-to-area ratio following the method from ref. ^50^. Additionally, the number of dendrites, or projections from the cell body, were manually counted for each segmented object. Measurements were analysed in GraphPad Prism. A representative selection of regions of interest (ROIs) was exported to Fiji and arranged using the ROI manager. The masks of the cells were filled and sorted based on high and low mutation. The complexity and dendrite count of representative melanocytes from clonal expansions of melanocytes with high and low mutation burdens were visualized in a box-and-whisker plot using Tukey’s method, with significance calculated by the two-sided Wilcoxon rank-sum test (Fig. 4c,d).

### Spatial transcriptomic profiling using the Xenium platform

#### Sample selection and sectioning

We obtained FFPE skin samples from three different donors (detailed in Supplementary Table 8). The skin samples were each adjacent to melanoma. Regions containing only normal skin were identified by a dermatopathologist and used for downstream analyses.

A 5-µm section of the skin was mounted on a Xenium slide, with the skin sections positioned within the sample area (10.45 mm × 22.45 mm). Care was taken to ensure that none of the fiducial markers were covered, and any obstructions were manually removed to expose all markers correctly.

Before mounting sections on the Xenium slides, some were first mounted on standard slides and stained with haematoxylin and eosin (H&E) to verify the integrity of the tissue. Sections were also subjected to high-sensitivity eukaryotic Pico RNA analysis (Agilent, 5067-1513) using an Agilent Bioanalyzer 2100. The DV200 score was confirmed to be within the acceptable range for the Xenium run (50–70%).

#### Gene panel design

The targeted gene panel was based on the predesigned human skin panel (v1), which was composed of 260 genes. An additional 100 genes were incorporated to create a custom panel. These additional genes include those enriched in high-mutation melanocytes (*n* = 20), low-mutation melanocytes (*n* = 15), adult melanocytes (from ref. ^6^; *n* = 12), neonatal melanocytes (from ref. ^6^; *n* = 9), fetal melanocytes (from ref. ^6^; *n* = 3), MSCs (from ref. ^6^ and other reports; *n* = 12), differentiated melanocytes (*n* = 6), transitional melanocytes (*n* = 3), neural crest cells (*n* = 17)^51–63^, hair follicle bulge cells (*n* = 2)^26,64^ and Schwann cells (*n* = 1)^65^. Of the 23 genes enriched in high-mutation melanocytes and 19 genes in low-mutation melanocytes, some of them were already included in the predesigned skin panel. The genes *FRG2DP* and *FTH1P10* were excluded due to the lack of suitable probes. The genes are listed in Supplementary Table 6.

Although most custom-added genes had the maximum probe set coverage (up to eight probes), some were reduced to prevent optical crowding. Additionally, the panel includes 40 negative control codewords to assess the specificity of the decoding algorithm and 20 negative control targets to evaluate the assay’s specificity.

#### Sample preparation for the Xenium assay

The slides were processed according to the 10X Genomics recommended protocol. Both slides were handled concurrently using the Xenium slides and sample prep reagents (10X Genomics, PN-1000460). First, the slides were assembled into cassettes (10X Genomics, PN-1000566). Next, the paraffin was removed from the slides, followed by rehydration and decrosslinking of the tissue sections to release sequestered mRNA. Subsequently, probes were hybridized to the target mRNA in the skin sections at 50 °C for 16–24 h. After hybridization, excess probes were washed off during a post-hybridization wash for 30 min at 37 °C. The probes consist of three regions: two regions at the ends that hybridize to the target RNA, and a middle region that encodes the gene-specific barcode.

Ligation of the probes to the target RNA forms a circular DNA probe structure. This is accomplished with a 2-h incubation at 37 °C. Following this, rolling circle amplification is performed for 2 h at 30 °C, generating multiple copies of the gene-specific barcode for each RNA target.

#### Cell segmentation staining

To accurately identify the borders of each cell, immunofluorescence staining was performed using various cell structure markers, including nuclear stain (4′,6-diamidino-2-phenylindole, DAPI), boundary stains (ATP1A1, CD45, E-cadherin) and interior stains (α-SMA, vimentin,18S RNA) (Fig. 5a,b). The staining was carried out by incubating the slides at 4 °C for 16–24 h using Xenium cell segmentation staining reagents (10X Genomics, PN-1000661). After incubation, the stain was enhanced by adding the Xenium staining enhancer reagent, followed by washing to remove excess staining reagents. Autofluorescence was then quenched to reduce background noise. The final step in the staining process involved nuclear staining with DAPI (Supplementary Table 8).

#### Xenium onboard analysis

The slides were processed using the fully automated Xenium Analyzer. The skin samples were imaged over 15 cycles, with fluorescent probes targeting mRNA sequences and other reagents added, imaged and removed during each cycle. The image sensor captured three-dimensional data across four fluorescence channels and multiple fields of view (FOVs) throughout these cycles. The resulting images were stitched together to create a comprehensive spatial dataset for Xenium analysis.

The Xenium Analyzer then processed and decoded the transcript data using its onboard probabilistic model via the Xenium onboard analysis (XOA) pipeline (v3.0.2.0). This decoding utilized predefined fluorescent signal patterns, or codewords, to identify transcripts, with negative codewords included to ensure specificity. Once decoded, the transcripts were deduplicated, and the final data underwent secondary analysis to produce the XOA output. The analysis summary provided information on decoding accuracy, cell segmentation, gene expression and image quality control (QC). All the outputs were visualized using Xenium Explorer (3.1.0).

#### Post Xenium H&E staining of skin samples

Taking advantage of the non-destructive Xenium workflow, the skin-sample slides were H&E-stained post the analysis run. During cell segmentation staining, the autofluorescence quenching leaves behind a purple stain. The slides were therefore first removed from their cassettes, and quencher removal was performed using 10 mM sodium hydrosulfite (Sigma-Aldrich, 157953-5G). The skin sections were then stained with H&E, dehydrated and coverslipped for imaging. The samples were scanned at ×20 magnification using a Leica Aperio CS2 scanner. The output was processed using QuPath bioimage analysis software to extract a pyramidal, tiled ‘OME.TIF’ image file. This image was then imported into Xenium Explorer and manually aligned using a best similarity transformation method. The alignment relies on key points identified from both the XOA spatial output and the H&E-stained image.

#### Spatial analysis of high- and low-mutation melanocytes

Xenium was performed on four sections of skin (sequential sections of donor 1 and one section each from donors 2 and 3). Decoding of the four skin sections yielded 84.4% (48,418,166), 84.9% (46,940,134), 93.1% (62,571,678) and 88.2% (58,915,793) high-quality gene transcripts, with cell segmentation detecting 145,489 to 305,349 cells. Segmentation was performed with the Xenium Analyzer based on boundary staining, RNA abundance and by nuclear (DAPI) expansion. QC values for all the sections analysed by Xenium spatial transcriptomics are listed in Supplementary Table 8.

The XOA pipeline identified distinct clusters in the skin samples based on gene-expression profiles. We categorized the clusters more broadly into five groups after consulting with dermatopathologists and confirming their lineages from gene-expression profiling. The five broad categories include melanocytes, keratinocytes, immune cells, adnexal cells, fibroblasts and an ‘others’ category. The ‘other’ category includes rare cell populations that do not neatly fit into the other categories but may also include apoptotic cells and unevenly segmented cells. The reannotated clusters were visualized using a UMAP projection (Extended Data Figs. 7–9). A total of 5,340 melanocytes were detected in all sections (Supplementary Table 8).

Differential gene-expression analysis was performed, comparing the six categories of cells, outlined above, using the Seurat R toolkit (v5.1.0)^66^ (Extended Data Figs. 7–9). The thresholds for this analysis were an adjusted *P* value (Benjamini–Hochberg) of 0.05 and a log_2_(fold change) greater than 1. The top ten genes enriched in each cell type cluster were visualized as a dotplot heatmap, with upregulated genes marked in red, and the size of the dot indicating the fraction of cells expressing the gene.

To identify melanocytes with high and low mutation burdens, a read count matrix for all genes in the melanocyte clusters was generated from XOA outputs (matrix.mtx.gz, barcodes.tsv.gz and features.tsv.gz). A delta count (the difference between the sum of reads for genes enriched in high-mutation melanocytes and those enriched in low-mutation melanocytes) was calculated for each cell, and cells were rank-ordered based on their relative expression of ‘HighMut’ versus ‘LowMut’ genes. The top 15th percentile of cells were classified as high-mutation melanocytes, and the bottom 15th percentile as low-mutation melanocytes. Using this approach, 821 high-mutation melanocytes and 846 low-mutation melanocytes were identified in all samples (Supplementary Table 8).

After identifying the two melanocyte subpopulations spatially, we quantified their counts in four distinct regions of the skin sample based on proximity to hair follicles: follicular, near-follicle, interfollicular regions and hair bulb (Fig. 5). Two melanocytes (two of 1,648 cells) that did not fall within the defined regions were excluded from the cell-distribution analysis. The results are presented as a bar graph with a 95% confidence interval, and significance was determined using the Poisson test. We also measured the surface area of each melanocyte in the two subpopulations. The surface area data were visualized in a box-and-whisker plot using Tukey’s method, with significance calculated using Tukey’s post-hoc honest significant difference test.

### Statistics and reproducibility

The initial discovery cohort comprised 297 melanocytes derived from 55 skin biopsies collected from 31 donors across multiple anatomical sites, which provided sufficient power to define melanocyte subpopulations and associated gene-expression signatures. All melanocytes meeting predefined QC criteria (including sequencing coverage thresholds and allelic dropout assessment) were included in downstream analyses. No data were excluded. No statistical methods were used to predetermine sample sizes, but our sample sizes are similar or larger than those reported in previous publications^11–13^.

Experiments were not randomized for the initial cell population cohort (HighMut versus LowMut melanocytes), which was generated in an unbiased manner based on distinct mutational profiles. Validation experiments within each cohort were randomized (see Methods for details). Morphologic analyses were performed by researchers blinded to mutational status. Key findings were reproducible across independent donors. The gene-expression signature distinguishing high- and low-mutation melanocytes was validated in an independent cohort of melanocytes sequenced from three additional donors and further supported by repeated fivefold cross-validation with ten repetitions. Detailed validation procedures are described in the Methods.

All statistical tests were two-sided unless otherwise specified. Exact statistical tests, sample sizes, thresholds and bioinformatic tools are provided in the corresponding Methods sections.

### Reporting Summary

Further information on research design is available in the Nature Portfolio Reporting Summary linked to this Article.

## Online content

Any methods, additional references, Nature Portfolio reporting summaries, source data, extended data, supplementary information, acknowledgements, peer review information; details of author contributions and competing interests; and statements of data and code availability are available at 10.1038/s41556-026-01943-7.

## Supplementary information

Reporting Summary

Peer Review File

Supplementary Table **Supplementary Table 1** The table summarizes the advantages and disadvantages of an approach of measuring mutational, gene expression and morphological features from small expansions of individual melanocytes, compared with a standard single-cell RNA-sequencing workflow. **Supplementary Table 2** List of individual melanocytes, keratinocytes and fibroblasts sequenced for this study, along with a summary of their clinical, cytological, genomic and transcriptomic data metrics. **Supplementary Table 3** List of point mutations identified in all the individual melanocytes from the high- and low-mutation subsets. **Supplementary Table 4** Representative molecular signatures associated with high- and low-mutation-burden melanocytes. Select gene sets (see **Supplementary Table 6** for a full list) from the molecular signature database that overlap with high-mutation-burden genes or low-mutation-burden genes. **Supplementary Table 5** List of gene sets from the Molecular Signatures Database (MSigDB) that exhibited significant overlap with high- and low-mutation melanocytes. **Supplementary Table 6** List of genes used for Xenium spatial transcriptomics analysis, including the Xenium skin panel (v1) and the custom panel, along with the corresponding cell types they identify. **Supplementary Table 7** List of genes enriched in melanocytes from adult, melanocyte stem cells, foreskin, trunk, volar and non-volar origins, along with their overlaps with genes enriched in melanocytes with high- and low-mutation burdens. **Supplementary Table 8** Donor information for samples included in the Xenium analysis and quality control (QC) metrics of the Xenium run. **Supplementary Table 9** Light microscopy reporting table.

## Source data

Source Data Fig. 1 Statistical source data.

Source Data Fig. 2 Statistical source data.

Source Data Fig. 3 Statistical source data.

Source Data Fig. 4 Statistical source data.

Source Data Fig. 5 Statistical source data.

Source Data Extended Data Fig.1 Statistical source data.

Source Data Extended Data Fig. 2 Statistical source data.

Source Data Extended Data Fig. 3 Statistical source data.

Source Data Extended Data Fig. 4 Statistical source data.

Source Data Extended Data Fig. 5 Statistical source data.

Source Data Extended Data Fig. 6 Statistical source data.

Source Data Extended Data Fig. 7 Statistical source data.

Source Data Extended Data Fig. 8 Statistical source data.

Source Data Extended Data Fig. 9 Statistical source data.

## Data Availability

Single-cell DNA- and RNA-sequencing data from human skin that support the findings of this study have been deposited in dbGaP under accession codes phs001979.v1.p1 and phs003683.v2.p1. These accession numbers provide access to the raw sequencing FASTQ files. Access to these datasets is restricted because participant consent permits data use only for biomedical research and does not allow unrestricted public release of individual-level genomic information. Investigators can request access through the dbGaP Data Access Committee via the dbGaP portal, and approved users receive data under institutional approvals and data use agreements consistent with the original consent. Requests are typically reviewed within 4–8 weeks, and data remain available for the duration of the repository’s retention policy. Intermediate levels of analysis are also provided. Somatic mutation calls for single cells are included in Supplementary Table 3 and deposited in cBioPortal (https://www.cbioportal.org/study/summary?id=normal_skin_melanocytes_2024). Xenium spatial transcriptomics datasets from skin tissue sections are available in GEO under accession code GSE286964. Additional processed results are provided in the Supplementary Information. These data will also be accessible through the HTAN data portal after the next data release (currently anticipated for fall of 2026). Publicly available gene-expression datasets used for comparison of melanocyte subsets with the HighMut and LowMut cells identified in this study were obtained from previously published studies. Single-cell RNA-sequencing data from Belote et al.^6^ are available in the Gene Expression Omnibus under accession no. GSE151091. Single-cell RNA-sequencing data from Cheng et al.^23^ are available from the European Genome-phenome Archive (EGA) under accession no. EGAS00001002927. Other data supporting the findings of this study are available from the corresponding author on reasonable request. Source data are provided with this paper.

## Code availability

Apps for the cloud-based computing platform, DNAnexus, were written to perform the custom analyses described above, which are available in Zenodo^67–70^.
