# Supplementary material for: Somatic mutations distinguish melanocyte subpopulations in human skin
Source: Nat Cell Biol. 2026 Apr 27;28(6):1300–8. doi: 10.1038/s41556-026-01943-7 (PMC13264844; doi:10.1038/s41556-026-01943-7)
Supplement: Supplementary file 1 — Reporting Summary [file 41556_2026_1943_MOESM1_ESM.pdf]

nature portfolio

Corresponding author(s): DR. A. Hunter Shain

Last updated by author(s): 02/19/2026

Reporting Summary

Nature Portfolio wishes to improve the reproducibility of the work that we publish. This form provides structure for consistency and transparency in reporting. For further information on Nature Portfolio policies, see our Editorial Policies and the Editorial Policy Checklist.

Statistics

For all statistical analyses, confirm that the following items are present in the figure legend, table legend, main text, or Methods section.

n/a	Confirmed	
☐	☒	The exact sample size (*n*) for each experimental group/condition, given as a discrete number and unit of measurement
☐	☒	A statement on whether measurements were taken from distinct samples or whether the same sample was measured repeatedly
☐	☒	The statistical test(s) used AND whether they are one- or two-sided<br>*Only common tests should be described solely by name; describe more complex techniques in the Methods section.*
☐	☒	A description of all covariates tested
☐	☒	A description of any assumptions or corrections, such as tests of normality and adjustment for multiple comparisons
☐	☒	A full description of the statistical parameters including central tendency (e.g. means) or other basic estimates (e.g. regression coefficient) AND variation (e.g. standard deviation) or associated estimates of uncertainty (e.g. confidence intervals)
☐	☒	For null hypothesis testing, the test statistic (e.g. *F*, *t*, *r*) with confidence intervals, effect sizes, degrees of freedom and *P* value noted<br>*Give P values as exact values whenever suitable.*
☒	☐	For Bayesian analysis, information on the choice of priors and Markov chain Monte Carlo settings
☐	☒	For hierarchical and complex designs, identification of the appropriate level for tests and full reporting of outcomes
☐	☒	Estimates of effect sizes (e.g. Cohen's *d*, Pearson's *r*), indicating how they were calculated

Our web collection on statistics for biologists contains articles on many of the points above.

Software and code

Policy information about availability of computer code

Data collection	No softwares or codes were used for data collection.
Data analysis	Code Availability<br><br>Apps for the cloud-based computing platform, DNA nexus, were written to perform the custom analyses described above, which are available here: [https://github.com/ShainLab/Single_Cell_Somatic_Mutation_Caller] and [https://github.com/ShainLab/HaploPrep].

For manuscripts utilizing custom algorithms or software that are central to the research but not yet described in published literature, software must be made available to editors and reviewers. We strongly encourage code deposition in a community repository (e.g. GitHub). See the Nature Portfolio guidelines for submitting code & software for further information.

Data

Policy information about availability of data

All manuscripts must include a data availability statement. This statement should provide the following information, where applicable:

- Accession codes, unique identifiers, or web links for publicly available datasets
- A description of any restrictions on data availability
- For clinical datasets or third party data, please ensure that the statement adheres to our policy

This work is part of the Human Tumor Atlas Network (HTAN), supported by the National Cancer Institute (U01 CA294536). HTAN's mission is to generate

comprehensive atlases of the molecular changes underlying cancer initiation and progression. The datasets generated in this study are described below. These data will also be accessible through the HTAN data portal after the next data release (currently anticipated for Spring of 2026).

Single-cell DNA and RNA sequencing data from human skin that support the findings of this study have been deposited in dbGaP under accession codes phs001979.v1.p1 [https://dbgap.ncbi.nlm.nih.gov/beta/search/?OBJ=study&TERM=phs001979.v1.p1] and phs003683.v2.p1 [https://dbgap.ncbi.nlm.nih.gov/beta/search/?OBJ=study&TERM=phs003683.v2.p1]. These accession numbers provide access to the raw sequencing FASTQ files.

Access to these datasets is restricted because participant consent permits data use only for biomedical research and does not allow unrestricted public release of individual-level genomic information. Investigators can request access through the dbGaP Data Access Committee via the dbGaP portal, and approved users receive data under institutional approvals and data use agreements consistent with the original consent. Requests are typically reviewed within 4–8 weeks, and data remain available for the duration of the repository's retention policy.

Intermediate levels of analysis are also provided. Somatic mutation calls for single cells are included in Supplementary Table 2 and deposited in cBioPortal [https://www.cbioportal.org/study/summary?id=normal_skin_melanocytes_2024]. Xenium spatial transcriptomics datasets from skin tissue sections are available in GEO under accession code GSE286964 [https://www.ncbi.nlm.nih.gov/geo/query/acc.cgi?acc=GSE286964]. Additional processed results are provided in the Supplementary Information. All analyses on publicly available data were performed with appropriate citation of the original source.

Source data are provided with this study. All other data supporting the findings of this study are available from the corresponding author on reasonable request.
All data analyses were conducted using publicly available software packages. The pipeline and tools for somatic mutation calling is available at [https://github.com/ShainLab/Single_Cell_Somatic_Mutation_Caller], a coverage analysis tool for counting the number of bases in the bam file with a specified coverage at [https://github.com/ShainLab/Footprints_v.0.1], script for identifying heterozygous Single Nucleotide Polymorphisms and haplotype phasing at [https://github.com/ShainLab/HaploPrep], the phylogenetic tree construction script at [https://github.com/ShainLab/Phylogenetic_tree]. All codes are publicly available under the MIT License.
For all the tools that are publicly available, detailed description of the softwares/tools used are as follows:

DNA sequencing data were aligned to the hg19 version of the human genome using the BWA-MEM algorithm (v2.0.5). Following alignment, Picard (v4.1.2.0) (https://broadinstitute.github.io/picard/) was used to deduplicate the genomic reads. The aligned reads were then realigned around indels and recalibrated using the Genome Analysis Toolkit (GATK v4.1.2.0). In terms of the RNA samples, sequencing reads were aligned to both the genome and transcriptome using STAR align (v2.1.0). Deduplication of RNA reads was performed using Picard (v4.1.2.0). Read counts for each gene were quantified with RSEM (v1.2.0). Different versions of some of these software programs were tested (https://github.com/ShainLab/Single_Cell_Somatic_Mutation_Caller/tree/main/benchmarking_data) and had little impact on the results.

To identify germline heterozygous SNPs, FreeBayes (v1.3.1) was employed, and the SNPs were filtered to include only those overlapping with known SNPs in the 1000 Genomes Project36 and with allelic frequencies between 40-60%. Among other uses, this information was utilized to detect allelic dropout, assisting in the removal of samples with low coverage or amplification biases. A DNAnexus app is available to perform these operations (https://github.com/ShainLab/HaploPrep/tree/main). We also include benchmarking data comparing FreeBayes to another SNP caller (https://github.com/ShainLab/HaploPrep/tree/main/benchmarking_data/VariantCaller_test).

CNVkit (v0.9.6.2) was used to infer copy number alterations from both DNA and RNA sequencing data A reference was used in both modes. When running CNVkit in DNA mode, each cell was compared to a panel of normals from the same sequencing batch. When running in RNA mode, all other cells from the same sequencing batch were used as a reference. Other CNVkit parameters were set to default. As described in greater detail in the documentation, for DNA copy number inference, CNVkit accepts an interval file of baits and automatically generates "bins" for both on-target and off-target reads. The bin size depends on the bed file of baits. For this study, CNVkit automatically generated bins, resulting in approximately 250bp on-target bins and 20kb off-target bins. For RNA copy number inference, each gene is treated as its own bin. CNVkit use circular binary segmentation (CBS) to segment files by default, producing .cns files of copy number segments. Heatmaps showing copy number inferences are available on figshare for each cell (https://doi.org/10.6084/m9.figshare.28700804.v2). Copy number alterations were rare and therefore not a focus of the present study.

Additionally, a candidate list of short insertions and deletions were generated using Pindel . We elected to use Pindel because it was the choice of PCAWG40. We also benchmarked it against MuTect2, and for calling indels from DNA that had undergone whole genome amplification, we concluded the specificity was superior to MuTect2. The candidate indels were further filtered for a minimum of four reads and variant allelic frequency of 15% or higher. These candidates were visually inspected in integrative genomics viewer to further remove: (a) insertions within homopolymer tracts, which could theoretically be assigned to multiple positions within the tract, and (b) indels occurring adjacent to a germline SNP. These types of false positive indels were previously reported by the Pan-cancer analysis working group. The median number of indels per exome was 2 – a small proportion of the overall mutation burden, an extremely small proportion of mutations in this study.

For identifying somatic point mutations, the initial candidate point mutations were called using MuTect2 (v4.1.2.0). The mutations were further filtered to remove artifacts that arose during whole genome amplification (https://github.com/ShainLab/Single_Cell_Somatic_Mutation_Caller). The app uses phasing and expression information to validate somatic mutations. True mutations were expected to show linkage to these nearby SNPs, while artifacts typically displayed incomplete or no linkage.

For phasing, we begin by using the GATK ReadBackedPhasing function using the patient's normal BAM file, a VCF of germline heterozygous SNPs (stringently filtered for high specificity), and an annotated VCF file as input. This generated a phased VCF file, which was then used as input for our "Single_Cell_Somatic_Mutation_Caller" custom software application: [https://github.com/ShainLab/Single_Cell_Somatic_Mutation_Caller]. Within the application, custom scripts split tumor BAM reads into haplotype-specific BAM files. Candidate somatic mutations were then counted in the bam files corresponding to each haplotype using the samtools mpileup function. These counts were compiled into a summary table, and our app categorizes each variant as either "clonal" (i.e. in complete linkage with the nearest germline SNP), subclonal (i.e. not in linkage), or it makes no call if there are no nearby SNPs and/or coverage is low in the haplotype bam files.

Second, RNA validation was conducted by verifying the presence of somatic mutations in RNA sequencing data, as artifacts were not anticipated to be present in both genomic and transcriptomic datasets. This approach considers only mutations with sufficient RNA-seq coverage and excludes those introducing premature stop codons, which could trigger nonsense-mediated decay. This strategy also does not consider expression of X-chromosome mutations in females because there is a 50% chance the mutation resides on the silenced allele. After counting the number of reference and mutant reads in matching RNA-sequencing data, our app calls each mutation as either "expressed", "not expressed", or it makes no call if coverage is insufficient, the mutation is truncating, and/or the mutation is on the X-chromosome of female sample.

After validating (or invalidating) candidate mutations within the expressed and phase-able portions of the genome, candidate mutations that do not reside in

regions (e.g. variants in genes that are not expressed and far away from germline heterozygous SNPs) were inferred to be mutations or artifacts based on their variant allele frequency (VAF). True mutations exhibited a normal distribution centered around 50%, while artifacts typically had lower allelic frequencies and fewer overall reads. Cutoff thresholds were set using receiver operating characteristic (ROC) curves, trained on known mutations/artifacts from each sample, to maximize both sensitivity and specificity (Fig. S2).

The specificity of mutation calls was benchmarked in several ways, as shown in figure S2. The allele frequencies of mutations were bimodal – most were centered at 50% with a smaller subset centered at 100%. This pattern is exactly what one would expect for heterozygous and homozygous mutations in cells where copy number alterations are exceedingly rare. Artifacts tended to have much lower allele frequencies, consistent with their origins in later rounds of whole genome amplification. Moreover, mutations had expected mutational signatures (aging or UV radiation), whereas artifacts had known artifactual signatures. These patterns held true whether mutations were inferred from their allele frequencies or validated based on their haplotype distribution, gene expression, or presence in other cells.

Once mutations were called, mutation burdens were calculated as mutations per megabase. The total megabases of coverage is denoted as footprint of the genome covered. This is calculated using footprint software11 and included only nucleotide base pairs with a minimum of 10X or greater coverage.

2. Mutation burden and mutational signature analyses
Mutation burdens were calculated as mutations per megabase. We counted the number of mutations as described above. To determine the footprint of genome with sufficient coverage in each clonal expansion, we calculated the footprints (as we described previously in Tang et al. Nature, 2020) to count the precise number of basepairs with 10X coverage or greater. For the analysis of mutational signatures, we compiled somatic mutations across all cells from both cohorts (Table S4) and established trinucleotide contexts for single base substitutions using the Bioconductor library BSgenome.Hsapiens.UCSC.hg19 (v1.4.3). This analysis was restricted to cells harboring a minimum of 10 mutations, as profiles with fewer mutations are statistically unreliable. A custom forward stagewise algorithm using SigProfilerAssignment (v0.1.8) was applied to build a mutational profile based on 78 pre-defined COSMIC (v3.7) signatures previously extracted by SigProfiler 68. The minimum number of SBS mutations for the signature analysis is set at 10. The signatures for all the cells are depicted as stacked barplots in figure 1b (bottom panel) showing the fractions of top 7 signatures. Signatures present in less than 10% of cells were grouped into an [others] category.

Research involving human participants, their data, or biological material

Policy information about studies with human participants or human data. See also policy information about sex, gender (identity/presentation), and sexual orientation and race, ethnicity and racism.

Reporting on sex and gender

For this study, sex and gender was not part of the study design since our aim was to perform multi-omic profiling of epidermal melanocytes from body sites that experience different degrees of habitual sun exposure. Skin was collected from the shoulders, buttocks, trunk, and head/neck area. Skin biopsies were collected from 31 unique donors (23 females and 8 males). The gender of living donors recruited for this study are self-reported. For cadavers, this information was based off medical records. All the details on the exact gender, age and other information on the donors are shared as a supplementary file (table S1). However, samples from both male and female donors were included in all analyses to minimize sex-related biases in the results.

Reporting on race, ethnicity, or other socially relevant groupings

For this study, the only criterion was to analyze and compare epidermal melanocytes across different anatomical site with differing mutation burdens. Therefore, race, ethnicity, age and other socially relevant groupings were not part of the study design. Donors ranged from 29 to 86 years of age. This study include Black, White, Asian and Hispanic donors. For living donors recruited for this study; race, ethnicity and age are self-reported. For cadavers, this information was based off medical records.

Population characteristics

See above.
Skin biopsies were collected from 31 unique donors (23 females and 8 males).
Donors ranged from 29 to 86 years of age. This study include Black, White, Asian and Hispanic donors.

Recruitment

A total of 55 skin biopsies were collected from 31 donors across multiple anatomical sites at the University of California, San Francisco (UCSF) and Northwestern University. At UCSF, biopsies were obtained from donors enrolled in the UCSF Willed Body Program for research purposes. Living patients consented to participating in this study through approved protocols by Institutional Review Board of University of California, San Francisco (22-36678) and Institutional Review Board of Northwestern University (STU00211546).

Cadaver tissues were obtained from donors who had provided broad pre-mortem consent, through their living will, permitting the use of their tissues for medical research and/or educational purposes. Donor demographic information, including age, sex, and gender, was obtained from preapproved Vital Statistics Information Sheets. The living donors provided self-reported information and consent, in accordance with their IRBs, to publish data on their UV radiation exposure and various risk factors to skin cancer including age, sex, ancestry, sun exposure, sunscreen use and tanning bed use, by completing a questionnaire. Comparable information for UCSF Willed Body Program donors was obtained from their consented Vital Statistics Information Sheets.

The biopsies were collected as either punch or shave biopsies, with diameters of 3mm or 5mm. Additionally, tissue samples (buccal mucosa, blood, or skin biopsies from distinct anatomical sites) were also collected to establish the genome for each donor.

Participant compensation was not provided.

Ethics oversight	Consent from all living donors was obtained in accordance with protocols approved by the institutional review boards of the respective universities (UCSF IRB 22-36678 and Northwestern IRB STU00211546). For deceased donors, informed consent was part of their will.

Note that full information on the approval of the study protocol must also be provided in the manuscript.

Field-specific reporting

Please select the one below that is the best fit for your research. If you are not sure, read the appropriate sections before making your selection.

☒ Life sciences ☐ Behavioural & social sciences ☐ Ecological, evolutionary & environmental sciences

For a reference copy of the document with all sections, see nature.com/documents/nr-reporting-summary-flat.pdf

Life sciences study design

All studies must disclose on these points even when the disclosure is negative.

Sample size	In total, we measured somatic mutations from 297 epidermal melanocytes from 31 donors derived from 58 skin biopsies from 31 donors. The sample size for melanocytes were based on all available genomic data. No statistical methods were used to pre-determine sample sizes but our sample sizes are similar or larger than those reported in previous publications (PMID: 41309580, 33029006, 41385634)
Data exclusions	No data was excluded from the analyses.
Replication	To validate the gene expression profiles of HighMut and LowMut melanocytes, we collected additional biopsies (n=3) from the shoulders of three new donors (details in Supplementary Table 1). From these biopsies, 15 melanocytes were sequenced using the same approach as for the main (discovery) cohort. Applying identical cutoff thresholds and selection criteria, melanocytes with high and low mutation burdens were identified (Extended Data Fig. 3a). Enrichment of HighMut- and LowMut-associated genes from the discovery cohort was assessed in the validation cohort by UMAP clustering of differentially expressed genes (Extended Data Fig. 3b) and heatmap comparison (Extended Data Fig. 3c).<br><br>The robustness of the gene signature was further assessed using repeated 5-fold cross-validation, with 10 repetitions. In each training fold (80% of the cells) of every cross-validation split, we performed differential gene expression analysis with DESeq2, and identified the significant genes, using the same settings and significance threshold (Benjamini-Hochberg false discovery rate adjusted p-value < 0.1) applied to derive the final gene signature. To reduce variability due to a single cross-validation split, the entire 5-fold cross-validation procedure was repeated 10 times, resulting in 50 separate differential expression analyses (10 repetitions × 5 folds). We then plotted the percentage of training folds in which each gene was deemed significant. 300 out of 9989 genes were significant in at least one training fold. The 42 genes that we defined as HighMut and LowMut genes, based on a one time differential gene expression analysis of all cells, were all among the top 48 genes most frequently discovered across cross-validation training folds (Extended Data Fig. 4).<br><br>Key findings regarding the subpopulations of high- and low-mutation burden melanocytes were observed across multiple donors and anatomic sites. Differential expression signatures between the two melanocyte subpopulations observed were reproduced in an independent validation cohort from three additional donors (13 samples total), not included in the original analyses. These were independently supported by spatial transcriptomics (10X Genomics Xenium analyzer) in separate FFPE specimens from 3 different donors.
Randomization	This was an observational study of human skin biopsies; random allocation to experimental groups was not applicable as molecular comparisons focus on epidermal melanocytes from the same body sites having varying levels of mutational burden.
Blinding	Similar to randomization, blinding is not applicable to this study. The study's results are empirical, and the researchers have no influence on the outcome.

Reporting for specific materials, systems and methods

We require information from authors about some types of materials, experimental systems and methods used in many studies. Here, indicate whether each material, system or method listed is relevant to your study. If you are not sure if a list item applies to your research, read the appropriate section before selecting a response.

Materials & experimental systems

n/a	Involved in the study	
☒	☐	Antibodies
☒	☐	Eukaryotic cell lines
☒	☐	Palaeontology and archaeology
☒	☐	Animals and other organisms
☒	☐	Clinical data
☒	☐	Dual use research of concern
☒	☐	Plants

Methods

n/a	Involved in the study	
☒	☐	ChIP-seq
☒	☐	Flow cytometry
☒	☐	MRI-based neuroimaging

Plants

Seed stocks	NA
Novel plant genotypes	NA
Authentication	NA
