# Supplementary material for: Somatic mutations distinguish melanocyte subpopulations in human skin
Source: Nat Cell Biol. 2026 Apr 27;28(6):1300–8. doi: 10.1038/s41556-026-01943-7 (PMC13264844; doi:10.1038/s41556-026-01943-7)
Supplement: Supplementary file 2 — Peer Review File [file 41556_2026_1943_MOESM2_ESM.pdf]

nature portfolio

Peer Review File

Somatic mutations distinguish melanocyte subpopulations in human skin

Corresponding Author: Dr Hunter Shain

Version 0:

Decision Letter:

Dear Hunter,

Your manuscript "Somatic mutations distinguish melanocyte subpopulations in human skin", has now been seen by 3 referees, who are experts in skin, cancer, mutations, DNAseq, RNAseq (referee 1); mutations, melanocytes (referee 2); and melanocytes, melanoma, DNAseq (referee 3), and whose comments are pasted below. In light of their advice, we regret that we cannot offer to publish the study in Nature Cell Biology.

As you will see, although reviewers #2 and #3 find this work potentially interesting, they still raise serious concerns that question the strength of the dataset, whereas reviewer #1 raises very critical points also regarding the strength of the data and of the novel conclusions that can be drawn at this stage.

Although we cannot publish your paper, it may be appropriate for another journal in the Nature Portfolio. If you wish to explore the journals and transfer your manuscript please use our manuscript transfer portal. You will not have to re-supply manuscript metadata and files, unless you wish to make modifications. For more information, please see our manuscript transfer FAQ page.

We are very sorry that we could not be more positive on this occasion, but we thank you for the opportunity to consider this work. Please, let me know if you would like me to consult with our colleagues at Communications Biology to see if they would be interested in inviting a revision based on these reviewer reports.

With kind regards,
Stelios

Stylianos Lefkopoulos, PhD
He/him/his
Senior Editor, Nature Cell Biology
Springer Nature
Heidelberger Platz 3, 14197 Berlin, Germany

E-mail: stylianos.lefkopoulos@springernature.com
Twitter: @s_lefkopoulos
LinkedIn: linkedin.com/in/stylianos-lefkopoulos-81b007a0

Reviewers' comments:

Reviewer #1 (Remarks to the Author):

This manuscript builds on the author's previous publication on somatic mutations in melanocytes, approximately doubling the number of single cells analysed. The mutation burden and phenotype of cells is analysed by DNA and RNA sequencing. The study aims to show that melanocytes with a high and low mutational burden have distinct phenotypes and localization in the skin.

The total number of cells reported here is nevertheless modest (297). The cells derive from 31 donors, so 10 cells per donor

are sampled. The strategy of expanding cells in vitro and then deriving colonies from single cells may alter cell phenotypes and select for cells that can adapt to growth in culture.

Fundamental to the analysis is the splitting of cells into high and low burden based on quartile. Many body sites are sampled, some sun exposed others not. If there are only 3-4 cells at many of the sites is separating the cells into quartiles valid? From Fig 1 it looks as if there are only 15 or so cells in the low and low burden groups for samples with a skin burden of 8 SNV/Mb or more. How do the findings change if the quartile cut off is adjusted? Does the bulk of the cells lying in the 25-75% range have intermediate properties between high and low groups?

The spatial analysis is interesting but relies on a single donor. This should be extended for a 'cell atlas'.

There are significant issues with the quality and openness of bioinformatic analyses performed in this paper. Strategies for filtering and variant calling currently appear 'cherry-picked' and details insufficient to re-produce the analyses performed. The requisite QC plots needed to validate filtering strategies and algorithm choices are not provided so it is difficult to have confidence in the results presented without the reader running significant additional re-analyses. Furthermore it is not possible to reproduce the results here without accompanying code for each analysis and filtering criteria.

Most tools used are 6 years (or more) out of date, e.g. - GATK 4.1.2.0, freebayes (v1.3.1) are used and were released in 2019. The version of Picard tools mentioned also does not exist, the latest release on the page cited in methods is 3.3.0, so it is assumed used an older version bundled into GATK was used.

The variant calling strategy amalgamates several programs, it is unclear and not explained why the recommended pipeline for a single program was not followed from start to finish. GATK local indel re-alignment is mentioned but germline variants are not called with haplotype caller. It is unclear why this route has been pursued and a comparison of freebayes vs haplotype caller would be useful to validate the choice. Also, the choice of then using a third calling program for INDELs i.e. PINDEL is unusual. Could all these calls not come from haplotype caller for germline and mutect2 to keep a standard and uniform pipeline strategy consistent with current standards?

Why was manual curation employed when it was stated that INDEL realignment was already performed by GATK? How reproducible and what are the criteria for identifying artifacts? No code is provided to reproduce any of the variant calling pipelines and the steps to reproduce the analyses performed in this paper. Are there some data quality issues that are not discussed, and without further explanation this is hard to justify.

The strategy of filtering heterozygous variants is concerning, heterozygous mutations are only considered here between 40-60%. 50% obviously is the theoretical allele frequency for a heterozygous mutation, however to reach this value the sequencing depth needs to be sufficiently high. This may be the case for some of the target panel samples which range from 53 to 3093, but it is unclear if these numbers are before or after removing duplicate reads. The maximum coverage for exomes is 175. While the few samples of several 1000x coverage may be sufficient for the calls to have equilibrated around 50% most samples probably have not.
Variant callers employ genotype likelihood values and model the most likely genotype and so the range of heterozygous variants most callers will return is 20-80% allele fractions. So by narrowing the thresholds in an apparently arbitrary way, the data may be biased. There is no discussion or reporting of how many variants were removed per sample. There is no plot of the VAF distributions in the cohorts of sequencing and discussion of how many variants were filtered then by these thresholds. The methods section also does not discriminate as to whether the deep panel was treated in the same ways. This filtering strategy is likely to be poor for the depth reported of the exome samples.

The modelling of genotype quality score should ensure high quality calls, if GATK had been used end to end Variant Quality Score Recalibration could have been used also to supplement GQ filtering in a method that is published and more sensitive and not likely biasing the data used.

For known SNP filtering could a larger dataset such as gnomAD be utilised instead of 1KG? Basic details missing from QC such as what the low coverage or amplification biases criteria to remove samples were, further hindering reproducibility.

Was additional haplotype phasing done in addition to that of mutect2? It is unclear based on the methods provided when linkage is discussed, details and linkage thresholds are needed and a statement of how many SNPs were then excluded.

Why is the data manually filtered between the RNA and DNA? Could the BAM files for RNA and DNA from the same individual not be supplied into mutect2 and allow the variant caller all the information to make the calling much more accurate and reproducible? This feature was available from version 4.1. This would allow the variant caller to fully leverage the power of all the sequencing data in a much more sophisticated and nuanced approach to improve the results

With the RNA-seq, the version of RSEM v1.2.0 used is over 12 years old. Why has such an old version been utilised? The latest release version is 1.3.3, released 4 years ago. Details are lacking for the versions of gene transcripts that were used, i.e. refseq, ensemble or gencode and again versions should be state. Reproducing the data on such old software may not be possible.

Basic details of the copy number calling are missing for CNVkit. For example What was the bin size used? What log thresholds and values were applied to call changes? A 5 year old version of CNVkit is still being used here. Was any cohort analysis employed in any of the processing steps of CNVkit? Which segementation algorithm was used?

The single cell parameters used in Xenium analysis are not provided either for the XOA pipeline or Seurat. It is unclear as to the batch correction, doublet identification mitochondrial percentage checking and basic qc for single cell analysis. Also no details of the Deseq2 for DE-analysis are provided. There are also large gaps in the description of the single cell analysis methodology.

These technical and reproducibility concerns cast doubt on the reproducibility of data analysis and must be addressed before publication.

Reviewer #2 (Remarks to the Author):

This manuscript reports a considerable amount of work, representing an in-depth follow up to the same group's publication in 2020 (Tang et al) and some very interesting conclusions. Their previous work, in essence, found that melanocytes in sun exposed regions of skin carried a much higher mutation load than those from regions not exposed to sunlight.

The approach used allows for a more comprehensive analysis of single melanocytes than single cell RNA sequencing. Single isolated melanocytes are grown on to provide several hundred cells which can be subsequently analysed by DNA and RNA sequencing and morphological analysis. However. as the authors acknowledge, the cells are removed from their environment for numerous cell generations and there is the possibility of changes occurring. Nevertheless a coherent story emerges suggesting this is not an issue.

The authors use a strategy to identify "outlier" melanocytes in skin biopsies with high mutation burden among cells with low burden and vice versa. They suggest low burden cells have been protected from mutation either biologically or physically. What do the authors think about the high mutation cells, some of which appear to have a burden 6 times higher than their neighbouring cells? Are they much older cells that have been in the epidermis longer?

Supp Table 1 shows the number of successful clones grown for each biopsy. They set a minimum mutation burden of 3 (per Mb?) to be included in analysis. Did they set a lower limit of number of melanocytes analysed per biopsy so there is confidence in the median mutation burden estimate? Most of the donors are past 60 years of age, but some are younger. Is there any correlation between mutation burden and age, or incidence of low burden cells and age (older people might be expected to have few follicular melanocytes).

They carry out differential gene expression between high and low burden melanocytes. Based on transcript analysis the authors suggest low burden melanocytes are more stem-like or neural crest (progenitor like). I would have expected follicle-located (and thus mutation protected) pigment cells to fall into two categories: melanocytes actively synthesising melanin and melanocyte stem cells located in the bulge region. Can they distinguish two populations in the low burden transcript data? Published data suggests they would be DCT positive but KIT, TYRP1 and TYR negative or low. (Of course, it is possible that the bulge stem cells are not identified, or one of the populations is lost somehow)

Comparing gene expression in the two populations with existing datasets they find a significant overlap between high-burden cells and "adult melanocytes" and low burden cells with "melanocyte stem cells". The P value for the overlaps are very significant, albeit the actual number of transcripts overlapping is small.

Further evidence as to the "stem like" characteristic comes from cell morphology analysis and their finding that high burden cells are more dendritic, low burden cells more ovoid, fitting observations by others that bulge localized stem cells are more ovoid.

Finally using spatial transcriptomics and transcript signatures that identify their two melanocyte classes they show most low burden melanocytes are either in the hair follicle or in the epidermis close to follicles. The morphology of the skin sections is not great, and it is hard to know if the low burden cells they are identifying in the follicle are located in the bulge. They seem to be distributed throughout. I would have expected some differentiated cells to be localized at the follicle base. Where are the few high-burden signature cells in the follicle localized? Along those lines are there any low burden (UV protected) cells with a high burden signature which might correspond to differentiated follicular melanocytes? (see in this context my comment above about two populations in the follicles)

A minor point; they find HERC2 to be preferentially expressed in high mutation burden melanocytes. They comment (page 4) that HERC2 is well established as genetically associated with pigmentation. This is true, but it is believed that the genetic association is via an intronic SNP that is regulating the expression of adjacent OCA2.

Reviewer #3 (Remarks to the Author):

The study aimed at characterizing subpopulations of melanocytes using clonal expansion and multi-Omics analysis, identifying distinct high- and low-mutation melanocytes, in human skin tissue. Individual melanocytes from donors were harvested, clonally expanded, and subjected to DNA sequencing (exome sequencing) and RNA-Seq. An additional skin

samples was further imaged using a workflow involving Xenium to localize melanocytes with low- and high-mutation burden, based on a derived gene-expression signature.

General comment: The study's aims are of interest as many aspects of melanoma biology are yet to be explored using new (single) cell technologies. The approach is robust (like clonal expansion and sequencing) and the bioinformatics methods are sound. The use of the Xenium methodology to identify the mutational status of melanocytes via their gene expression signature is innovative.

There are some major drawbacks:

1. The threshold defining high- and low-mutation melanocytes is not well justified (Figure 1). The authors observed a difference in mutation burden across melanocytes between participants and decided to implement a relative, rather than absolute mutation level threshold. The relative threshold defines high-mutation melanocytes as those in the upper quantile vis a vis melanocytes from the same participant. That way, the number of observed mutations defining a high-mutation melanocyte differs from participant to participant. An absolute threshold would apply the same mutation level to any melanocyte. There is no biological justification for this approach, particularly as the authors claim that melanocytes exit the stem cell niche after accumulating a certain number of mutations. It is unclear why this number would differ from participant to participant, or from body site to body site. Did the authors try to define an absolute threshold. Did the analysis and conclusion hold?

2. There is no validation of the gene expression signature derived from low- and high-mutation melanocytes. This signature is crucial for the Xenium experiment, but its robustness is not demonstrated. The authors should perform a cross-validation experiment where some melanocytes are not included in the training and derivation of the signature. During testing, hold-out melanocytes would be used to test the validity of the signature, i.e. whether the signature correctly identifies whether the hold-out melanocytes are of low- or high-mutational nature.

3. The Xenium experiment is conducted in a single participant only. The uncertain robustness of the signature (see #2) might necessitate to use of more participants (unless the signature is close to 100% correct).

4. It is unclear why the authors decided to perform exome rather than whole genome sequencing, which would have allowed for the derivation of a more robust mutational estimate (i.e. more genome real estate and chance to observe mutations). Was it because DNA from only 230 cells (per colony) were used? Why did the authors limit colony growth to that number?

**For Nature Portfolio general information and news for authors, see http://npg.nature.com/authors.

Version 1:

Decision Letter:

26th January 2026

Dear Hunter,

Thank you for submitting your revised manuscript "Somatic mutations distinguish melanocyte subpopulations in human skin" (NCB-A57094A-Z). As you know, it was seen a few days ago by the original referees (their comments are below). As you also know, because reviewer #3 continued to raise some concerns, we decided to discuss these concerns with reviewer #1 (except for reviewer #3's comment about WGS, which we decided we would not need you to address with more data for further consideration at Nature Cell Biology). Reviewer #1 agreed with the rest of the points raised by reviewer #3 (points #1 and #2) and we then reached out to you to ask for your response to them; eventually, with your permission, we shared your response to these comments via e-mail with reviewer #3. You can find the input reviewer #3 offered on this response in their report, section 'ADDITIONAL COMMENTS ON THE AUTHORS' RESPONSE TO MY POINTS ABOVE'.

Taking everything into consideration, we have decided that we'll be happy in principle to publish your manuscript in Nature Cell Biology as a Resource, pending minor revisions to satisfy the referees' final requests (the remaining point by reviewer #1 and the remaining point that reviewer #3 raises in the part of their report 'ADDITIONAL COMMENTS ON THE AUTHORS' RESPONSE TO MY POINTS ABOVE') and to comply with our editorial and formatting guidelines.

If the current version of your manuscript is in a PDF format, please email us a copy of the file in an editable format (Microsoft Word or LaTex)-- we cannot proceed with PDFs at this stage.

We are now performing detailed checks on your paper and will send you a checklist detailing our editorial and formatting

requirements in about 7-10 days. Please do not upload the final materials and make any revisions until you receive this additional information from us.

Thank you again for your interest in Nature Cell Biology. Please do not hesitate to contact me if you have any questions.

Best wishes,
Stelios

Dr. Stylianos Lefkopoulos
He/him/his
Senior Editor, Nature Cell Biology
Springer Nature
Heidelberger Platz 3, 14197 Berlin, Germany

E-mail: stylianos.lefkopoulos@springernature.com
Bluesky: @slefkopoulos.bsky.social
LinkedIn: www.linkedin.com/in/stylianos-lefkopoulos-81b007a0

Reviewer #1 (Remarks to the Author):

The authors have thoroughly revised and substantially improved the manuscript. The key point about the cut off between low and high mutated melanocytes has been well addressed, as have concerns about the spatial analysis. The informatics is now much better explained and the choices made have been justified. Overall, this study adds to our knowledge of human (as opposed to mouse) skin biology and will be of interest in the fields of epidermal biology and carcinogenesis. Minor point: "melanocytes in the hair follicle occupy a privileged niche, protected from UV radiation, but periodically migrate out of the hair follicle to replenish the interfollicular epidermis after waves of photodamage'…This is a possible but unproven explanation. Most photodamage is low doses of UV, which may not trigger such migration. Equally other epidermal injuries may result in migration. Perhaps move this speculation from abstract to discussion?

Additional comments on the remaining points by reviewer #3
Reviewer #3 highlights that issues remain about the definition and validation of high- and low-mutation melanocytes. This is a critical issue for the paper and the comment '…there is a definite need to show numerically the validity / robustness of the proposed list of differentially expressed genes' should be addressed. The additional experiment provided for validation is not convincing as 'not high-mutation validation melanocytes do not convincingly fall within the range of the discovery melanocytes'. As suggested a computational approach may help confirm the robustness of the definition of high- and low-mutation cells.

Reviewer #1 (Remarks on code availability):

Code is now accessible and is much better explained.

Reviewer #2 (Remarks to the Author):

This revised manuscript deals with the concerns I had with the initial submission. I believe there are interesting and important conclusions drawn. In particular the identification of putative stem cells or melanocyte progenitors in the epidermis. Previous studies have shown migration of such progenitors from hair follicle to epidermis in melanocyte-depleted mouse skin and in vitiligo skin but the work described here shows progenitors present in non-pathological samples.

Reviewer #2 (Remarks on Protocol(s)):

Concerns of other reviewers are around bioinformatic methods. This is not my expertise.

Reviewer #3 (Remarks to the Author):

Summary: The revision addresses the reviewer‘s concerns to some degree (more Xenium experiments, experiments aimed at validating the gene expression signature of high- and low-mutation melanocytes). However, there are still some questions with regard the definition of high- and low-mutation melanocytes; and the validation experiments do not provide a numerical assessment of the validity of the gene expression signature. As the whole study hinges on the validity of that signature, I don‘t believe the study is ready for publication quite yet. The authors should find a way to numerically validate the signature - see comment 2. Given such an assessment, the question of the correct definition (comment 1) becomes less relevant.

Comment 1. The first question referred to the definition of high- and low-mutation melanocytes, and the resulting set of differentially expressed genes. The authors provide additional data (supplementary figure S3). The authors show that -

irrespective of the exact definition (i.e. fixed threshold vs quantiles) -the set of differentially expressed genes between high- and low-mutation melanocytes overlap considerably. However, the number of non-overlapping genes is also high, particularly for the fixed mutation definition (last column, n=742 non overlapping genes). Table S3 thus does NOT settle the question whether the authors used the correct definition (i.e. quantiles). The authors claim to have chosen an approach / definition for „ensuring … reproducible gene expression profiles". As discussed (Comment 2) below, this has only been partially demonstrated.

Comment 2. The second question is a most crucial one. The authors were asked to valide the list of differentially expressed genes via cross-validation. The authors refrained from doing so, arguing that the list has already been defined (i.e. all data has been used to define the list). While I see this point, there is a definite need to show numerically the validity / robustness of the proposed list of differentially expressed genes. (A) An alternative to cross-validation is bootstrapping, which could show - for example - how often (% of bootstrap samples) a particular gene is among the list of differentially expressed genes. Bootstrapping would not affect the already established list of genes. (B) The authors provide another alternative - validation via a newly set of sequenced melanocytes. Unfortunately, data from only 6 out of 13 newly sequenced melanocytes were useful for further analysis (due to the strict „quantile" definition of high- and low-mutation melanocytes; all melanocytes could have been used in the „threshold" definition). The authors do not perform a differential expression analysis on these 6 samples (why? - this would have been, even if suboptimal, a way to show numerically the robustness of the signature) and provide, instead, a heatmap and UMAP of the expression profiles of the six melanocytes. The heatmap seems to show an overall TREND that the validation melanocytes show similar profiles than the discovery set. The UMAP shows good concordance with the low-mutation validation melanocytes, but the high-mutation validation melanocytes do not convincingly fall within the range of the discovery melanocytes. In summary, the authors do not provided a numerical assessment of the robustness of the gene expression signature, but a „visual" - only partially convincing - assessment instead (heatmap/UMAP).

Comment 3: I congratulate the authors for adding additional Xenium experiments.

Comment 4: While I see the argument of overall costs, WGS costs have come down considerably and I don't see the need for deep sequencing in cell lines of clonally expanded melanocytes, i.e. it should be possible to detect somatic mutations even at low sequencing depth.

ADDITIONAL COMMENTS ON THE AUTHORS' RESPONSE TO MY POINTS ABOVE
I can definitely accept the arguments brought forward regarding point #1.

I am glad that the authors provide a numerical assessment of the robustness of the signature (point #2). This strengthens the paper a lot. I do not have access to the exact methods - but the results look convincing, i.e. the chosen gene list seems very robust. A little remark: The author use a threshold of $p_{adj}<0.2$ and $p_{adj}<0.1$; these thresholds are typically used for the FDR (not p-value); so I am not quite sure whether the notation is correct.

Version 2:

Decision Letter:

Dear Hunter,

I am pleased to inform you that your manuscript, "Somatic mutations distinguish melanocyte subpopulations in human skin", has now been accepted for publication in Nature Cell Biology. Congratulations!

Thank you for sending us the final manuscript files to be processed for print and online production, and for returning the manuscript checklists and other forms. Your manuscript will now be passed to our production team who will be in contact with you if there are any questions with the production quality of supplied figures and text.

Over the next few weeks, your paper will be copyedited to ensure that it conforms to Nature Cell Biology style. Once your paper is typeset, you will receive an email with a link to choose the appropriate publishing options for your paper and our Author Services team will be in touch regarding any additional information that may be required.

After the grant of rights is completed, you will receive a link to your electronic proof via email with a request to make any corrections within 48 hours. If, when you receive your proof, you cannot meet this deadline, please inform us at rjsproduction@springernature.com immediately.

You will not receive your proofs until the publishing agreement has been received through our system.

Due to the importance of these deadlines, we ask that you please let us know now whether you will be difficult to contact over the next month. If this is the case, we ask you provide us with the contact information (email, phone and fax) of someone who will be able to check the proofs on your behalf, and who will be available to address any last-minute problems.

If you have any questions about our publishing options, costs, Open Access requirements, or our legal forms, please contact ASJournals@springernature.com

Once your paper has been scheduled for online publication, the Nature press office will be in touch to confirm the details. An online order form for reprints of your paper is available at https://www.nature.com/reprints/author-reprints.html. All co-authors, authors' institutions and authors' funding agencies can order reprints using the form appropriate to their geographical region.

Publication is conditional on the manuscript not being published elsewhere and on there being no announcement of this work to any media outlet until the online publication date in Nature Cell Biology.

You may wish to make your media relations office aware of your accepted publication, in case they consider it appropriate to organize some internal or external publicity. Once your paper has been scheduled you will receive an email confirming the publication details. This is normally 3-4 working days in advance of publication. If you need additional notice of the date and time of publication, please let the production team know when you receive the proof of your article to ensure there is sufficient time to coordinate. Further information on our embargo policies can be found here: https://www.nature.com/authors/policies/embargo.html

Please note that *Nature Cell Biology* is a Transformative Journal (TJ). Authors may publish their research with us through the traditional subscription access route or make their paper immediately open access through payment of an article-processing charge (APC). Authors will not be required to make a final decision about access to their article until it has been accepted. Find out more about Transformative Journals

Authors may need to take specific actions to achieve compliance with funder and institutional open access mandates. If your research is supported by a funder that requires immediate open access (e.g. according to Plan S principles or the NIH public access policy) then you should select the gold OA route, and we will direct you to the compliant route where possible. Because authors warrant under our subscription licensing terms that they haven't committed to licensing any version of their article under a licence inconsistent with the terms of our agreement – including the applicable embargo period – publication under the subscription model isn't suitable for authors whose funders require no embargo.

To assist our authors in disseminating their research to the broader community, our SharedIt initiative provides you with a unique shareable link that will allow anyone (with or without a subscription) to read the published article. Recipients of the link with a subscription will also be able to download and print the PDF.

If your paper includes color figures, please be aware that in order to help cover some of the additional cost of four-color reproduction, Nature Portfolio charges our authors a fee for the printing of their color figures. Please contact our offices for exact pricing and details.

As soon as your article is published, you will receive an automated email with your shareable link.

If you have not already done so, we strongly recommend that you upload the step-by-step protocols used in this manuscript to protocols.io (https://protocols.io), an open online resource that allows researchers to share their detailed experimental know-how. All uploaded protocols are made freely available and are assigned DOIs for ease of citation. Protocols and Nature Portfolio journal papers in which they are used can be linked to one another, and this link is clearly and prominently visible in the online versions of both. Authors who performed the specific experiments can act as primary authors for the Protocol as they will be best placed to share the methodology details, but the Corresponding Author of the present research paper should be included as one of the authors. By uploading your Protocols onto protocols.io, you are enabling researchers to more readily reproduce or adapt the methodology you use, as well as increasing the visibility of your protocols and papers. You can also establish a dedicated workspace to collect your lab Protocols. Further information can be found at https://www.protocols.io/help/publish-articles.

You can use a single sign-on for all your accounts, view the status of all your manuscript submissions and reviews, access usage statistics for your published articles and download a record of your refereeing activity for the Nature Portfolio.

Nature Cell Biology encourages authors presenting evidence for cell, biological, molecular, and genetic interactions to consider communicating these findings using Biofactoid (https://biofactoid.org/). This tool helps users share a searchable representation of interactions (e.g. binding, gene expression, post-translational modification) between genes, gene products, or chemicals. Information added to Biofactoid, with author attribution, is shared on social media and public databases, such as Pathway Commons, where it can be discovered and analyzed in the context of a large and growing corpus of knowledge.

Please feel free to contact us if you have any questions.

With kind regards,
Stelios

Dr. Stylianos Lefkopoulos
He/him/his

Senior Editor, Nature Cell Biology
Springer Nature
Heidelberger Platz 3, 14197 Berlin, Germany

E-mail: stylianos.lefkopoulos@springernature.com
Bluesky: @slefkopoulos.bsky.social
LinkedIn: www.linkedin.com/in/stylianos-lefkopoulos-81b007a0

Click here if you would like to recommend Nature Cell Biology to your librarian
http://www.nature.com/subscriptions/recommend.html#forms

** Visit the Springer Nature Editorial and Publishing website at www.springernature.com/editorial-and-publishing-jobs for more information about our career opportunities. If you have any questions please click here.**

Open Access This Peer Review File is licensed under a Creative Commons Attribution 4.0 International License, which permits use, sharing, adaptation, distribution and reproduction in any medium or format, as long as you give appropriate credit to the original author(s) and the source, provide a link to the Creative Commons license, and indicate if changes were made.
In cases where reviewers are anonymous, credit should be given to 'Anonymous Referee' and the source.
The images or other third party material in this Peer Review File are included in the article's Creative Commons license, unless indicated otherwise in a credit line to the material. If material is not included in the article's Creative Commons license and your intended use is not permitted by statutory regulation or exceeds the permitted use, you will need to obtain permission directly from the copyright holder.
To view a copy of this license, visit https://creativecommons.org/licenses/by/4.0/

Reviewer #1 (Remarks to the Author):

This manuscript builds on the author's previous publication on somatic mutations in melanocytes, approximately doubling the number of single cells analysed. The mutation burden and phenotype of cells is analysed by DNA and RNA sequencing. The study aims to show that melanocytes with a high and low mutational burden have distinct phenotypes and localization in the skin.

The total number of cells reported here is nevertheless modest (297).

The size of the study is simultaneously large and small. It is the largest study (one of the only studies) to describe the somatic mutational landscapes of melanocytes, and it is on par with other prominent papers that clonally expand cells of normal tissues to measure their mutational landscapes[1–3]. We believe that it is a major resource to the community to have matched exome sequencing data and whole transcriptome sequencing data from hundreds of colonies of individual cells.

On the other hand, the reviewer is correct that a sample size in the hundreds has limitations, depending on the questions one seeks to ask. However, we believe that the main conclusion from this study (that there are mutationally distinct subpopulations of melanocytes in human skin) is strongly supported by our sample size.

Altogether, thank you for the comment. We discuss this limitation in lines 102-106.

The cells derive from 31 donors, so 10 cells per donor are sampled. The strategy of expanding cells in vitro and then deriving colonies from single cells may alter cell phenotypes and select for cells that can adapt to growth in culture.

It is true that clonal expansion may introduce biases, but the fact that we observe mutationally distinct subpopulations of cells shows that our approach is able to capture cells in these states. Surely, there is interesting biology that our approach cannot capture, but this does not take away from the insights that we are capable of uncovering.

Nonetheless, the reviewer raises an important point, and we discuss this limitation in lines 96-100 of the manuscript.

Fundamental to the analysis is the splitting of cells into high and low burden based on quartile. Many body sites are sampled, some sun exposed others not. If there are only 3-4 cells at many of the sites is separating the cells into quartiles valid? From Fig 1 it looks as if there are only 15 or so cells in the low and low burden groups for samples with a skin burden of 8 SNV/Mb or more. How do the findings change if the quartile cut off is adjusted? Does the bulk of the cells lying in the 25-75% range have intermediate properties between high and low groups?

We agree that the way in which we split cells into high and low mutation burden groups was a fundamental decision to our analysis, and the other reviewers asked about this, too. There came a point in our study where we had to "freeze" the definition so that we could finalize the list of differentially expressed genes between high- and low- mut melanocytes and proceed with appropriate probes for Xenium analyses. Hence, we put a great deal of thought into the definition of high- and low- mut melanocytes. In this revision, we show in figure S3 some of the alternative definitions considered. As the reviewer will see, the differentially expressed genes were remarkably stable

across the various conditions. We also have text (lines 116-124, 492-495) in the manuscript explaining why we eventually settled upon the chosen definition over the alternatives.

The reviewer asks an interesting question regarding the properties of cells in the middle 50% of mutation burdens. In this revision, we include a new heatmap showing the gene expression structure of high- and low- mut cells as well as the cells in between (Fig. S4). For the reviewer's convenience, the key UMAPs are also shared here as reviewer figure 1. From this data, we conclude that the intermediate cells are a mixture of high- and low- mutation burden cells (mostly high) rather than in an "in-between" state.

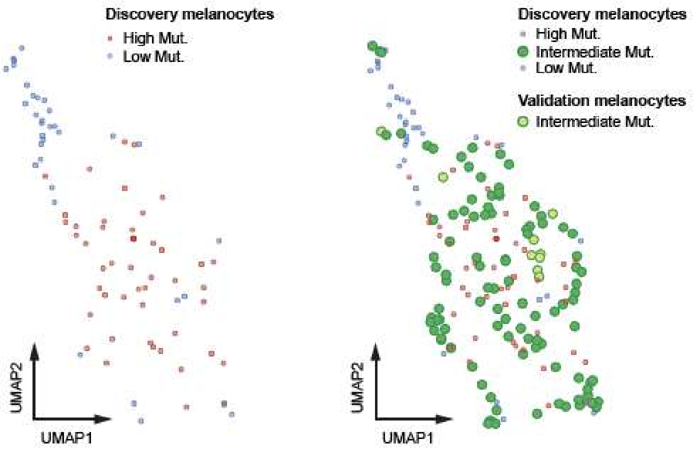

Reviewer Figure 1. Melanocytes with intermediate mutation burdens can cluster with either high- or low- mutation burden cells. A UMAP was generated from differentially expressed genes between melanocytes with high and low mutation burdens. As expected, melanocytes with high (red) or low (blue) mutation burdens tend to cluster apart (left UMAP). Melanocytes with intermediate mutation burdens were overlaid in the UMAP on the right and cluster with both low or high mutation burden melanocytes.

The spatial analysis is interesting but relies on a single donor. This should be extended for a 'cell atlas'.

We agree with the reviewer, and this point was echoed by the other reviewers as well. In this revision, we have added Xenium data from 2 more donors (now totaling 3 donors). This new data significantly strengthened the main conclusions of our manuscript. Melanocytes with HighMut and LowMut gene expression programs were also present in the new skin samples, and their spatial distribution was nearly identical to the original Xenium data from a single donor. Specifically, the LowMut melanocytes were enriched in hair follicles, though they could be found in the interfollicular epidermis, particularly near the infundibulum (i.e. the "openings") of the hair shaft. Meanwhile, the HighMut melanocytes were enriched in the interfollicular epidermis. We have added and updated figures 5, S8 and S9 with the new data (line number 217-221, 604-607 and 700-732).

There are significant issues with the quality and openness of bioinformatic analyses performed in this paper. Strategies for filtering and variant calling currently appear 'cherry-picked' and details insufficient to re-produce the analyses performed. The requisite QC plots needed to validate filtering strategies and algorithm choices are not provided so it is difficult to have confidence in the results presented without the reader running significant additional re-analyses.

In this revision, we have added quality control data supporting the accuracy of our mutation calls. Figure S2 is a new figure that aggregates mutational data from all cells in the study. We have also added similar plots for every individual cell in this study into fig share: https://figshare.com/articles/dataset/309_individual_melanocytes_genotyped_from_human_skin/28700804?file=53514620 (since there are hundreds of cells, it would be onerous to include this data as a supplemental figure). For the reviewer's convenience, we will walk through, here, one plot for one cell as well as the aggregate data.

In reviewer figure 2A (first column), we show a set of potential somatic variants, stratified by their variant allele frequencies from a clonal expansion of one cell. These variants were detected by MuTect2 or Pindel. They are a mixture of somatic mutations and amplification artifacts. We were able to validate or invalidate a subset of somatic mutations (shown in the second and third columns of reviewer figure 2A) using three strategies, described below. For the remaining variants (shown in the fourth column of reviewer figure 2A), we infer their mutational status based on their allele frequencies, as discussed below.

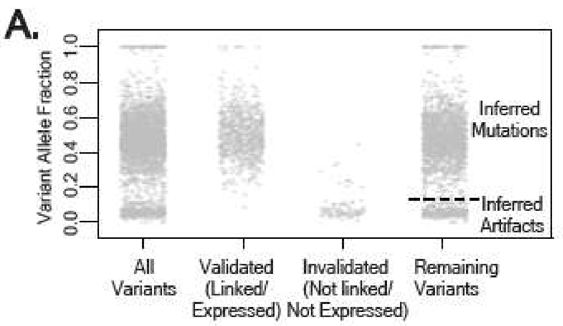

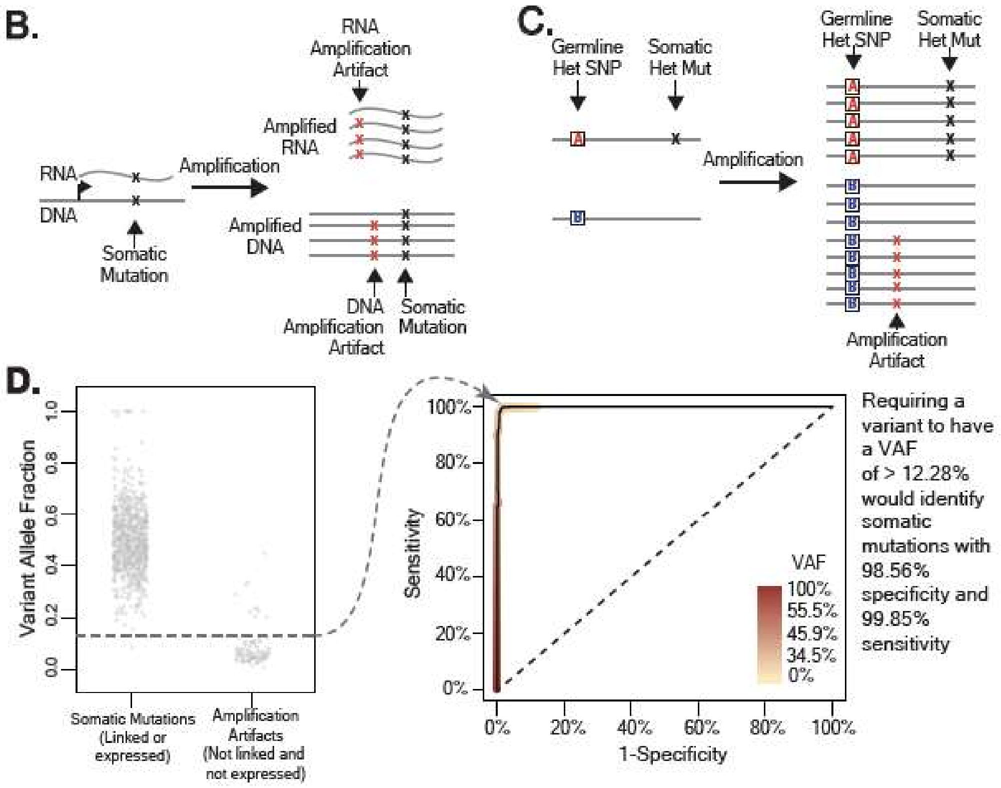

Reviewer Figure 2. Patterns to distinguish true mutations from amplification artifacts. A. Potential somatic variants (individual data points) were called with MuTect and Pindel in a clonal expanion of melanocytes as compared to patient-matched normal tissue. They are stratified, here, by their variant allele frequencies. Some variants could be validated or invalidated as somatic mutations (see panels B and C). The remaining variants were inferred to be mutations or artifacts based on their allele frequencies (see panel D). **B.** Mutations in expressed genes are evident in both DNA- and RNA- sequencing data, whereas amplification artifacts are not. **C.** Germline polymorphisms, distinguished here as "A" and "B" alleles, are in linkage with somatic mutations but not amplification artifacts. **D.** Establishing a variant allele fraction (VAF) cut-off to infer somatic mutations within a clone. The left panel depicts the VAFs for known somatic mutations and known amplification artifacts from a single clone. The right panel depicts a ROC curve, showing the VAF at which sensitivity and specificity of somatic mutation calls would be maximized when inferring the mutational status of variants based on VAF alone. Variants that fell within expressed or phase-able portions of the genome were classified as mutations or artifacts (see methods). The remaining variants were inferred based on the VAF cut-off, which maximized sensitivity and specificity of somatic mutation calls.

First, we consider a somatic mutation validated if it was detected in DNA-sequencing data and also present in RNA-sequencing data (reviewer figure 2B). The variant would be invalidated (i.e. an artifact) if it was detected in DNA-sequencing data but not in RNA-sequencing data (assuming a minimal level of coverage in the RNA-sequencing data and that the mutation does not result in a premature stop codon, which could lead to nonsense-mediated decay).

Second, we[4] and others[5] independently discovered that somatic mutations occur in complete linkage with nearby SNPs, whereas amplification/sequencing artifacts do not (reviewer figure 1C). We use these linkage patterns to further distinguish mutations from artifacts in our data.

Third, some cells are clonally related to other cells from the same patient, indicated by the fact that they share a similar set of somatic alterations. When a variant was found in 2 or more cells from the same patient, we considered it to be a true somatic mutation. However, in the example case, shown in reviewer figure 2, the melanocyte was not related to any cells.

Overall, variants were validated as somatic mutations if they were expressed, linked, or found in 2 or more cells from the same patient. Variants were invalidated if they were not detected in RNA-sequencing data of a highly expressed gene or not in linkage with nearby polymorphisms.

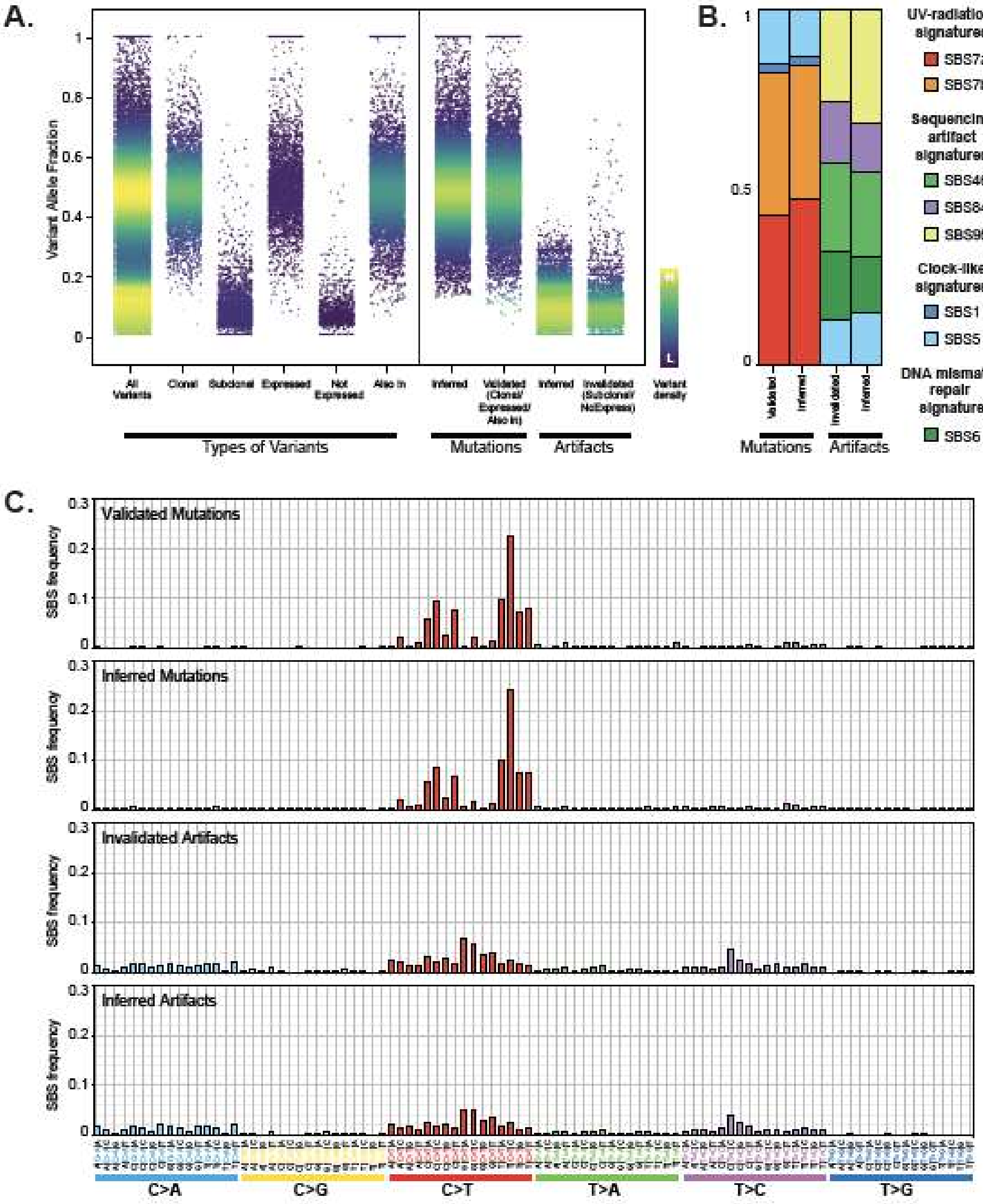

Reviewer Figure 3. Distinguishing true mutations from sequencing and amplification artifacts. A. The scatterplot shows the variant allele fractions (VAFs) of all variants identified in melanocytes from this study. Somatic mutations are first validated within the expressed and phasable regions of the genome. The remaining variants are inferred based on their VAFs (see Methods). **B.** The stacked plot illustrates the mutational signatures of validated mutations, inferred mutations, invalidated artifacts, and inferred artifacts. **C.** The 96-bar plots show the frequency of the six possible somatic point mutations across all trinucleotide contexts. The panels, from top to bottom, correspond to validated mutations, inferred mutations, invalidated artifacts, and inferred artifacts.

For variants that could not be validated or invalidated with one of the above approaches, we used a mutant allele frequency cutoff (reviewer figure 2D). For each sample, we stratified the allele frequencies of variants with a known status (i.e. validated or invalidated, reviewer figure 2D left plot),

and we calculated what the sensitivity and specificity of mutation calls would have been at different allele frequency cutoffs (reviewer figure 2D, right plot). Next, we select an allele frequency cutoff that would maximize the sensitivity and specificity of mutation calls on this known data, and we apply it to the variants whose status is not known.

There are several reasons to believe that our mutations are real, as shown in reviewer figure 3 (this reviewer figure is now a supplemental figure in the manuscript, Fig. S2). Somatic mutations, whether they were validated or inferred, have allele frequencies centered at 50% (or 100%), which would be expected for a heterozygous mutation (or homo/hemi-zygous mutation) (reviewer figure 3A). Moreover, their mutational signatures are comprised of mutational processes expected to operate on skin – namely, aging (SBS1 and 5) and UV radiation (SBS7a/b) (reviewer figure 3B). Artifacts, whether they were invalidated or inferred, have relatively low allele frequencies, and their mutational signatures include known artifactual signatures (SBS 46, 84, and 95) (reviewer figure 3B). When looking at the 96 bar plots, validated and inferred mutations occur in nearly identical tri-nucleotide contexts (reviewer figure 3C). (line number 517-525)

Furthermore it is not possible to reproduce the results here without accompanying code for each analysis and filtering criteria.

Thank you for the comment. We have created a new GitHub page that includes more code and descriptions of operations than was previously accessible.

https://github.com/ShainLab/Single_Cell_Somatic_Mutation_Caller
https://github.com/ShainLab/HaploPrep

We begin by describing how we generated sequencing alignments and preliminary variant calls with established software (e.g. BWA, GATK, MuTect2, Pindel, and CNVkit). These steps are relatively common, and we detail what we did, but other labs could substitute different software and/or versions of software without much impact on the final somatic mutation calls. We discuss the impact of versions and differing software in response to your other comments below.

The most innovative bioinformatic component of our single-cell genotyping workflow is the way in which we integrate RNA-sequencing data and haplotype information to distinguish mutations from artifacts. We wrote an app, which is publicly available on the cloud-based platform, DNA nexus, to perform these steps, and the source code is available in the GitHub links above. This is reflected in code availability section (line number 745 to 765).

Most tools used are 6 years (or more) out of date, e.g. - GATK 4.1.2.0, freebayes (v1.3.1) are used and were released in 2019. The version of Picard tools mentioned also does not exist, the latest release on the page cited in methods is 3.3.0, so it is assumed used an older version bundled into GATK was used. The variant calling strategy amalgamates several programs, it is unclear and not explained why the recommended pipeline for a single program was not followed from start to finish.

Our workflow can broadly be divided into three steps: 1. wet lab work to amplify genomic material from individual cells while limiting the introduction of artifacts, 2. bioinformatic work to come up with an initial set of candidate mutations, and 3. bioinformatic work to come up with a final list of somatic mutations, tailored to root out artifacts in samples that have undergone whole genome amplification. For this project, we spent most of our time optimizing steps 1 and 3. The majority of the issues raised by the reviewer pertain to step 2, calling an initial set of candidate variants. We individually address each point below, but in the big picture, this is not a major source of artifacts because any artifacts left

over after this step were eventually removed in step 3 using the haplotyping, RNA validation, and/or MAF filters described above.

For step 2, we use standard software, developed by researchers who work with bulk-cell sequencing data from tumors. The variant calls from bulk-cell tumor pipelines are a helpful starting point, but this software was not designed to call somatic mutations in DNA that has undergone whole genome amplification. Therefore, the goal of step 2 is to come up with a sensitive list of candidate variants, which can eventually be whittled down into a specific list of variants with appropriate filters in step 3. In light of the reviewer’s comments, it was useful to revisit some of these specific software programs and versions of software used in step 2, but the suggestions had little effect on the final mutation calls, largely because our own scripts (in step 3) removed the majority of artifacts anyway.

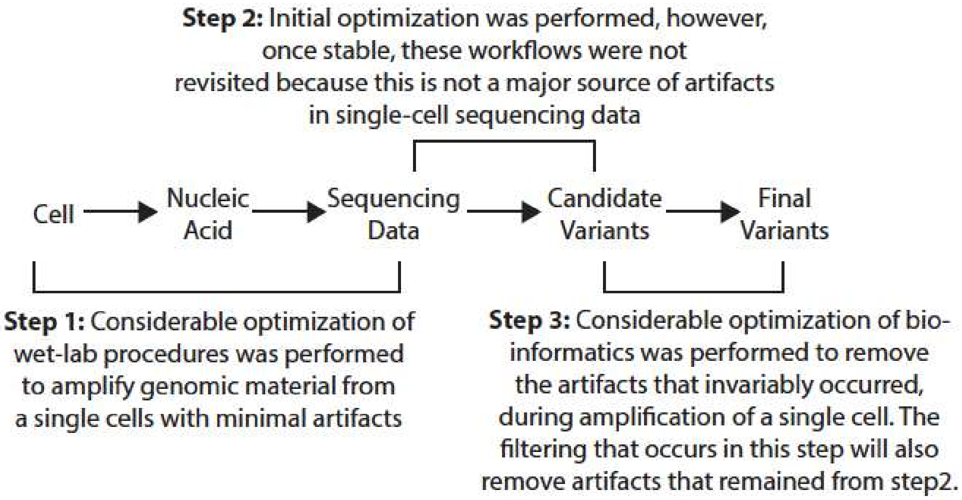

As a practical note, we have steadily accumulated mutational data from individual skin cells over the past 7 years and have written (or use developer-written) cloud-based apps on DNAnexus. When an app is working for us, we generally do not update it.

To begin to illustrate our point, while addressing the reviewer’s specific comments on the version of GATK, we substituted GATK version 4.5.0.0 in place of GATK 4.2.0.0 on one representative cell while keeping everything else the same. These results are shown in more detail in our new Github page, as evidence that either software version can be substituted in step 2 above. The bottom line: substituting GATK versions did not result in a single difference in the final mutation calls. To be sure, there were differences in the initial set of candidates, but these were all low allele frequency variants that were filtered out in later stages of our workflow.

	Total Candidate Somatic Variants	Unique Candidate Somatic Variants	Total Candidate Indels	Unique Candidate Indels	Real Somatic Variants	Real Indels	**Final Mutation Count**	**Coverage (MB)**	**Mut/MB**
GATK4.2	333	121	4	0	59	2	**61**	**43.3**	**1.41**
GATK4.5	235	23	4	0	59	2	**61**	**43.3**	**1.41**

GATK local indel re-alignment is mentioned but germline variants are not called with haplotype caller. It is unclear why this route has been pursued and a comparison of freebayes vs haplotype caller would be useful to validate the choice.

Thank you for this comment. To begin, we wish to point out that the Pan Cancer Analysis Working Group (PCAWG) used a consensus between FreeBayes and Haplotype Caller to identify germline SNPs[6]. They extensively benchmarked FreeBayes, HaplotypeCaller, as well as other software, and they concluded that FreeBayes produced high quality SNPs – in some ways better than HaplotypeCaller.

To further address this point, we also performed a comparison between FreeBayes and GATK HaplotypeCaller on a representative sample. As was the case when we compared GATK versions, there were differences in the initial set of variants, directly output by each software, without any filtering. However, once we applied the exact same filters, as described in the manuscript, to the initial set of SNP calls, the final calls were 98% overlapping, which is in line with the PCAWG benchmarking data[6].

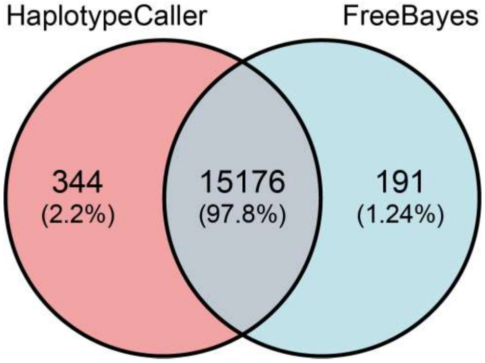

FreeBayes: 15,384 germline heterozygous SNPs
HaplotypeCaller: 15,520 germline heterozygous SNPs
Overlapping variants: 15,176 SNPs (97.8% concordance)
Variants unique to FreeBayes: 191 SNPs (<1.24% of total variants)
Variants unique to HaplotypeCaller: 344 SNPs (<2.2% of total variants)

We inspected several of the SNPs detected only by FreeBayes, and they seem real. We also inspected several of the SNPs called only by HaplotypeCaller, and they also seemed real. In our opinion, either software is viable, and one could potentially increase the sensitivity of germline SNPs detected by taking the union of the data sets.

However, the goal of our study was to detect somatic mutations, and while we use germline heterozygous SNPs to help do this, germline variation is not a focus of our study. Taking the union of Haplotype Caller and FreeBayes results could augment the number of SNPs per individual by 1-2%, but this would have no meaningful effect on the conclusions in this study (a topic we discuss in more detail in response to the reviewer’s questions about filtering germline SNPs below). (line number 388-399)

We have updated our GitHub page to show these comparisons as evidence that the user can use alternative approaches to coming up with an initial candidate list of germline SNPs.

Also, the choice of then using a third calling program for INDELs i.e. PINDEL is unusual. Could all these calls not come from haplotype caller for germline and mutect2 to keep a standard and uniform pipeline strategy consistent with current standards?

We appreciate the reviewer's question regarding our use of Pindel for indel detection. While we understand the preference for pipeline uniformity, we found that Pindel is a superior somatic indel caller for our workflow. The decision to use Pindel is squarely within current standards, as this was the software of choice by the Pan Cancer Analysis Working Group (PCAWG) after an extensive evaluation of multiple tools, including MuTect2[6].

We performed a comparison between Pindel and Mutect2 on a representative sample. They both produced a large number of initial candidates, almost all of which were low allele frequency artifacts. However, after applying the same filters used in our paper, Pindel produced 2 candidate indels, while Mutect2 produced 1,418 total candidate indels.

We show, below, the two indels discovered by Pindel and the top 12 indels discovered by MuTect2, ranked by their allele frequencies. There was a clear pattern whereby MuTect2 was calling indels in homopolymers.

Pindel:

Hugo_Symbol	Chromosome	Start_Position	End_Position	Variant_Classification	Variant_Type	Reference_Allele	Tumor_Seq_Allele2	Genome_Change	Normal_Ref	Normal_Mut	Normal_MAF	Ref	Mut	MAF
NAA11	4	80246473	80246473	Frame_Shift_Del	DEL	C	-	g.chr4:80246473d	187	0	0	74	77	0.510
CHL1	3	403493	403495	Splice_Site	DEL	GGT	-	g.chr3:403493_40	135	0	0	57	44	0.436

Mutect2 (top 12):

Hugo_Symbol	Chromosome	Start_Position	End_Position	Variant_Classification	Variant_Type	Reference_Allele	Tumor_Seq_Allele2	Genome_Change	Normal_Ref	Normal_Mut	Normal_MAF	Sample_Ref	Sample_Mut	MAF
NAA11	4	80246473	80246473	Frame_Shift_Del	DEL	C	-	g.chr4:80246473d	187	0	0	74	77	0.510
CHL1	3	403492	403494	Splice_Site	DEL	TGG	-	g.chr3:403492_40	136	0	0	57	44	0.436
LRRC56	11	551227	551228	Frame_Shift_Ins	INS	-	C	g.chr11:551227_5	153	0	0	14	5	0.263
LRFN3	19	36435928	36435929	3'UTR	INS	-	CC	g.chr19:36435928	127	0	0	36	12	0.250
CHRD	3	184100958	184100959	Intron	INS	-	G	g.chr3:184100958	116	0	0	32	10	0.238
CWC27	5	64163765	64163766	Intron	INS	-	G	g.chr5:64163765_	133	0	0	86	24	0.218
SLC4A3	2	220494110	220494111	Frame_Shift_Ins	INS	-	C	g.chr2:220494110	176	0	0	36	10	0.217
HMX3	10	124895626	124895627	Frame_Shift_Ins	INS	-	C	g.chr10:12489562	65	0	0	22	6	0.214
FTCD	21	47572804	47572805	Intron	INS	-	G	g.chr21:47572804	82	0	0	34	9	0.209
SLC23A2	20	4850568	4850569	Frame_Shift_Ins	INS	-	G	g.chr20:4850568_	82	0	0	47	12	0.203
CASKIN2	17	73498972	73498973	Frame_Shift_Ins	INS	-	G	g.chr17:73498972	141	0	0	37	9	0.196
ELAVL3	19	11577604	11577605	Frame_Shift_Ins	INS	-	C	g.chr19:11577604	109	0	0	30	7	0.189

Example of an indel called by Mutect2 in a homopolymer (polyC) region:

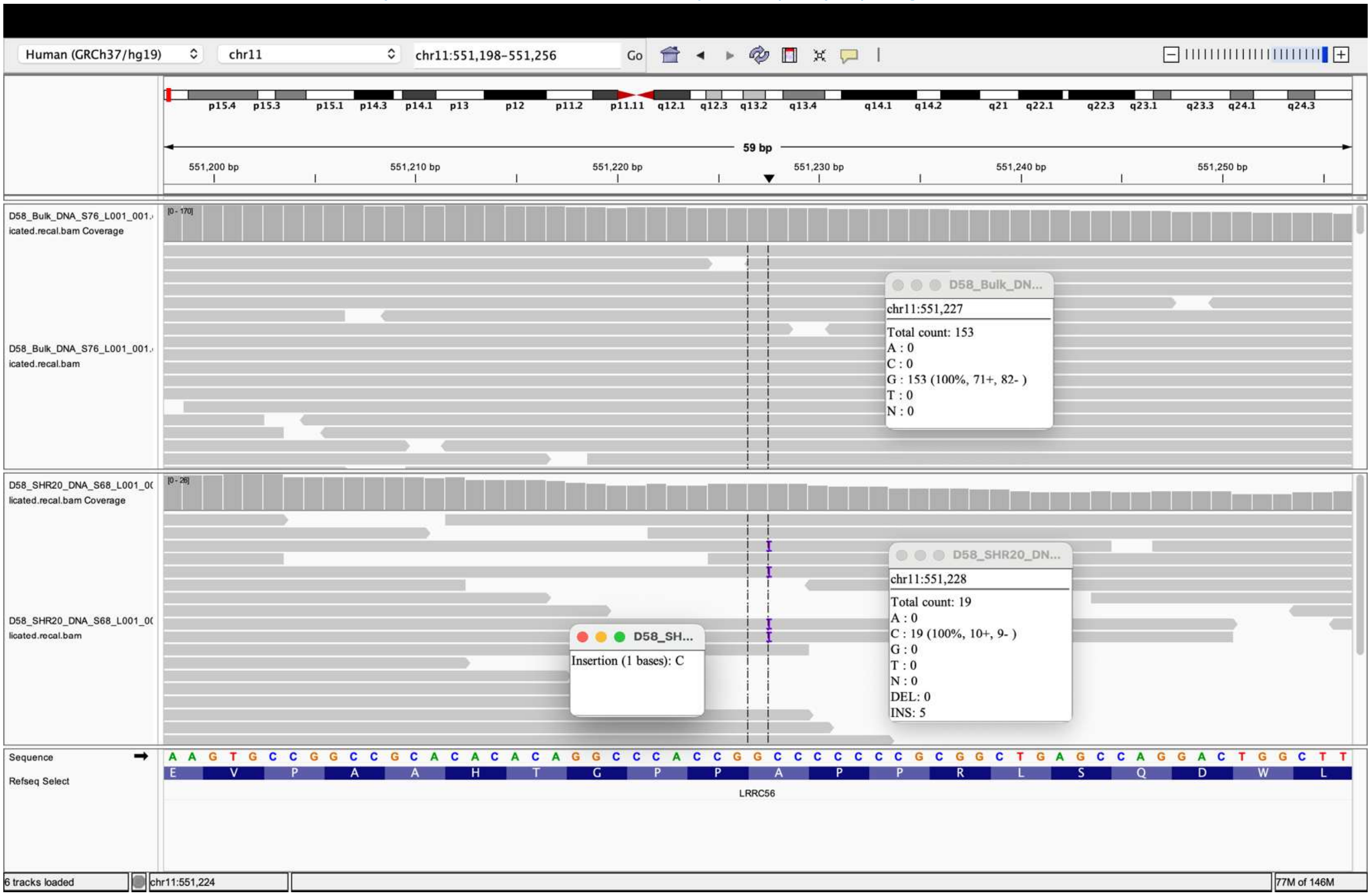

It was encouraging to see both callers identified the same 2 genuine somatic indels, demonstrating equivalent sensitivity. However, Pindel's vastly superior specificity significantly reduced downstream analytical burden and potential for false discoveries. It is important to note that the polymerases used in whole genome amplification are prone to slippage in homopolymer tracts, and this might be why MuTect2, which is more susceptible than Pindel to these types of artifacts, performed sub optimally on this dataset.

Why was manual curation employed when it was stated that INDEL realignment was already performed by GATK? How reproducible and what are the criteria for identifying artifacts? No code is provided to reproduce any of the variant calling pipelines and the steps to reproduce the analyses

performed in this paper. Are there some data quality issues that are not discussed, and without further explanation this is hard to justify.

Manual curation is employed because occasionally there are edge cases where indels are called in regions where alignment is difficult. The PCAWG manuscript has an detailed supplemental methods section[6], where they openly admit, in several circumstances, to manually inspecting certain types of variants. We do not believe that human curation of data is, in and of itself, a disqualifier, but we agree with the reviewer's concern that the curation needs to be described in greater detail.

Indels removed via curation fell into two categories. The most common was an insertion in a homopolymer tract, which could theoretically be assigned to any position within the tract. The second scenario was when indels occurred near germline a SNP. As we read deeper into the PCAWG paper[6], they noted the exact same sources of artifacts in their indels and elected to remove any indel within a homopolymer of 6 bases as well as an indel within 25 base pairs of a germline SNP. In this revision, we describe in greater detail the types of indels that were removed via curation (line number 415-425).

In addition to PCAWG, other prominent studies have acknowledged manual review of their indels. In a recent TRACERX paper, published in Nature (PMID 37046096), Swanton and colleagues noted, "Occasionally, when attempting to identify indels across multiple tumor regions, discrepancies in the start position, end position or length of the indel were identified. In such cases, the longest predicted indel and the maximum sequence related values were reported".

Below, we show an example of an indel that was originally called somatic, but after manually inspecting, we realized that this was a false positive C insertion in a homopolymer tract. In this example, the allele frequency of the insertion was high enough to pass all filters, but it was lower than it should have been (it should be 50% for a bona fide mutation). It was also suspicious that the length of the insertion could vary from read to read. We feel confident in our assessment that this is a false positive, and it would have been difficult to flag this edge case with any strict filter.

Finally, it is important to note that there was an extremely small proportion of indels where the call was changed after manual inspection. We looked at our last 100 cells, and we only found 14 indels, total, that we removed after manual inspection. Inclusion of these indels would have no effect on any conclusions in our manuscript, but we feel that exclusion of them slightly improves the specificity of mutations we report.

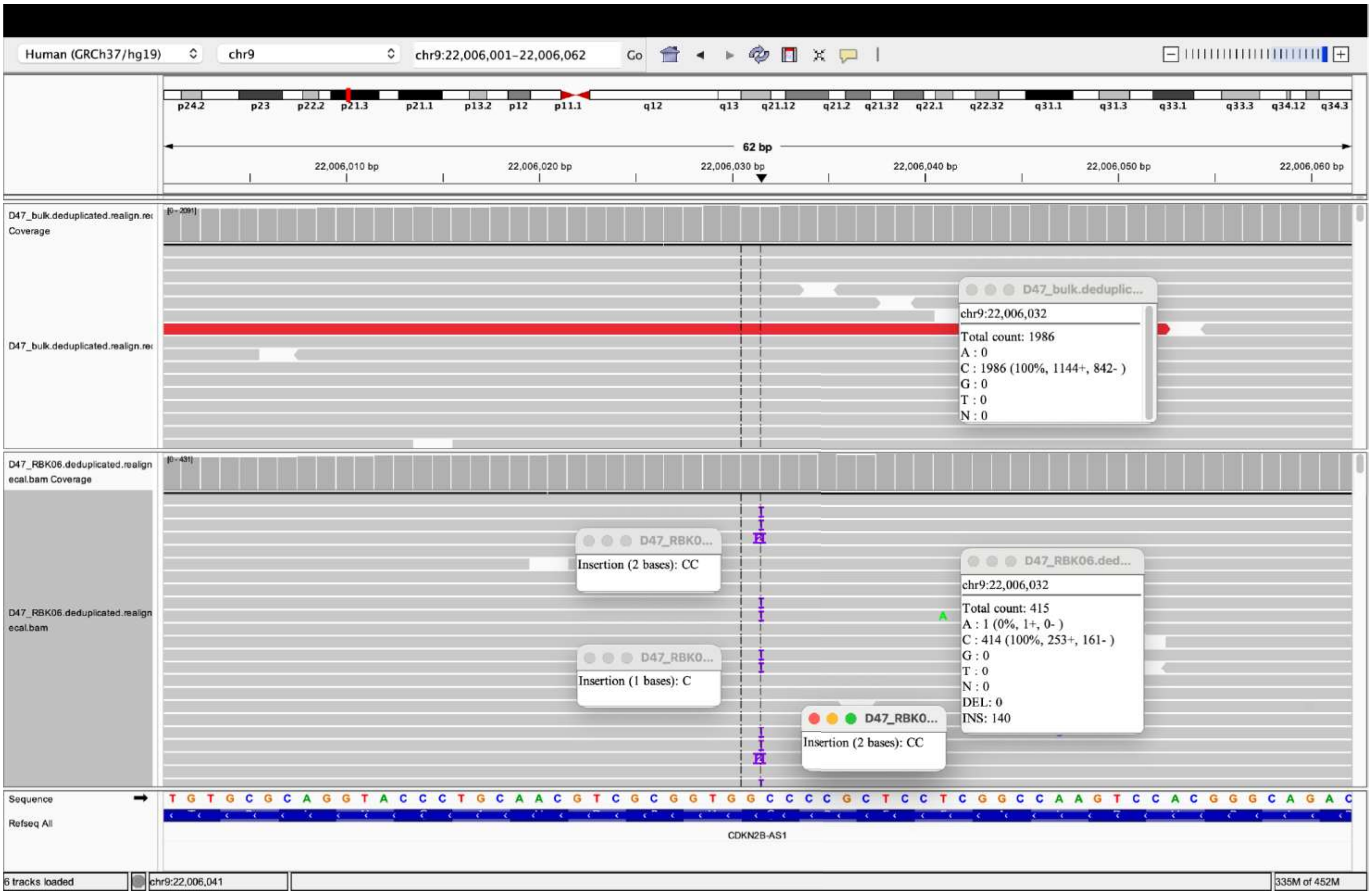

The strategy of filtering heterozygous variants is concerning, heterozygous mutations are only considered here between 40-60%. 50% obviously is the theoretical allele frequency for a heterozygous mutation, however to reach this value the sequencing depth needs to be sufficiently high. This may be the case for some of the target panel samples which range from 53 to 3093, but it is unclear if these numbers are before or after removing duplicate reads. The maximum coverage for exomes is 175. While the few samples of several 1000x coverage may be sufficient for the calls to have equilibrated around 50% most samples probably have not.

Variant callers employ genotype likelihood values and model the most likely genotype and so the range of heterozygous variants most callers will return is 20-80% allele fractions. So by narrowing the thresholds in an apparently arbitrary way, the data may be biased. There is no discussion or reporting of how many variants were removed per sample. There is no plot of the VAF distributions in the cohorts of sequencing and discussion of how many variants were filtered then by these thresholds. The methods section also does not discriminate as to whether the deep panel was treated in the same ways. This filtering strategy is likely to be poor for the depth reported of the exome samples.

The modelling of genotype quality score should ensure high quality calls, if GATK had been used end to end Variant Quality Score Recalibration could have been used also to supplement GQ filtering in a method that is published and more sensitive and not likely biasing the data used.

For known SNP filtering could a larger dataset such as gnomAD be utilised instead of 1KG? Basic details missing from QC such as what the low coverage or amplification biases criteria to remove samples were, further hindering reproducibility.

The reviewer raises excellent points, but it is important to consider why we are calling germline heterozygous SNPs.

Heterozygous SNPs were used for phasing of candidate mutations, which was helpful in validating or invalidating the candidates as somatic mutations. For this goal, specificity of a germline heterozygous SNP is much more important than sensitivity. An artifactual germline SNP could result in the invalidation of any somatic mutation in its vicinity. Perhaps we missed some germline heterozygous SNPs with our strict filters (e.g. by requiring germline SNPs to have narrow allele frequencies around 50%), thereby depriving ourselves of opportunities to validate or invalidate somatic mutations in linkage with the germline SNP; however, these somatic mutation candidates could, instead, be inferred based on their somatic allele frequencies, as described above.

Taken together, the reviewer raises fair points, but for the goals of this manuscript, we could afford the miss some germline heterozygous SNPs with broader allele frequencies and/or rare population frequencies. In return, the high specificity of our SNP calls improved our linkage analyses.

Was additional haplotype phasing done in addition to that of mutect2? It is unclear based on the methods provided when linkage is discussed, details and linkage thresholds are needed and a statement of how many SNPs were then excluded.

We have added new details to the methods and uploaded our HaploPrep app (that is run on DNAnexus) to our GitHub page. The details of the app can be found here: https://github.com/ShainLab/HaploPrep. Briefly, we used the GATK ReadBackedPhasing function. We input the patient's normal bam, a vcf file of germline heterozygous SNPs (pre-filtered as discussed above so that the list has high specificity) and a text file of the annotations for each variant. The output is a phased vcf file.

The phased vcf file is used as an input for our "Single_Cell_Somatic_Mutation_Caller" app on DNAnexus. Within the app, we have custom scripts that will split the reads in a tumor's bam file into haplotype bams, as shown below. Finally, we use the samtools mpileup function to count the number of mutant and reference reads in each haplotype bam file for candidate somatic mutations and report these counts in a table. Below is an example of 3 heterozygous SNPs in linkage with a somatic mutation nearby. (line number 434-444)

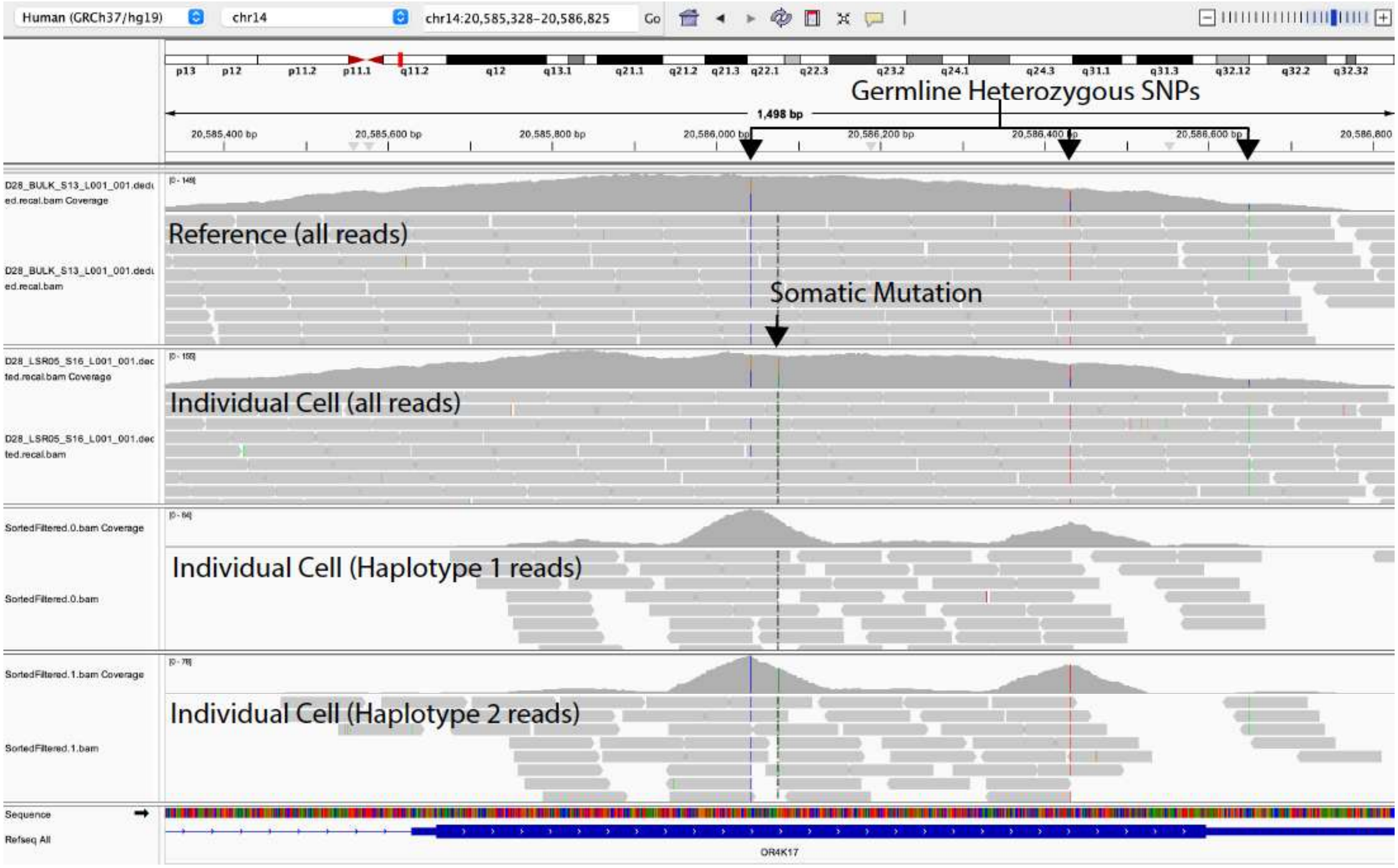

Why is the data manually filtered between the RNA and DNA? Could the BAM files for RNA and DNA from the same individual not be supplied into mutect2 and allow the variant caller all the information to make the calling much more accurate and reproducible? This feature was available from version 4.1. This would allow the variant caller to fully leverage the power of all the sequencing data in a much more sophisticated and nuanced approach to improve the results

Candidate mutations were called in DNA-sequencing data, and then we used the samtools mpileup function to count the number of mutant and ref reads in the RNA bam file from that same sample. This is done within the “Single_Cell_Somatic_Mutation_Caller” app. We do not call mutations directly in RNA-sequencing data, nor do we aggregate the RNA- and DNA- sequencing data from the same cell and attempt to call somatic mutations from the combined data. These are ideas that we had attempted; however, after spending a considerable amount of time attempting to call somatic mutations in RNA-sequencing data using RNA-MuTect, we were not satisfied with the specificity of mutation calls. Therefore, we instead elected to use the RNA-sequencing data solely to validate mutations that were called in DNA-sequencing data.

We do use objective criteria to call a mutation “validated”, “invalidated”, or [in rare cases] “manual inspect” based on the level of support in the RNA-sequencing data. Manual inspect calls occur when there is a low percentage of mutant reads in the RNA-sequencing data. For example, if a variant has 2000-fold coverage in RNA-sequencing data, it would not be unexpected to have a handful of variant reads corresponding to alternate alleles. In such an example, we would not feel comfortable automatically calling the mutation expressed, nor would we feel comfortable automatically declaring that it has no support in RNA-sequencing data. After manual inspection, we sometimes elect not to make a call in either direction. In practice, these manual reviews constitute less than one tenth of a percent of variants – they could be completely disregarded without having any effect on the conclusions of this study. (line number 448-455)

With the RNA-seq, the version of RSEM v1.2.0 used is over 12 years old. Why has such an old version been utilised? The latest release version is 1.3.3, released 4 years ago. Details are lacking for the versions of gene transcripts that were used, i.e. refseq, ensemble or gencode and again versions should be state. Reproducing the data on such old software may not be possible.

RSEM is fairly stable software and has not experienced any update in 4 years, though the reviewer is correct that the version we use is 12 years old. We used a cloud-based app on DNAnexus that was written by the inventors of RSEM. In reviewing the updates that have occurred since version 1.2.0, they appear to be quality of life updates, addressing niche bugs that other users had experienced in the past. None of these bugs have affected us, and we cannot see any updates that would be expected to change the results of this manuscript. Nevertheless, as a test, we ran RSEM v1.2.0 and v1.3.3 (the latest version) on one sample, and the output was nearly identical as shown below:

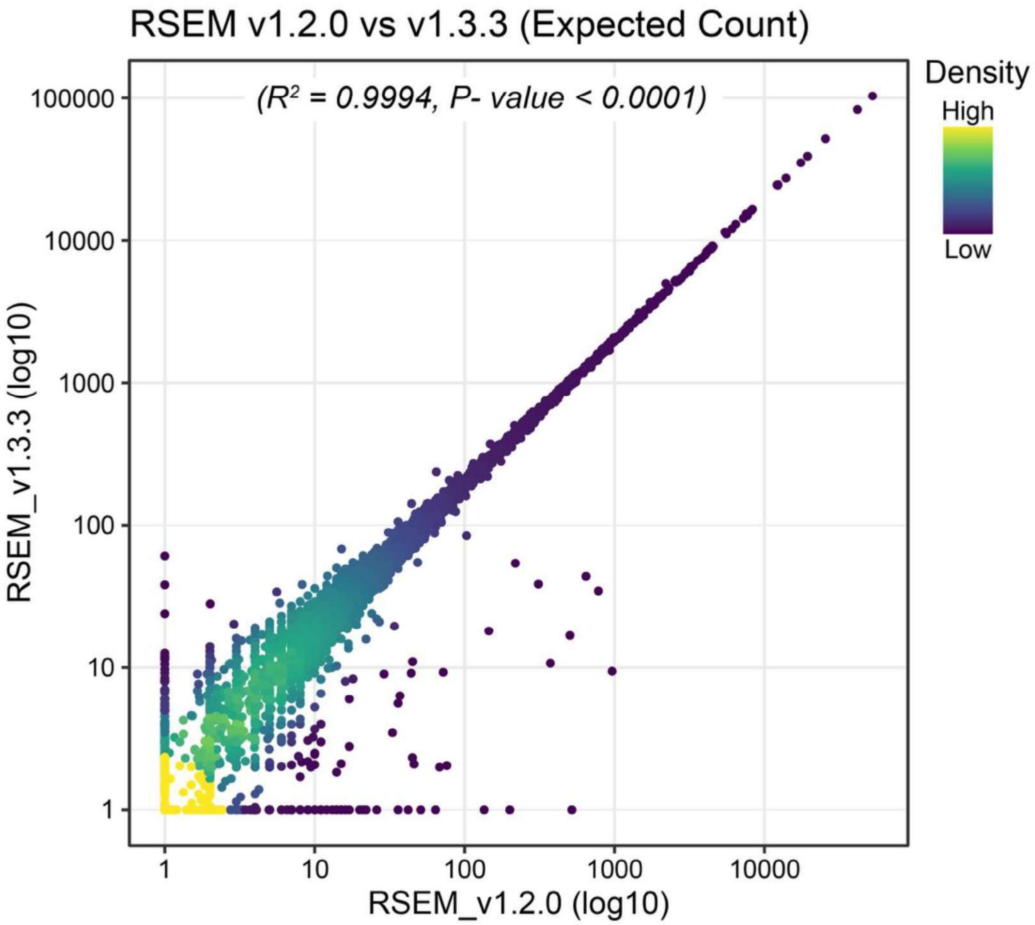

Linear regression analysis of FPKM and expected read counts obtained from the two RSEM versions (v1.2.0 and v1.3.3) demonstrated a high degree of concordance, with an R^2 value close to 1 and a p-value < 0.0001. Note that we show the log transformed values to diminish the impact of outliers (the true linear correlations were even higher).

Basic details of the copy number calling are missing for CNVkit. For example What was the bin size used? What log thresholds and values were applied to call changes? A 5 year old version of CNVkit is still being used here. Was any cohort analysis employed in any of the processing steps of CNVkit? Which segementation algorithm was used?

Before addressing these points, it is important to emphasize that copy number alterations were infrequent in melanocytes from normal skin. Given their rarity, copy number alterations were not discussed in the results and had no relevance to the main conclusions of this study.

Nevertheless, we do call copy number alterations, and as such, these operations deserve additional description in the methods, which we address in this revision (line number: 401-413). Thank you to the reviewer for pointing this out.

I (HS) developed the beta-version of CNVkit[7] and eventually partnered with a software engineer, Eric Talevich, to release a production quality version of the software[8]. At one point, we wrote a DNA nexus app, which became the most widely used third-party app on the entire could based platform. We have not updated the app in years, even though several new versions of the CNVkit software have been released. The reason we have not updated the app in DNAnexus is because the core functionalities of the DNA copy number inference have remained stable. All of the updates in the past 5 years have been to fix niche bugs and/or to add new reporting functionalities, requested by other users, but we do not use the new functionalities ourselves.

The single cell parameters used in Xenium analysis are not provided either for the XOA pipeline or Seurat. It is unclear as to the batch correction, doublet identification mitochondrial percentage checking and basic qc for single cell analysis. Also no details of the Deseq2 for DE-analysis are provided. There are also large gaps in the description of the single cell analysis methodology.

Thank you for this comment. We discuss the analytical workflow in greater detail in this revision. However, the specific suggestions by the reviewer are not applicable to this technology. Xenium is not susceptible to doublets because it performs analyses on cells in situ. The Xenium instrument performs cell segmentation from images (not fluidics). Moreover, Xenium is a probe-based technology that relies on entirely different sets of internal benchmarks than dissociated single-cell sequencing data from 10X chromium. There are no measures of mitochondrial transcripts, but the instrument does include some negative control probes and codewords to measure the specificity of probe detection and deconvolution. In this revision, we summarize some of the key quality control metrics in the methods, and we include summary files, directly output by the Xenium analyzer, in figshare for users who seek a deeper dive into the data. We also made the Xenium data publicly available in GEO.

Additionally, details on the analysis of differential gene expression have been included in the Methods section (lines 717-722), using the publicly available Seurat R toolkit. The parameters used for DESeq2 are specified therein.

These technical and reproducibility concerns cast doubt on the reproducibility of data analysis and must be addressed before publication.

We sincerely thank the reviewer for their thoughtful and detailed evaluation of our manuscript. It is evident that they dedicated considerable time and care in reviewing our results and methods. We greatly value their constructive feedback and believe that their suggestions have significantly strengthened the manuscript.

Reviewer #2 (Remarks to the Author):

This manuscript reports a considerable amount of work, representing an in-depth follow up to the same group's publication in 2020 (Tang et al) and some very interesting conclusions. Their previous work, in essence, found that melanocytes in sun exposed regions of skin carried a much higher mutation load than those from regions not exposed to sunlight.

The approach used allows for a more comprehensive analysis of single melanocytes than single cell RNA sequencing. Single isolated melanocytes are grown on to provide several hundred cells which can be subsequently analysed by DNA and RNA sequencing and morphological analysis. However. as the authors acknowledge, the cells are removed from their environment for numerous cell generations and there is the possibility of changes occurring. Nevertheless a coherent story emerges suggesting this is not an issue.

The authors use a strategy to identify "outlier" melanocytes in skin biopsies with high mutation burden among cells with low burden and vice versa. They suggest low burden cells have been protected from mutation either biologically or physically. What do the authors think about the high mutation cells, some of which appear to have a burden 6 times higher than their neighbouring cells? Are they much older cells that have been in the epidermis longer?

Yes, we believe that the melanocytes with high mutation burdens have been in the epidermis longer, whereas the melanocytes with low mutation burdens spent much of their lives in hair follicles before migrating to the epidermis. Normal skin undergoes repeated cycles of ultraviolet (UV) radiation exposure, the extent of which depends on an individual's sun exposure history. As shown in Figure 6, we suggest that each cycle of UV radiation exposure results in the elimination of some melanocytes due to DNA damage, while others persist. Our findings indicate that melanocytes with a high mutation burden represent long-lived cells that have survived multiple cycles of intense UV exposure. In contrast, melanocytes with a low mutation burden likely correspond to cells that have recently migrated into the epidermis in response to recent UV insults. This process likely continuous, indicated by the presence of intermediate mutation burden melanocytes. This is discussed in lines 274-276 of the manuscript.

Importantly, the revised version of the manuscript includes new Xenium data (Fig. 5, S8 and S9) further supporting this hypothesis.

Supp Table 1 shows the number of successful clones grown for each biopsy. They set a minimum mutation burden of 3 (per Mb?) to be included in analysis. Did they set a lower limit of number of melanocytes analysed per biopsy so there is confidence in the median mutation burden estimate?

Thank you for this question. We did not set a lower limit for the number of cells per donor. When the number of cells sequenced at a site is small, we agree that the median mutation burden is not necessarily a perfect measure of the mutation burden at that site. However, if there is extreme variability in mutation burden across these cells, then we believe it warrants investigation as to why. For example, from donor 7 Lower Back, we only sequenced four cells, but their mutation burdens ranged from 0.4 mutations/Mb to 6.15 mutations/Mb. It is striking to see two cells from the same site with a 15-fold difference in mutation burden and believe that these two cells warrant inclusion in the in the analyses.

Most of the donors are past 60 years of age, but some are younger. Is there any correlation between mutation burden and age, or incidence of low burden cells and age (older people might be expected to have few follicular melanocytes).

Thank you for this excellent question. The mutation burden of skin cells varies at multiple levels: between individuals, across different sites within the same individual, and from cell to cell within a given site. In this manuscript, we focus specifically on cell-to-cell variability within each site. The broader questions of person-to-person and site-to-site variability are also of great interest. In this revision, we have provided metadata to enable others to explore person-to-person differences, and we anticipate publishing follow-up studies examining the factors that drive such variability, including age.

Since the reviewer raised this point, we will share our current perspective on the relationship between mutation burden and age. In our dataset, the correlation between age and mutation burden was weak. However, we believe this reflects sampling bias: many of our younger donors were recruited from a high-risk skin cancer clinic, while our older donors were more representative of the general population, including some with skin of color. We are actively recruiting younger donors who better represent the general population, but such data are not yet available. (It is unexpectedly difficult to obtain normal skin from young individuals without dermatologic concerns, as healthy donors rarely undergo procedures involving skin removal.) For these reasons, we believe it would be premature — and potentially misleading — to draw conclusions about the relationship between mutation burden and age in the current manuscript.

While the relationship between age and mutation burden is not strong, we do see a much higher mutation burden in melanocytes from Caucasians as compared to skin of color as well as a higher mutation burden in melanocytes from people with a history of skin cancer as compared to people without a history of skin cancer. Nevertheless, we think the topic of person-to-person variability in mutation burden would be a better fit for a future manuscript, which includes new data filling in some of the gaps in types of people sequenced.

They carry out differential gene expression between high and low burden melanocytes. Based on transcript analysis the authors suggest low burden melanocytes are more stem-like or neural crest (progenitor like). I would have expected follicle-located (and thus mutation protected) pigment cells to fall into two categories: melanocytes actively synthesising melanin and melanocyte stem cells located in the bulge region. Can they distinguish two populations in the low burden transcript data? Published data suggests they would be DCT positive but KIT, TYRP1 and TYR negative or low. (Of course, it is possible that the bulge stem cells are not identified, or one of the populations is lost somehow)

This is another fantastic question. In this revision, we have added spatial transcriptomics data on the Xenium platform from two new donors. It is not easy to capture cross sections of human hair because the density of hair follicles is low (when compared to animal models) and the follicles are not synchronized at the same stage of growth. As the reviewer is aware, melanocyte stem cells are located in the bulge, and pigment producing melanocytes can be found in the bulb during the anagen (i.e. growth) phase. In our previous submission, we did not capture any hair follicle in the anagen phase. Nonetheless, between the three skin samples, we now have representative sections of all regions of the hair follicle, including the bulb.

Indeed, we observe the outer root sheath of the bulge, isthmus, and suprabulbar regions of the hair follicle have melanocytes in a more stem-like state (expressing our LowMut signature), and the melanocytes in the bulb (especially the cells near and above the dermal papilla) occupy a relatively differentiated state (expressing our HighMut signature). We now discuss this finding in our manuscript (line number: 236-240, Fig. 5B). We commend the reviewer on their shrewd prediction, which was proven true once we captured a hair follicle in the anagen phase of growth!

Comparing gene expression in the two populations with existing datasets they find a significant overlap between high-burden cells and “adult melanocytes” and low burden cells with “melanocyte

stem cells". The P value for the overlaps are very significant, albeit the actual number of transcripts overlapping is small.

This is an astute observation that is due to the fact that the total gene space is large. There are approximately 20,000 RefSeq genes in the genome. The hypergeometric test will calculate the probability of random overlap between two sets of genes from this space. For the LowMut versus Melanocyte Stem Cell comparison, there are 12 genes in the LowMut signature and 1069 genes in Belote's MSC signature. The expected overlap is 12/20,000 x 1,069/20,000 x 20,000 = 0.64 genes. We observed 7 genes overlapping, which is unlikely to happen by random chance and thus extremely significant. Even if we adjust assumptions to be more conservative (e.g. by reducing the gene space to 10,000 genes, which is roughly the number of genes expressed in a given cell lineage), the overlap remains significant.

Further evidence as to the "stem like" characteristic comes from cell morphology analysis and their finding that high burden cells are more dendritic, low burden cells more ovoid, fitting observations by others that bulge localized stem cells are more ovoid.

Finally using spatial transcriptomics and transcript signatures that identify their two melanocyte classes they show most low burden melanocytes are either in the hair follicle or in the epidermis close to follicles. The morphology of the skin sections is not great, and it is hard to know if the low burden cells they are identifying in the follicle are located in the bulge. They seem to be distributed throughout. I would have expected some differentiated cells to be localized at the follicle base. Where are the few high-burden signature cells in the follicle localized? Along those lines are there any low burden (UV protected) cells with a high burden signature which might correspond to differentiated follicular melanocytes? (see in this context my comment above about two populations in the follicles)

The reviewer asks a fantastic question, and their prediction was 100% accurate. In this revision, we added new Xenium data from two additional patients. In one patient, we got a full-length cross section of a human hair follicle in the anagen phase. We were able to clearly see a population of differentiated melanocytes in the bulb of the hair follicle. These melanocytes were marked as "HighMut" cells – not because they have high mutation burdens per se but because they express the same differentiation expression signature as melanocytes with high mutation burdens. The new data is now shown in figure 5, S8 and S9 (line number 217-221, 604-607, 700-732).

A minor point; they find HERC2 to be preferentially expressed in high mutation burden melanocytes. They comment (page 4) that HERC2 is well established as genetically associated with pigmentation. This is true, but it is believed that the genetic association is via an intronic SNP that is regulating the expression of adjacent OCA2.

Thank you for raising this point. We have removed the discussion of HERC2 in this revision.

Overall, thank you to the reviewer for the sharp questions. We believe the manuscript is significantly strengthened because of your feedback.

Reviewer #3 (Remarks to the Author):

The study aimed at characterizing subpopulations of melanocytes using clonal expansion and multi-Omics analysis, identifying distinct high- and low-mutation melanocytes, in human skin tissue. Individual melanocytes from donors were harvested, clonally expanded, and subjected to DNA sequencing (exome sequencing) and RNA-Seq. An additional skin samples was further imaged using

a workflow involving Xenium to localize melanocytes with low- and high-mutation burden, based on a derived gene-expression signature.

General comment: The study's aims are of interest as many aspects of melanoma biology are yet to be explored using new (single) cell technologies. The approach is robust (like clonal expansion and sequencing) and the bioinformatics methods are sound. The use of the Xenium methodology to identify the mutational status of melanocytes via their gene expression signature is innovative.

There are some major drawbacks:

1. The threshold defining high- and low-mutation melanocytes is not well justified (Figure 1). The authors observed a difference in mutation burden across melanocytes between participants and decided to implement a relative, rather than absolute mutation level threshold. The relative threshold defines high-mutation melanocytes as those in the upper quantile vis a vis melanocytes from the same participant. That way, the number of observed mutations defining a high-mutation melanocyte differs from participant to participant. An absolute threshold would apply the same mutation level to any melanocyte. There is no biological justification for this approach, particularly as the authors claim that melanocytes exit the stem cell niche after accumulating a certain number of mutations. It is unclear why this number would differ from participant to participant, or from body site to body site. Did the authors try to define an absolute threshold. Did the analysis and conclusion hold?

We apologize for not describing in greater detail how we arrived at the thresholds to define high- and low- mutation burden melanocytes. This is a critical question, for which we spent considerable amount of time researching before settling upon the criteria used in this manuscript.

In this revision, we include a new supplementary figure (supplementary figure S3) that summarizes the different criteria that we tested (reprinted below for the reviewer's convenience). We include the reviewer's suggestion of an absolute threshold among the conditions tested. Overall, it was encouraging to observe that the differentially expressed genes remained reproducible across the various threshold conditions, including the reviewer's suggestion.

However, a notable limitation to using absolute cutoffs, as the reviewer suggests, is the large number of genes that were considered significant. We are worried that these conditions were picking up person-to-person differences in addition to cell-to-cell differences. Under the reviewer's conditions, certain donors contributed to an outsized proportion of either low- or high- mut melanocytes. By contrast, our solution (to use relative mutation burdens within each sample) maintained a similar representation of donors in each group, thereby diminishing the likelihood of picking up on donor-to-donor differences in gene expression.

In summary, we selected the final criteria with four considerations in mind: (1) balancing thresholds to avoid definitions that were either too lenient or overly stringent, (2) preventing overrepresentation of subpopulations derived from only a few biopsies, (3) preventing overrepresentation of specific patients in either the HighMut or LowMut groups, and (4) ensuring robust and reproducible gene expression profiles. Accordingly, the chosen thresholds, met all these criteria (line number: 484-495).

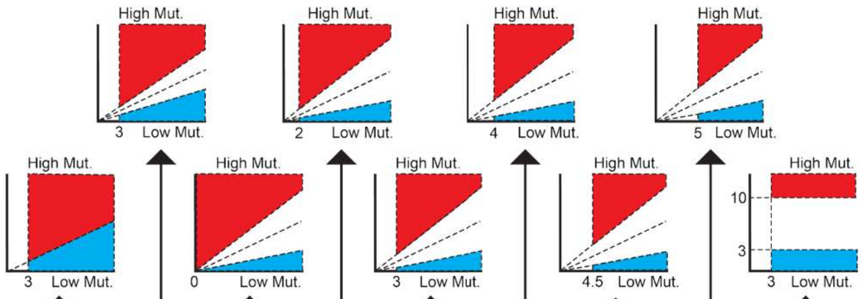

X-axis cutoff:	Biopsy Mut/Mb	3	3	0	2	3	4	4.5	5	3	Total geneset
Y-axis cutoff:	Low mut. Mut/Mb	< Median	1st tertile	1st quartile						< 3	
	High mut. Mut/Mb	> Median	3rd tertile	4th quartile						> 10	
Genes enriched in high mut. melanocytes	AHNAK2	x	x	x	x	x	x	x	x	x	9
	ATOX1		x	x	x	x	x	x	x	x	8
	HLA_DPA1	x	x		x	x	x	x	x	x	8
	LIPA			x	x	x	x	x	x	x	7
	DPP6			x	x	x	x	x	x	x	7
	HERC2			x	x	x	x	x	x	x	7
	AK5			x	x	x	x	x	x		6
	EPS8				x	x	x	x	x	x	6
	SH3BGRL3		x			x	x	x	x	x	6
	FRG2DP					x	x	x	x	x	5
	SLC43A3			x	x	x	x				4
	PRR13				x	x	x	x			4
	CD109					x	x	x	x		4
	ABCC2				x	x	x			x	4
	HMOX1					x	x	x		x	4
	FTH1P10		x			x	x			x	4
	MAP1LC3B	x	x			x				x	4
	AC026785.2				x	x	x				3
	MC1R					x	x		x		3
	SCD					x	x			x	3
	ARHGAP4					x				x	2
	AC016831.1					x				x	2
	ANKRD37					x					1
Genes enriched in low mut. melanocytes	SGCE	x	x		x	x	x	x	x	x	8
	PSME1			x	x	x	x	x	x	x	7
	ITM2A		x		x	x	x	x	x	x	7
	PALLD			x	x	x	x	x	x		6
	MYL9			x	x	x	x	x	x		6
	NTNG1			x		x	x	x	x	x	6
	IL24	x			x	x		x	x	x	6
	MYLK				x	x	x	x	x		5
	RGMB				x	x	x	x	x		5
	FAM207A			x	x	x	x			x	5
	VCAN	x			x	x	x	x			5
	TAGLN	x			x	x		x	x		5
	HACD1					x	x	x	x	x	5
	TCF4					x	x	x	x	x	5
	ARHGDIB					x	x	x	x	x	5
	FBN1	x	x		x	x		x			5
	LMCD1				x	x			x		3
	DAAM2					x				x	2
	SEMA3C					x					1
	Additional DE genes	17	14	6	10	0	8	10	16	742	

Final definition of high- and low- mutation burden cells, used in this study

Reviewer table. Alternative definitions of high- and low- mutation burden melanocytes capture biologically similar cells. Melanocytes were gated into high- and low- mutation burden cells based on their absolute mutation burden and/or their relative mutation burden within a biopsy. After gating, differential gene (DE) expression analyses were performed, comparing melanocytes with high- vs low- mutation burdens. The differentially expressed genes under our final condition are shown above, and we indicate when these genes were also found with an alternative definition of high- and low- mutation burden melanocytes. The number of additional differentially expressed genes, found with alternative analyses, is indicated in the bottom row.

2. There is no validation of the gene expression signature derived from low- and high-mutation melanocytes. This signature is crucial for the Xenium experiment, but its robustness is not demonstrated. The authors should perform a cross-validation experiment where some melanocytes are not included in the training and derivation of the signature. During testing, hold-out melanocytes would be used to test the validity of the signature, i.e. whether the signature correctly identifies whether the hold-out melanocytes are of low- or high-mutational nature.

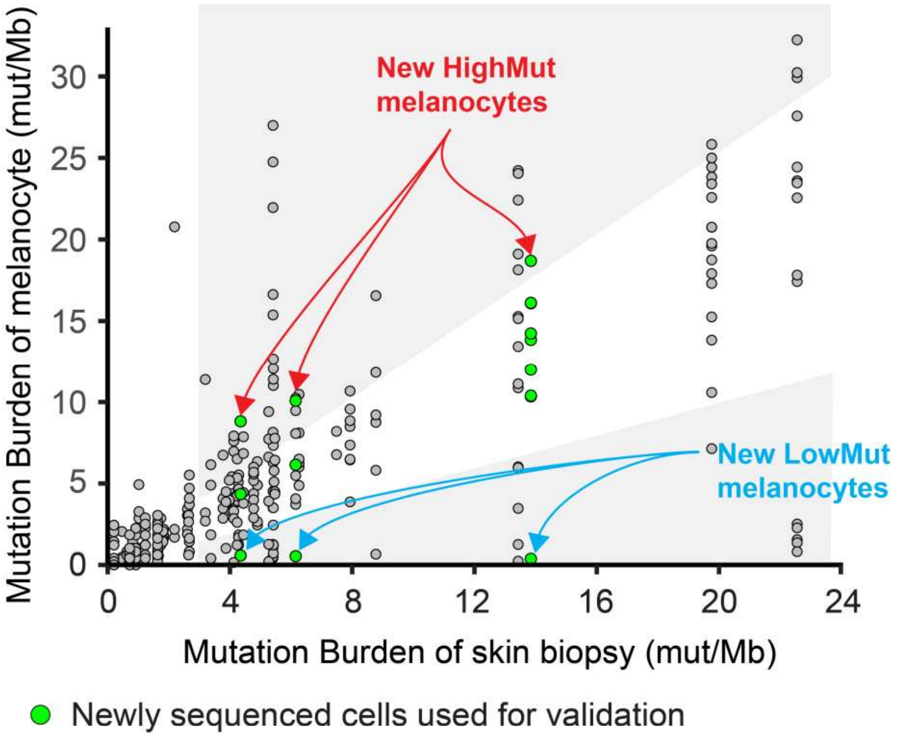

The reviewer raises an important matter. At this point, we cannot go back and “hold out” samples that were used to define the low- and high- mut gene expression signatures. However, since our last submission, we have genotyped additional melanocytes from three new skin samples. This provided a true validation cohort, which included three new HighMut melanocytes and three new LowMut melanocytes, as shown in the figure on the upper right (figure S4, line number: 139-143, 567-576).

We were encouraged to see that the new cells have concordant gene expression profiles as the High- and Low- Mut melanocytes from the discovery cohort, indicated by their clustering in the UMAP on the right (note that in the UMAP on the right. We include the plots above, plus additional data, in a new supplementary figure, showing validation data.

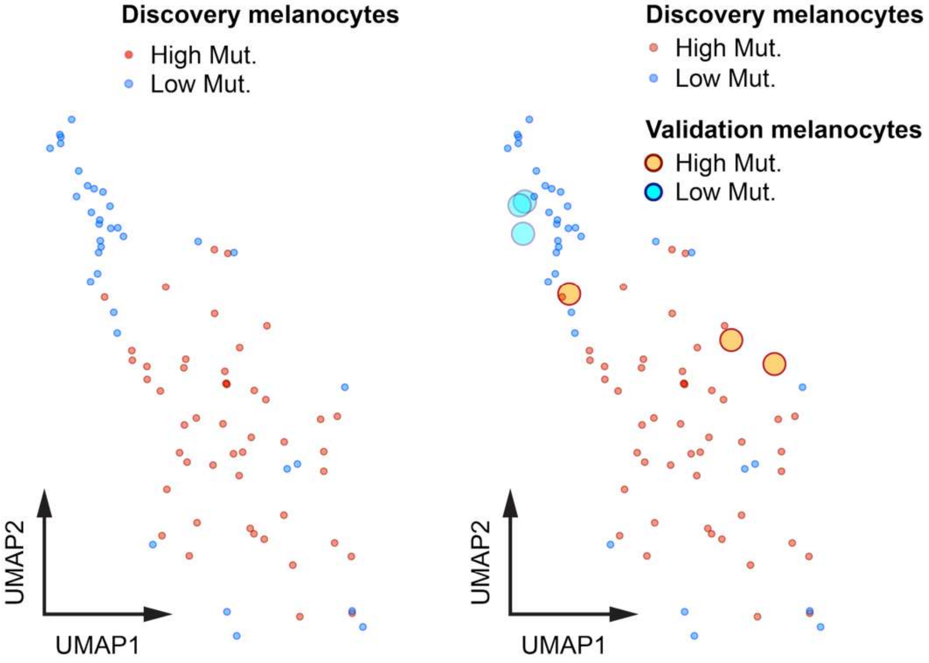

As a limitation to these analyses, the number of validation cells was small. It is laborious to discover new melanocytes that fit our definition of HighMut and LowMut cells (note that most of the newly sequenced cells fell in the intermediate category).

To complement the validation data above, we also generated new Xenium data, as discussed in response to the reviewer’s next point.

3. The Xenium experiment is conducted in a single participant only. The uncertain robustness of the signature (see #2) might necessitate to use of more participants (unless the signature is close to 100% correct).

Thank you for this suggestion! We included new Xenium data on skin from an additional 2 patients. The new skin samples show the exact same distributions of low- and high- mut melanocytes! The new data is shown in the updated version of figure 5, S8 and S9. At this stage, extensive orthogonal evidence, genomic, transcriptomic, morphologic, and spatial, derived from multiple technologies and a large number of unique donors, supports the existence of two distinct melanocyte subtypes in human skin.

4. It is unclear why the authors decided to perform exome rather than whole genome sequencing, which would have allowed for the derivation of a more robust mutational estimate (i.e. more genome real estate and chance to observe mutations). Was it because DNA from only 230 cells (per colony) were used? Why did the authors limit colony growth to that number?

Thank you for this question. With any sequencing project, there is a tradeoff between the number of samples, depth of sequencing, and breadth of sequencing. For this project, we prioritized number of samples and depth of sequencing over breadth of sequencing. While the cost of sequencing has diminished since the inception of next generation sequencing, it remains expensive to sequence the genomes of hundreds of samples, and keep in mind that we also performed whole transcriptome sequencing on each clonal expansion, further adding to the costs.

Sequencing at a high depth was especially critical for this study. We achieved 78X coverage in our whole exome sequencing data, which is higher than most papers that perform whole exome sequencing. The high coverage allowed us to accurately measure the allele frequencies of variants, which was helpful in distinguishing somatic mutations from artifacts. It would have been cost prohibitive to sequence whole genomes at this depth.

As the reviewer points out, the main advantage of whole genome sequencing is to detect more mutations per cell. Since skin cells have high mutation burdens, we can arrive at a robust estimate of mutation burdens from whole exome sequencing data. In adjoining reviewer figure, we show a bar graph of mutation burdens from select cells with high, intermediate, and low mutation burdens. The error bars correspond to confidence intervals, calculated with the Poisson test based on the number of events (absolute mutations) per interval (megabases of genome). At these confidence intervals, we can easily discriminate between cells with high- vs low-mutation burdens for this study.

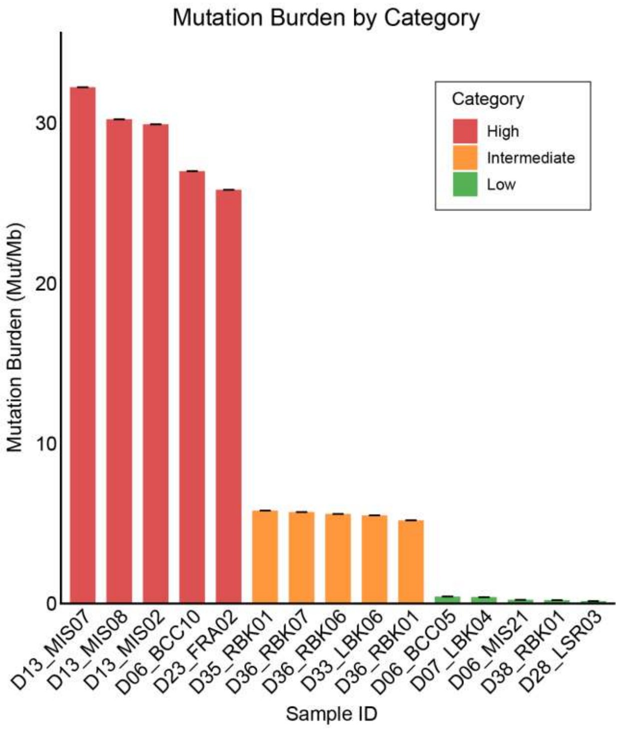

The number of cells per colony did not factor into our decision to perform exome sequencing. Since their genomic DNA is amplified, we have enough amplified DNA to sequence at any footprint.

The reviewer also asked why we harvested colonies once they reach ~200 cells. We monitor colony growth on a daily basis and harvest when we noticed growth beginning to slow. If we could expand the colonies further, we would, but cells from normal tissues have limited replicative potential. We discuss possible reasons why in line number 331-343 of the manuscript.

In summary, we thank the reviewer for their shrewd observations. We completely agree with their points and addressing them has improved the manuscript.

References

1. Yoshida, K. et al. Tobacco smoking and somatic mutations in human bronchial epithelium. Nature 578, 266–272 (2020).

2. Machado, H. E. et al. Diverse mutational landscapes in human lymphocytes. Nature 608, 724–732 (2022).

3. Park, S. et al. Clonal dynamics in early human embryogenesis inferred from somatic mutation. Nature 597, 393–397 (2021).

4. Tang, J. et al. The genomic landscapes of individual melanocytes from human skin. Nature 586, 600–605 (2020).

5. Bohrson, C. L. et al. Linked-read analysis identifies mutations in single-cell DNA-sequencing data. Nat. Genet. 51, 749–754 (2019).

6. ICGC/TCGA Pan-Cancer Analysis of Whole Genomes Consortium. Pan-cancer analysis of whole genomes. Nature 578, 82–93 (2020).

7. Shain, A. H. et al. The Genetic Evolution of Melanoma from Precursor Lesions. N. Engl. J. Med. 373, 1926–1936 (2015).

8. Talevich, E., Shain, A. H., Botton, T. & Bastian, B. C. CNVkit: Genome-Wide Copy Number Detection and Visualization from Targeted DNA Sequencing. PLoS Comput. Biol. 12, e1004873 (2016).

9. Talevich, E. & Shain, A. H. CNVkit-RNA: Copy number inference from RNA-Sequencing data. bioRxiv 408534 (2018) doi:10.1101/408534.

Response to Additional Reviewer Comments:

Reviewer #1:
Remarks to the Author:

The authors have thoroughly revised and substantially improved the manuscript. The key point about the cut off between low and high mutated melanocytes has been well addressed, as have concerns about the spatial analysis. The informatics is now much better explained and the choices made have been justified. Overall, this study adds to our knowledge of human (as opposed to mouse) skin biology and will be of interest in the fields of epidermal biology and carcinogenesis. Minor point: “melanocytes in the hair follicle occupy a privileged niche, protected from UV radiation, but periodically migrate out of the hair follicle to replenish the interfollicular epidermis after waves of photodamage’…This is a possible but unproven explanation. Most photodamage is low doses of UV, which may not trigger such migration. Equally other epidermal injuries may result in migration. Perhaps move this speculation from abstract to discussion?

We thank the reviewer for their careful reevaluation of the revised manuscript and for the positive assessment of the changes made. We are grateful for the reviewer’s detailed and constructive comments throughout the review process, which have substantially improved the clarity, rigor, and presentation of this work. We also appreciate the reviewer’s acknowledgment that the key concerns raised previously have now been addressed.

With respect to the comment regarding the statement that melanocytes in the hair follicle occupy a privileged niche protected from UV radiation and may migrate to replenish the interfollicular epidermis following photodamage, we agree that direct experimental evidence for UV-induced migration in human skin remains limited and that other forms of epidermal injury may also contribute to melanocyte redistribution.

Our data identify a population of low-mutation burden melanocytes enriched within the hair follicle, a finding that is consistent with the hair follicle representing a relatively protected niche in human skin. Because this interpretation is directly motivated by the observed mutational and spatial patterns in our dataset, we have retained this concept in the abstract. However, in response to the reviewer’s suggestion, we have revised the wording to more clearly frame this statement as an inference supported by our data rather than a definitive mechanistic conclusion, and we have expanded the discussion section to explicitly acknowledge alternative explanations and current limitations.

We thank the reviewer again for their insightful feedback, which has played an important role in strengthening the manuscript.

Additional comments on the remaining points by reviewer #3
Reviewer #3 highlights that issues remain about the definition and validation of high- and low-mutation melanocytes. This is a critical issue for the paper and the comment ‘…there is a definite need to show numerically the validity / robustness of the proposed list of differentially expressed genes’ should be addressed. The additional experiment provided for validation is not convincing as ‘not high-mutation validation melanocytes do not convincingly fall within the range of the discovery melanocytes’. As suggested a computational approach may help confirm the robustness of the definition of high- and low- mutation cells.

We appreciate this comment and have addressed it as detailed in our response to Reviewer 3 below.

Remarks on code availability:
Code is now accessible and is much better explained.

We thank the reviewer for noting the improvements in code accessibility and documentation. We are glad that the revisions have made the code clearer and more usable.

Reviewer #2:
Remarks to the Author:
This revised manuscript deals with the concerns I had with the initial submission. I believe there are interesting and important conclusions drawn. In particular the identification of putative stem cells or melanocyte progenitors in the epidermis. Previous studies have shown migration of such progenitors from hair follicle to epidermis in melanocyte-depleted mouse skin and in vitiligo skin but the work described here shows progenitors present in non-pathological samples.

Remarks on Protocol(s):
Concerns of other reviewers are around bioinformatic methods. This is not my expertise.

We thank the reviewer for their positive assessment of the revised manuscript and for highlighting the significance of our findings, particularly regarding the identification of a melanocyte subpopulation with stem cell-like characteristics in normal human epidermis.

Reviewer #3:
Remarks to the Author:
Summary: The revision addresses the reviewer‘s concerns to some degree (more Xenium experiments, experiments aimed at validating the gene expression signature of high- and low-mutation melanocytes). However, there are still some questions with regard the definition of high- and low-mutation melanocytes; and the validation experiments do not provide a numerical assessment of the validity of the gene expression signature. As the whole study hinges on the validity of that signature, I don‘t believe the study is ready for publication quite yet. The authors should find a way to numerically validate the signature - see comment 2. Given such an assessment, the question of the correct definition (comment 1) becomes less relevant.

Comment 1. The first question referred to the definition of high- and low-mutation melanocytes, and the resulting set of differentially expressed genes. The authors provide additional data (supplementary figure S3). The authors show that - irrespective of the exact definition (i.e. fixed threshold vs quantiles) -the set of differentially expressed genes between high- and low-mutation melanocytes overlap considerably. However, the number of non-overlapping genes is also high, particularly for the fixed mutation definition (last column, n=742 non overlapping genes). Table S3 thus does NOT settle the question whether the authors used the correct definition (i.e. quantiles). The authors claim to have chosen an approach / definition for „ensuring … reproducible gene expression profiles“. As discussed (Comment 2) below, this has only been partially demonstrated.

Thank you for this comment. The reviewer had previously suggested an alternative definition of high- and low-mutation-burden melanocytes based on an absolute mutation burden cutoff rather than a relative cutoff. We had considered this approach prior to the reviewer’s suggestion but ultimately decided against it. Mutation burden in melanocytes is strongly influenced by heritable traits, such as skin tone, and environmental exposures, such as cumulative sun damage. However, the goal of this study was to understand how melanocytes from the same tissue (i.e. sharing genetic background and environmental exposures) acquire different mutation burdens. Our definition is better suited to capturing within-tissue differences. For example, under the reviewer’s approach, all melanocytes from a minimally sun-exposed donor could be classified as low-mutation-burden cells, whereas our approach identifies cells with abnormally high (or low) mutation burdens within the same tissue.

Consistent with this reasoning, when we applied the reviewer's definition (Fig. S3), we recovered most of the same genes identified by our analysis but also identified an additional ~750 genes associated with other confounding biological processes. For these reasons, our definition is better suited to address the goals of this study.

Comment 2. The second question is a most crucial one. The authors were asked to valide the list of differentially expressed genes via cross-validation. The authors refrained from doing so, arguing that the list has already been defined (i.e. all data has been used to define the list). While I see this point, there is a definite need to show numerically the validity / robustness of the proposed list of differentially expressed genes. (A) An alternative to cross-validation is bootstrapping, which could show - for example - how often (% of bootstrap samples) a particular gene is among the list of differentially expressed genes. Bootstrapping would not affect the already established list of genes. (B) The authors provide another alternative - validation via a newly set of sequenced melanocytes. Unfortunately, data from only 6 out of 13 newly sequenced melanocytes were useful for further analysis (due to the strict „quantile“ definition of high- and low-mutation melanocytes; all melanocytes could have been used in the „threshold“ definition). The authors do not perform a differential expression analysis on these 6 samples (why? - this would have been, even if suboptimal, a way to show numerically the robustness of the signature) and provide, instead, a heatmap and UMAP of the expression profiles of the six melanocytes. The heatmap seems to show an overall TREND that the validation melanocytes show similar profiles than the discovery set. The UMAP shows good concordance with the low-mutation validation melanocytes, but the high-mutation validation melanocytes do not convincingly fall within the range of the discovery melanocytes. In summary, the authors do not provided a numerical assessment of the robustness of the gene expression signature, but a „visual“ - only partially convincing - assessment instead (heatmap/UMAP).

The reviewer had previously suggested an alternative method to define genes upregulated in high vs. low mutation burden melanocytes. In addition to performing differential gene expression analysis one time on the full cohort of cells (what we did in the manuscript), they suggested to assess the robustness of the proposed list by showing how often the genes we defined would have been discovered in different subsamples of the full cohort, through bootstrapping or cross-validation. We have now done this (see attached) and would be happy to include the results in the manuscript. As you can see, the genes defined by our approach were also the most frequent genes to come up as significant in the various cross-validation training folds, confirming the robustness of the proposed list. Overall, had we taken the reviewer's approach from the beginning, we would have settled on a nearly identical list of genes.

Comment 3: I congratulate the authors for adding additional Xenium experiments.

We thank the reviewer for their kind comment and for acknowledging the addition of the Xenium experiments. We are glad that these experiments strengthen the manuscript.

Comment 4: While I see the argument of overall costs, WGS costs have come down considerably and I don‘t see the need for deep sequencing in cell lines of clonally expanded melanocytes, i.e. it should be possible to detect somatic mutations even at low sequencing depth.

We thank the reviewer for this comment and for highlighting the considerations around sequencing depth. We agree that whole-genome sequencing costs have decreased and that somatic mutations can be detected at lower coverage. However, as advised by the editor, we believe that the current sequencing depth is sufficient for the goals of our study and that no additional deep sequencing is required.

ADDITIONAL COMMENTS ON THE AUTHORS' RESPONSE TO MY POINTS ABOVE
I can definitely accept the arguments brought forward regarding point #1.

I am glad that the authors provide a numerical assessment of the robustness of the signature (point #2). This strengthens the paper a lot. I do not have access to the exact methods - but the results look convincing, i.e. the chosen gene list seems very robust. A little remark: The author use a threshold of p_adj<0.2 and p_adj<0.1; these thresholds are typically used for the FDR (not p-value); so I am not quite sure whether the notation is correct.

We thank the reviewer for their positive assessment of the robustness analysis and for highlighting this point. We would like to clarify that the thresholds we report (padj < 0.2 and padj < 0.1) correspond to Benjamini–Hochberg false discovery rate (FDR)–adjusted p-values as output by DESeq2. That is, the padj values are FDR-corrected p-values, so our notation reflects the adjusted p-values rather than raw p-values. To address this comment and avoid confusion, we have updated the text to explicitly refer to FDR–adjusted p-values at the relevant thresholds.

We are grateful to Reviewer 3 for their detailed and constructive feedback, which has played an important role in enhancing the rigor and presentation of our work.
